# Using Make for Reproducible and Parallel Neuroimaging Workflow and Quality-Assurance

**DOI:** 10.3389/fninf.2016.00002

**Published:** 2016-02-02

**Authors:** Mary K. Askren, Trevor K. McAllister-Day, Natalie Koh, Zoé Mestre, Jennifer N. Dines, Benjamin A. Korman, Susan J. Melhorn, Daniel J. Peterson, Matthew Peverill, Xiaoyan Qin, Swati D. Rane, Melissa A. Reilly, Maya A. Reiter, Kelly A. Sambrook, Karl A. Woelfer, Thomas J. Grabowski, Tara M. Madhyastha

**Affiliations:** ^1^Department of Radiology, University of WashingtonSeattle, WA, USA; ^2^Department of Clinical Psychology, University of California, San Diego/San Diego State UniversitySan Diego, CA, USA; ^3^Department of Medicine, University of WashingtonSeattle, WA, USA; ^4^Department of Psychology, University of WashingtonSeattle, WA, USA; ^5^Department of Neurology, University of WashingtonSeattle, WA, USA

**Keywords:** neuroimaging pipelines, workflow, quality assurance, reproducibility

## Abstract

The contribution of this paper is to describe how we can program neuroimaging workflow using Make, a software development tool designed for describing how to build executables from source files. A makefile (or a file of instructions for Make) consists of a set of rules that create or update target files if they have not been modified since their dependencies were last modified. These rules are processed to create a directed acyclic dependency graph that allows multiple entry points from which to execute the workflow. We show that using Make we can achieve many of the features of more sophisticated neuroimaging pipeline systems, including reproducibility, parallelization, fault tolerance, and quality assurance reports. We suggest that Make permits a large step toward these features with only a modest increase in programming demands over shell scripts. This approach reduces the technical skill and time required to write, debug, and maintain neuroimaging workflows in a dynamic environment, where pipelines are often modified to accommodate new best practices or to study the effect of alternative preprocessing steps, and where the underlying packages change frequently. This paper has a comprehensive accompanying manual with lab practicals and examples (see Supplemental Materials) and all data, scripts, and makefiles necessary to run the practicals and examples are available in the “makepipelines” project at NITRC.

## Introduction

A major problem in neuroimaging is generating and executing complicated sequences of processing steps (workflows, or pipelines). Scientific rigor demands that these workflows be reproducible (in the sense of being able to replicate published results using the same data and analytic methods, Peng, 2009, 2011). The reality of neuroimaging requires they be parallelizable, fault-tolerant, and easily modified. Although, the scale with which raw data sets can grow is typically limited by the scanner capacity and the financial costs of studies, improvements to scanner acquisition and to data processing algorithms require increasingly more storage, memory, and processing power. To complete in a reasonable timeframe, many analyses require parallelization, demanding more computing and memory resources than are typically available on a standalone workstation, so pipelines must be able to exploit a shared memory multiprocessor or a cluster. A general purpose solution to this problem is not feasible at this point, because standards for data analyses in this relatively young field are constantly being challenged or improved by an active methods community. Like others, our perspective is that we should constantly evaluate novel methods and incorporate useful additions into our pipelines. This often requires combining tools from multiple software packages and tailored scripts. Practically, this task is challenging because existing software packages were developed largely in isolation from each other, which have their own file structures, formats, and naming conventions.

This is the motivation for developing customizable neuroimaging workflow systems (e.g., Nipype, Gorgolewski et al., 2011, and LONI Pipeline, Rex et al., 2003) that allow scientists to incorporate the best algorithms from multiple standard packages as well as custom tools into a single workflow. At the same time, many scientists who use neuroimaging in their research are not skilled programmers, and rely primarily upon mainstream software packages written by others, with limited customizability (e.g., FSL and SPM graphical user interfaces, and AFNI python scripts).

The contribution of this paper is to describe how we can program neuroimaging workflow using Make, a software development tool designed for specifying how to create executable programs from source files. We show we can achieve many of the features of more integrated neuroimaging pipeline systems such as Nipype and LONI pipeline, including reproducibility, parallelization, fault tolerance, and quality assurance reports. The reason for considering this approach is because it reduces the technical skill and time required to write, debug, and maintain neuroimaging workflows, as compared to workflow systems that incorporate layers of abstraction (i.e., “wrappers”) around neuroimaging tools. This in turn reduces the cost and time associated with scientific progress in a dynamic environment, where pipelines are often modified to accommodate new best practices or to study the effect of alternative preprocessing steps, and where the underlying packages change frequently.

This paper has an accompanying manual with lab practicals and examples (see Supplemental Materials) and all data, scripts and Makefiles necessary to run the practicals and examples are available in the “makepipelines” project at NITRC.

## Programming skill vs. flexibility

**Figure 1:**
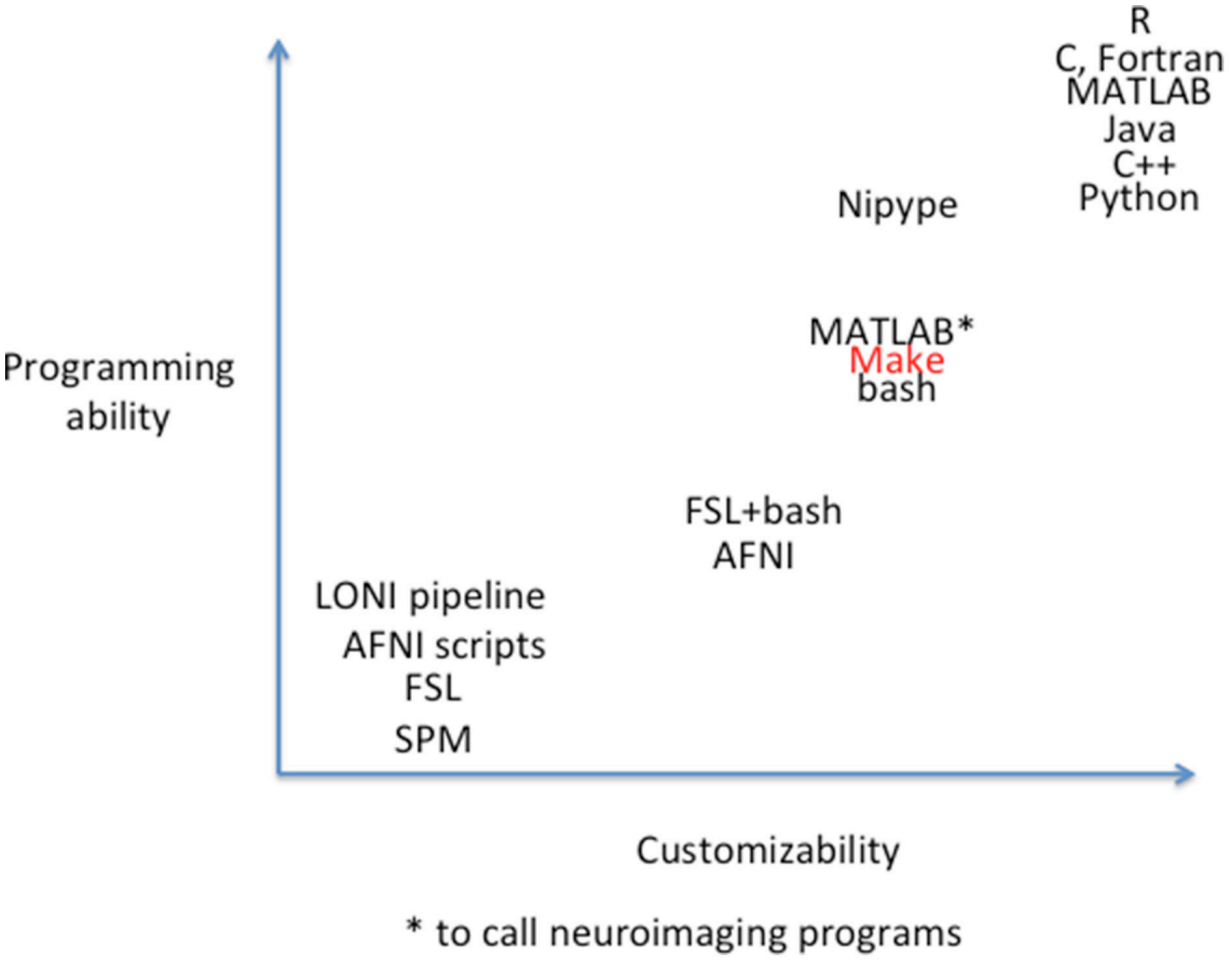
**Conceptual two dimensional space of pipeline tools**. In one dimension is customizability; how possible is it to modify the pipeline to include new neuroimaging processing algorithms? In the second dimension is programming ability. How much programming skill does it take to modify the pipeline? Relative programming ability is estimated based on our collective lab experience and current salaries for skills, where available (Dice.com., 2015). Customizability is estimated based on our lab experience.

Because Linux is the most popular neuroimaging platform (Hanke and Halchenko, 2011; Halchenko and Hanke, 2012; Halchenko et al., 2014), most neuroimaging applications and utilities run on Linux and can be called from bash scripts. For some popular neuroimaging packages (e.g., FSL, AFNI) it is necessary to be familiar with bash, both to run programs and to make contact with existing documentation from the software developers and other lab groups. At one extreme, nonprogrammers can use graphical user interfaces provided by the tools. At the other, sophisticated users can develop new algorithms to supplement or replace processing steps in higher level scripting or compiled languages (e.g., MATLAB, Python, C). In between, scientists may write scripts using bash and other Linux utilities, or parallelize execution of neuroimaging commands through MATLAB's Parallel Computing Toolbox if this resource is available through a user's institution. There is a tradeoff between programming ability and customizability (see Figure 1)^1^. To allow combination of tools from a variety of commonly available packages, systems such as Nipype (Gorgolewski et al., 2011) and LONI pipeline theoretically allow implementation of flexible, modular components without knowing the particulars of each underlying tool. These systems “wrap” components from other packages to provide a consistent interface, data provenance, and parallelism. This allows creation of canned pipelines that incorporate these features and can be executed as easily as other pipelines that provide these features within a specific software package (e.g., FSL's FEAT, SPM's batch). However, the programming skill required to wrap new modules and incorporate those into pipelines is still on par with the difficulty of the wrapping language.

Make is a simple system that can be used for writing and maintaining customizable pipelines that requires less skill and time than any pipeline system that wraps the underlying neuroimaging tools. Make is included in all standard Linux distributions (and is available for OSX and all other UNIX-based systems), so there is nothing additional to install. Make requires no additional code, and thus programmer time, to wrap neuroimaging packages. Wrappers require users who wish to modify pipelines to understand the calling syntax of the packages in addition to the language and software design of the wrapper. While operating without wrappers means that the idiosyncrasies of the underlying packages are not hidden from the user, the calling syntax and program options of the underlying packages are clearly exposed. This makes debugging specific steps easier, because code can be cut and pasted into the shell. This also helps when versions of the underlying programs change, because wrappers do not have to be changed to accommodate version changes in syntax. Anything that can be called from the shell can be included in a makefile.

Using Make for neuroimaging workflow occupies an important space in the tradeoff between customizability and programming ability. As shown in Figure 1, it provides the customizability and reproducibility available from a script in addition to parallelism and fault tolerance. It represents only an incremental increase in customizability and programming ability from bash shell scripting, but a greatly advanced capacity to process large data sets, and thus may address a need of the larger neuroimaging community.

## Implementation

Make refers to a development tool created for expressing a directed acyclic dependency graph (Feldman, 1979). A makefile (or a file of instructions for the progam make) consists of a set of rules that create or update target files if they have not been modified since their dependencies were last modified. Rules take the form:


  target: dependency_1 ... dependency_n
      recipe


The target and dependencies are normally files, and the recipe is a sequence of shell commands (where each line begins with a TAB character) that are executed to produce the target file from the dependencies. To understand the use of Make in a neuroimaging example, consider the simple case of skull stripping a T1 weighted image called T1.nii.gz. The skull stripped file will be called T1_brain.nii.gz, following FSL conventions. The command to perform this skull stripping in FSL is called bet. We create a file, named Makefile, which contains the following lines (Example 1A):

**Example 1A:**
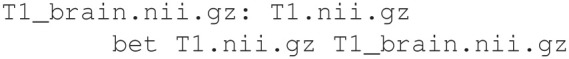
**Makefile for skull stripping**.

Having created this file, the command make will perform the operations necessary to skull strip the T1 image if it happens to be newer than T1_brain.nii.gz, or if T1_brain.nii.gz does not exist. Although, this is a simple example, multiple rules can be included in a makefile that, if chained together, form a directed acyclic dependency graph that can be arbitrarily complex. By comparison, we illustrate the equivalent workflow in Nipype, a wrapper-based workflow (Example 1B). Note that calling a module is similarly straightforward, albeit abstracted from the original command. However, creating a wrapper (Example 1C), which is required to add any new tool to a workflow, requires substantial knowledge of the wrapping language and the workflow conventions (e.g., InputSpec, TraitedSpec classes) in addition to understanding the syntax of the underlying command line tool.

**Example 1B:**
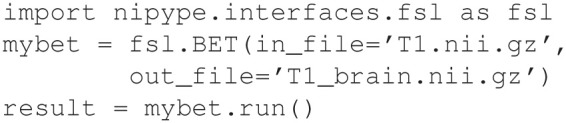
**Nipype command for skull stripping**.

**Example 1C:**
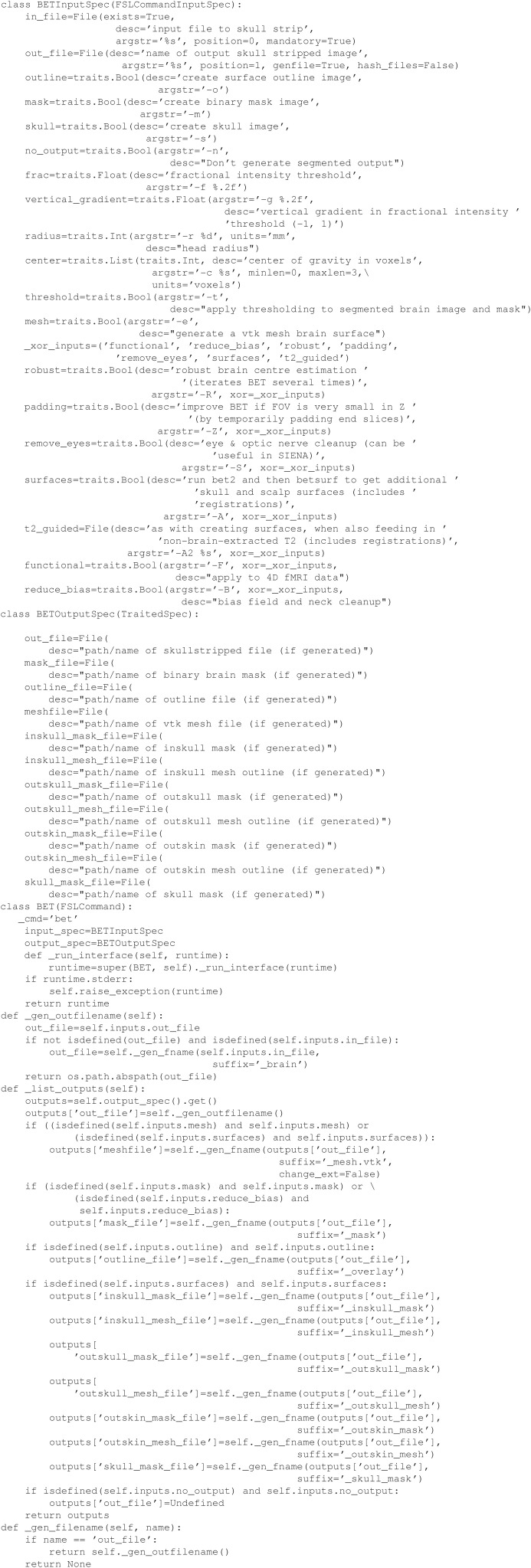
**Nipype wrapper for skull stripping**.

Example 2 shows a slightly more complicated makefile that uses the skull stripped T1 to perform hippocampal segmentation (using FSL's FIRST) and to generate a quality assurance image using FSL utilities (overlay and slices). Note that some targets (such as skstrip and qa) do not correspond to actual files, and are denoted in the first line of Example 2 as “phony” targets. These targets will always be created if their dependencies do not exist. We also notice that the target clean is phony but has no dependencies. It will always be created, allowing us a simple way to remove our work by typing make clean. We can specify multi-line recipes, written in the shell of our choice (here, bash) or simply call programs from other neuroimaging packages. As in the recipe for hippo.csv in Example 2 and in other examples, we make heavy use of bash shell programming and utilities such as awk and sed to manipulate the output of commands. However, recipes can also be executable scripts in other languages.

**Example 2:**
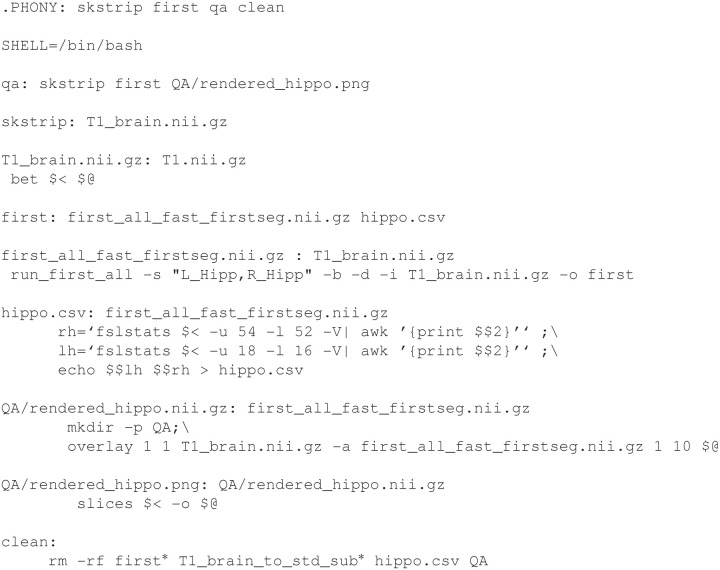
**A more complicated Makefile for hippocampal segmentation**.

**Figure 2:**
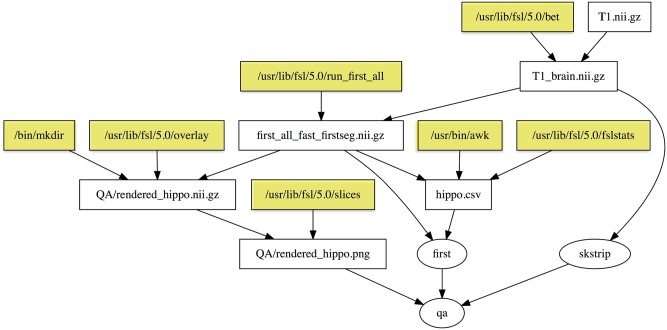
**Dependency graph of the “qa” target defined in the Makefile in Example 2**. Rectangles represent files, and ellipses represent phony targets (that are defined for convenience and do not correspond to actual files). Yellow shaded rectangles are programs installed on the system that are part of the default Linux distribution or FSL utilities, and are important for provenance but not dependency checking.

The dependency graph for the QA target is shown in Figure 2. Note that the dependency graph is created automatically from the specification of the rules, unlike Nipype and LONI Pipeline, which require the user to describe all the connections in a graph. If you add a rule, the dependency graph will be automatically modified. The programmer does not need to specify the dependency graph; it is generated automatically from the individual inputs and outputs of specific steps. Therefore, it is easy to break the workflow into individual rules as shown here for development, testing, and reuse.

Because Make is organized around the concept of a dependency graph, the primary arguments that control its execution are targets to build and variables that parameterize its execution. In this way it is possible to specify that only a specific part of the dependency graph should be executed, or that a specific subject or session should be used.

After nearly 40 years, Make is still widely in use and is the basis of many derivative systems, ranging from simple reimplementations that support new features to new build systems that address limitations of Make. For example, makepp is a drop-in replacement for Make that supports generation of dependency graphs (Pfeiffer and Holt, 2013) as shown in Figure 2. CMake is a popular cross-platform make system that addresses the issue that people may want to build software across systems that run different operating systems and have different commands (Martin and Hoffman, 2013). Snakemake is a Python-compatible workflow system (with a Python-like syntax) based on the logic of Make (Köster and Rahmann, 2012). FreeSurfer supplies a -make option to run via a standard makefile. Any variant of Make can be used as described in this paper to support neuroimaging workflow. However, most Linux platforms come supplied with a version of GNU Make released by the Free Software Foundation, which is stable, robust, and excellently documented. The examples in this paper and in the manual (see Supplemental Materials) all work with GNU Make version 3.8.1. The newest version of GNU Make (version 4.1 as of this writing) supports additional useful features, but it may not be available on all systems.

### Organizing neuroimaging projects to work with make

**Figure 3:**
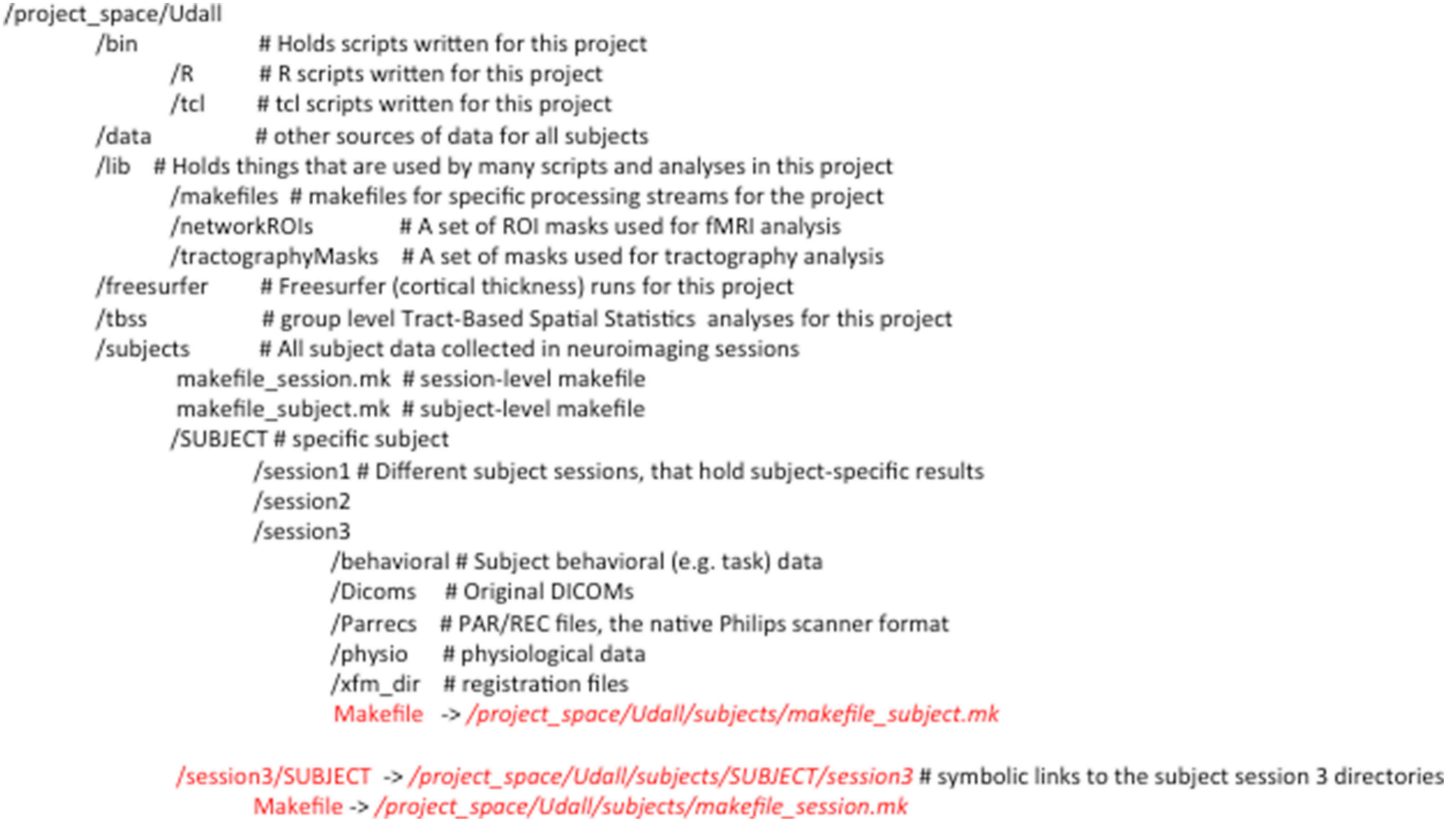
**Example directory structure for use with Make**. Symbolic links are typeset in red with arrows to the files they point to.

The use of consistent file and directory naming conventions is critical for any scripted data analysis, as it is for Make. We typically create a directory for each project that contains (1) subdirectories for scripts and programs specific to the analysis (bin), (2) masks, templates and makefiles (lib), (3) auxiliary behavioral data (data), and (4) one or more sessions of subject imaging data. Figure 3 shows the directory structure for a project called Udall, for which each subject was imaged on three occasions. We use symbolic links (files that reference other files) to create the illusion that the data are organized by subject/timepoint, and by timepoint/subject. This can be seen in Figure 3 by observing that /project_space/Udall/subjects/SUBJECT/session3 is the same directory as /project_space/Udall/subjects/session3/SUBJECT. This organization is convenient for longitudinal analyses. By creating links to subject and session-level makefiles, it is possible to build targets for both a single subject/timepoint, and across all subjects at a single timepoint (by calling Make recursively within each subject directory). This use of Make limits the depth of directory trees that researchers have to keep track of while exposing parallelism of processing multiple subjects (see Supplemental Materials: Setting up an Analysis Directory for more details). However, symbolic links can be confusing for users and system managers and it may not be necessary to expose the single subject/timepoint organization. In this case it is possible to copy subject and session-level makefiles and avoid additional complexity. In practice both approaches work successfully.

### Parallel execution

Most neuroimaging pipelines are inherently parallelizable—even if the processing of a single scan cannot be parallelized, typically the subject-specific processing of most data does not depend upon other subjects. This type of processing is called “embarrassingly parallel” (Moler, 1986; Foster, 1995) because of this lack of dependency, and is well suited to running on multiple cores in a single shared memory multiprocessor, or on clusters of computers that are grouped together with a scheduler such as the Sun Grid Engine (now Open Grid Scheduler). Because a dependency graph expresses inherent parallel structure, a single makefile is a parallel program that can be easily executed on either a multicore machine (using the -j flag to specify the number of cores to use) or on a cluster using qmake, an Open Grid Scheduler-supplied utility. When working with thousands of subjects, after performing QA and adjusting critical steps in the pipelines, redeploying make will automatically parallelize only the remaining work, based on dependency analyses of which targets need to be regenerated.

### Fault tolerance

Fault tolerance is an additional feature Make provides that is not natively supported by scripting languages. Because Make builds and executes recipes based on a dependency graph, when a step in the processing pipeline fails (e.g., due to a cluster node going down during a long running job, a hard drive filling up, a computer running out of memory, or a failure to complete a step correctly due to corrupted inputs) it can be re-executed with the same command line and will recreate only those dependencies that still need to be updated.

As a concrete example, consider the following bash script that runs multiple subject level GLM analyses in parallel using FSL's FEAT program. Parallelism is provided by FSL's fsl_sub program and enabled by setting the FSLPARALLEL environment variable. Because the cluster machines have limited memory, sometimes some arbitrary number of these analyses will fail (Note that by default FEAT will append a + to the output directory if it already exists but we assume the .fsf configuration file specifies overwriting it instead).


  \#!/bin/bash
  export FSLPARALLEL=true
  for subject in ‘cat subjects‘
  do
       if [ -f ${subject}/firstlevel.feat/
                        stats/cope5.nii.gz ]
       then
       feat ${subject}/firstlevel.fsf
       fi
  done


Fault tolerance is implemented here explicitly by checking for the existence of the final cope (number 5), so that the script can be rerun until all jobs have completed. An alternative approach would be to edit the script to include only the subjects who need to be reprocessed. Because coding workflow for fault tolerance is normally an afterthought, the latter approach is frequently used in scripts, leading to errors and inconsistencies in the processing of the data.

In contrast, a makefile to do this task would be written as follows:


  subjects=$(shell cat subjects)
  all: $(subjects:=/firstlevel.feat/stats/
                               cope5.nii.gz)
  
  %/firstlevel.feat/stats/cope5.nii.gz:
                     *<dependencies>*
       feat $^*^/firstlevel.fsf


As above, the subjects are obtained from the file called subjects. The main target all uses the subject names to construct the lists of copes to be created. A single recipe asserts that the cope depends upon some optional set of dependencies (here these are not specified and indicated by a placeholder in italics). Although, missing from the bash script, these dependencies could be used to specify that the FEAT analysis should be rerun if any of the dependencies (e.g., regressors, fMRI input file, or.fsf file) changes. The same makefile can be used to execute in parallel (with any degree of parallelism required to allow sufficient memory for the jobs) or sequentially. Finally, no explicit test is required to rerun the FEAT analysis if the cope does not exist; fault tolerance in Make is implicit.

### Writing self-documenting makefiles

One of the advantages of using makefiles is that phony targets can be defined that allow underlying neuroimaging calls to be conceptually named or grouped. For example, in Example 2, the phony target “skstrip” will run bet to perform skull stripping, and the phony target “transforms” will create all the necessary transformation matrices using flirt, epi_reg, or convert_xfm. Writing makefiles in this way makes it easier for people who are new to neuroimaging to understand and work with the pipelines. Documenting these targets is helpful, and our first approach was to create a target that produces a “usage” message for the makefile. However, we quickly discovered that it is also easy to fail to keep documentation up to date as new targets are added, removed, or modified.

One solution to this problem is to define a macro that can be used to annotate targets of interest with a descriptive help message. In this way, the help message and the target are close together in the makefile, so that it is easier to keep the help up to date. The target depends upon the help message being displayed, if requested. This has been implemented in a help system described by Graham-Cumming (2015), that produces a list of targets and their corresponding line numbers in specific makefiles. For example, processing of a single subject involves several piplines (e.g., for registration, structural analysis, resting state preprocessing, and functional connectivity analysis). Each of these can pipelines is cleanly written in its own makefile. To see this implemented in real example, see Supplemental Materials: Processing Data for a Single Testsubject.

**Example 4** (discussed in detail later) is a makefile to perform registrations. To document the transforms target in **Example 4**, one would modify it as follows:


  transforms: $(call print-help, transforms,
  Create resting state to MNI transformations)
  xfm_dir xfm_dir/MNI_to_rest.mat


We refer the reader to a detailed online description of how the help system is implemented (Graham-Cumming, 2005). However, to summarize, we add a “help” dependency to each target that we wish to document. This takes the form of the call function to Make shown above. When the user types “make help,” all help messages will be printed. If **Example 4** is located in the Makefile xfm.mk, the help message for target transforms (highlighted) would appear along with other target help messages written for other targets (to execute several subject-level processing pipelines) as shown below. This helps people find the targets that are defined by multiple makefiles in separate files.


  resting.mk:20: rest -- "Run resting
  state preprocessing pipeline"
  xfm.mk:3: transforms -- Create resting
  state to MNI transformations
  fcconnectivity.mk:6: Connectivity --
  "Perform subject-level seed-based
  connectivity analysis"
  QA.mk:10:  qa --  Create QA report
  Makefile:42: all -- Do skull stripping 
  etiv & HC volume calculation
  Makefile:55: robex -- Alternate skull
  stripping with ROBEX
  Makefile:61: freesurferskullstrip --
  Alternate skull stripping with FreeSurfer
  Makefile:71: etiv --  Estimation of ICV
  using enigma protocol
  Makefile:100: flex --  Run flex for white
  matter hyperintensity quantification
  Makefile:142: clean -- Clean up everything
  from all makefiles


### Debugging

Debugging makefiles has a reputation for being rather difficult. There are probably several reasons for this. Make has unusual syntax, requiring recipes to be prefaced by TAB characters, and it does not cope well with indiscriminate white space. It has a very different model of execution than scripts (variables are, by default, evaluated at time of use, and targets within a makefile are not executed sequentially). Use of pattern substitution can be difficult. Finally, when executing in parallel, the output messages of multiple jobs are interleaved, making it difficult to interpret these outputs from Make.

On the positive side, makefiles are naturally broken down into targets that can be tested individually. We encourage building of a makefile by adding and testing one target at a time. Once Make frameworks have been developed, most problems lie in debugging the neuroimaging commands. Because recipes are simply shell scripts, it is easy to cut and paste recipes into the command line to test them.

We document common causes of errors or misunderstandings (See Supplemental Materials: Troubleshooting Make). Other strategies that are useful are the use of the “-n” option, which prints out commands without executing them, and the “-p” option, which prints the database of rules (including the result of pattern substitution). The most recent version of GNU Make (4.1) has a -trace option, which describes why given lines are executed. It also has a -output-sync option to synchronize the output of recipes that are executed in parallel on multiple cores, a source of confusion in debugging problems that show up during parallel execution and not sequential execution. These are useful features for debugging, even if this is not the primary version of Make installed on the system.

## Quality assurance and data provenance

Established neuroimaging software packages often include information describing which steps and associated options were applied to the data as well as images and metrics for assessing data quality at various stages (e.g., FSL's FEAT reports). Unfortunately, these systems are necessarily limited to accounting for steps applied within that specific package, limiting their utility when the user incorporates methods from different packages or custom algorithms into a single pipeline. Options are available for wrapper-dependent workflow systems (e.g., MacKenzie-Graham et al., 2008), but are less likely to be routinely employed by users who are executing a range of commands via bash or MATLAB scripts. There are likely multiple ways to address this problem for custom pipelines, but we describe the system we have implemented using a combination of R Markdown (Rstudio, 2015) and Make.

### Quality assurance

The ability to quickly identify problems in data acquisition or processing is important for ensuring reliable results. This is generally accomplished by a combination of viewing selected images (e.g., skullstripping, image segmentation, registration) and examining metrics (e.g., motion parameters, global intensity changes). Tools exist in standard neuroimaging software packages to generate both images (e.g., FSL's slicer) and metrics. To reduce the burden of generating quality assurance (QA) reports (thus increasing the likelihood that QA will be incorporated into workflow), we generate most images and metrics using these existing tools, then aggregate them into reports with R Markdown (Figure 3).

Workflow for QA occurs in two stages. The first is to generate QA metrics and images. For example, in Example 2, FSL's slices is used to create a .png image of the segmented hippocampus overlaid on a limited set of axial, sagittal, and coronal slices of the subject's T1 image. The second stage is to incorporate such images into a single HyperText Markup Language (HTML) report with a script or using R Markdown, as shown in Example 3. Here, QA images for a skull-stripped brain and tissue parcellation are used to generate the report QA/Structural.html. The sed utility is used to replace the generic SUBJECT variable in the R Markdown file with the actual subject identifier so that we know which subject we are looking at. Next, a command to R renders the document. These reports are then checked by research staff.

**Example 3:**
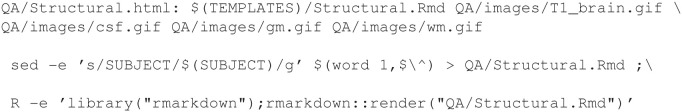
**Creating a QA report**.

R Markdown is a simple authoring format that allows writing text as well as executing chunks of code from a range of languages including R, bash, HTML, and Python. Output is similarly flexible (e.g., .html, .pdf, .doc). For the purposes of QA, we prefer an HTML report over a PDF, because the HTML reports allow embedding of videos (e.g., of timeseries data) and doesn't require the user to control pagination. The flexibility of R Markdown allows generation of QA reports that best suit a given project. One the one hand, it may be desirable to generate a single report showing the data quality for each subject as they are acquired to immediately identify potential problems. On the other hand, for the simultaneous processing of a large, open dataset (e.g., from the Autism Brain Imaging Data Exchange (ABIDE) or Alzheimer's Disease Neuroimaging Initiative (ADNI)), it may be more sensible to create a report that aggregates data from a single modality for many subjects at once. We incorporate R Markdown into Make workflows by specifying the script, images, and metrics as dependencies of a QA report target.

### Data provenance

**Figure 4:**
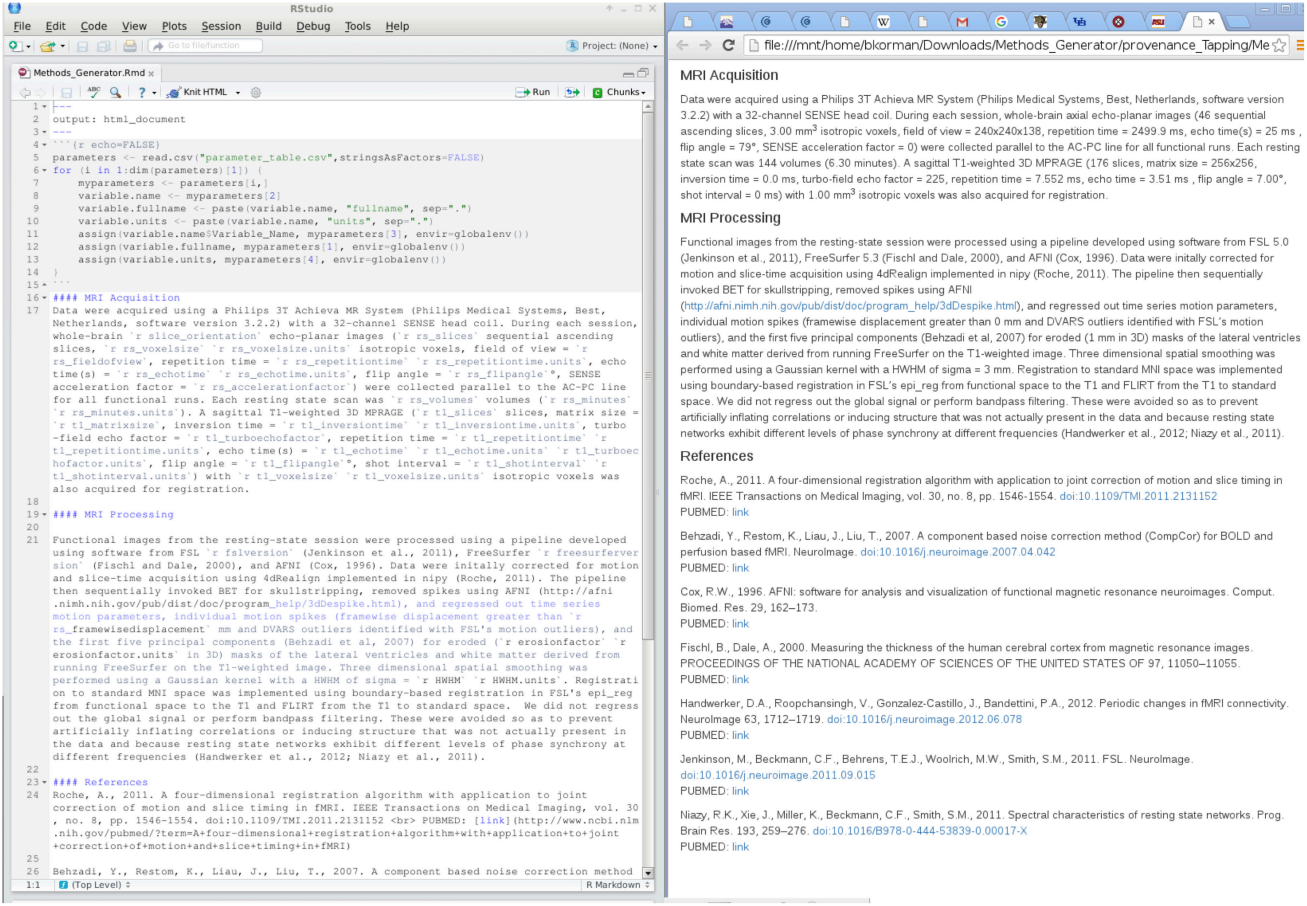
**Data provenance of resting state processing with R markdown**.

Provenance, which is the metadata describing how a dataset was acquired and altered through subsequent manipulation, is critical for ensuring reproducibility and allowing for comparison of study results, combination across datasets, and data sharing. Standards have been proposed for neuroimaging data provenance (Keator et al., 2015) but have not been widely implemented even for tools included in standard software packages. At a minimum, data provenance must be sufficient to allow for replication of data collection, processing, and analysis. Because this standard is the same as the standard for methods reporting in a publication (Poldrack et al., 2008; Carp, 2012), we currently implement a basic form of data provenance as an auto-generated methods section with appropriate references, much in the way that a processing methods description is generated by FSL FEAT for more basic analyses (see Figure 4).

Our methods generator makefile acquires raw MRI acquisition parameters (e.g., repetition time TR, echo time TE) pertinent to reporting by calling a bash script that automatically pulls these parameters from the raw PAR/REC (a Philips-specific file format) data and stores them in a comma separated value (CSV) file. Similarly, the versions of tools (e.g., FSL) as indicated by their installation directories and options for the implemented MRI preprocessing tools (e.g., smoothing kernel size), are pulled from the makefiles for specific processing streams and additionally stored in this CSV file. We can then write an R Markdown methods file that documents the processing streams and is parameterized by the values in this CSV file. The makefile renders this R Markdown file, automatically filling in the parameter values and generating an HTML output report. Although the R Markdown methods file must be changed by hand when pipelines change, minor revisions to software versions will be automatically noted as data are collected for a project. The flexibility of the Make/R Markdown-based system allows for updating the fields included as provenance when mainstream software packages are updated to be compliant with recommended standards.

## Usage examples

Over the last several years we have created pipelines to perform basic subject-level processing of structural, functional, diffusion, and perfusion data using Make, in addition to group level analysis and complex reporting. These incorporate tools from FSL, AFNI, Nipype, FreeSurfer, SPM, ANTs and many other neuroimaging software packages, and custom programs written in shell, MATLAB, Python and R. Examples of these pipelines are documented in the manual (see Supplemental Materials), and accompanying code and examples are available in the “makepipelines” package on NITRC. A sample of these examples is highlighted below.

### Registrations

**Figure 5:**
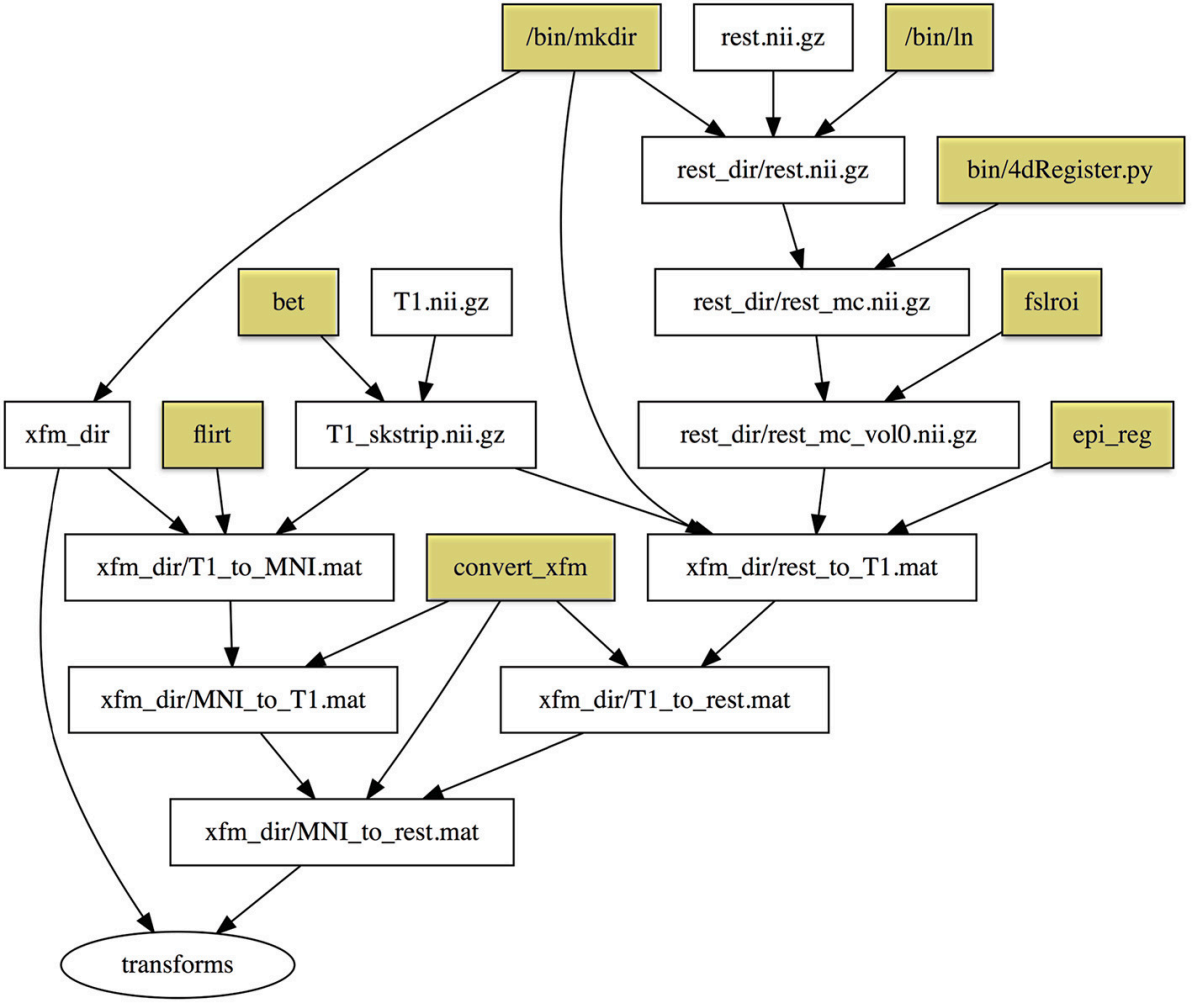
**Dependency graph for Example 5**. Rectangles represent files, and the ellipse represents a phony target (not an actual file). Yellow shaded rectangles are programs installed on the system that are part of the default Linux distribution or FSL utilities, and are important for provenance but not dependency checking. To simplify the graph we do not include full paths to these programs as we did in Figure 2.

Registrations are especially suited for execution with makefiles, because a registration process across several types of images involves multiple steps that can normally be calculated individually (in parallel) and concatenated. It is easy to describe this dependency relationship in Make. The syntax of registration commands and the order of arguments can be difficult to remember, but these are abstracted by targets in the makefile. Interim registrations need to be checked and may be hand-corrected, in which case make will regenerate only what needs to be updated following modification. Example 4 (See Supplemental Materials: Testsubject Transformations for a detailed description) is an example of a makefile to create resting state to standard MNI space transformations. Figure 5 shows the dependency graph generated for this example.

**Example 4:**
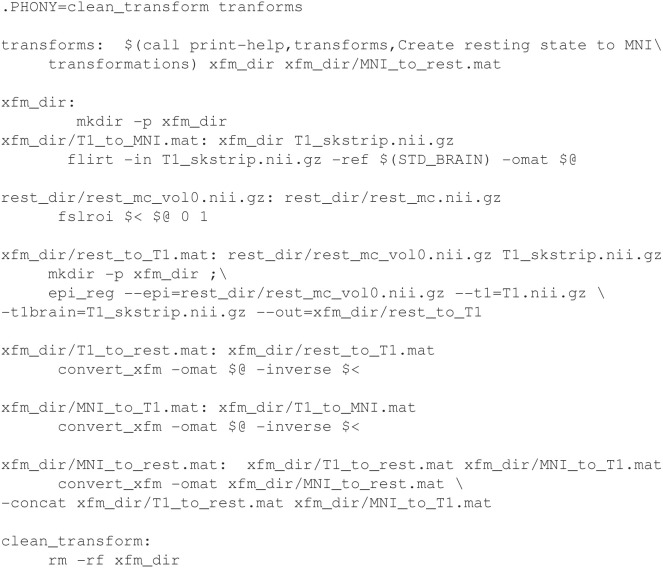
**Registering fMRI data to standard space using FSL utilities**.

### Conditional processing

Often different processing or analysis methods may be desirable based on data type or on the availability of ancillary data (e.g., physiologic data). Make is well-suited to handling workflows which require different actions under different circumstances. Of course, many programming languages allow for conditional logic. However, a conditional in Make can be used to specify different dependencies for a target. When the makefile is read, the conditional will be evaluated and the dependency graph will include only the necessary components.

In Example 5 (explained in greater detail in Supplemental Materials: DTI Distortion Correction with Conditionals), the final target, “tensor” (highlighted in blue), requires creation of a distortion corrected image called “sdc_mec_diffusion.nii.gz.” The recipe required for generating the distortion corrected data is chosen automatically by Make depending on whether a field map image or an acquisition parameters text file is present, as reflected by the variable SDC_METHOD (in red). This variable is queried later in the corresponding conditional set of rules (also highlighted in red). It is worth noting that the evaluation of the conditional takes place before any commands are run, because Make first needs to construct the dependency graph. This means that if files are created or deleted during a single invocation of Make, it may affect the behavior of the commands that are called, but it won't change whether they are executed, or the sequence in which they are executed, unless it causes an exit due to an error.

**Example 5:**
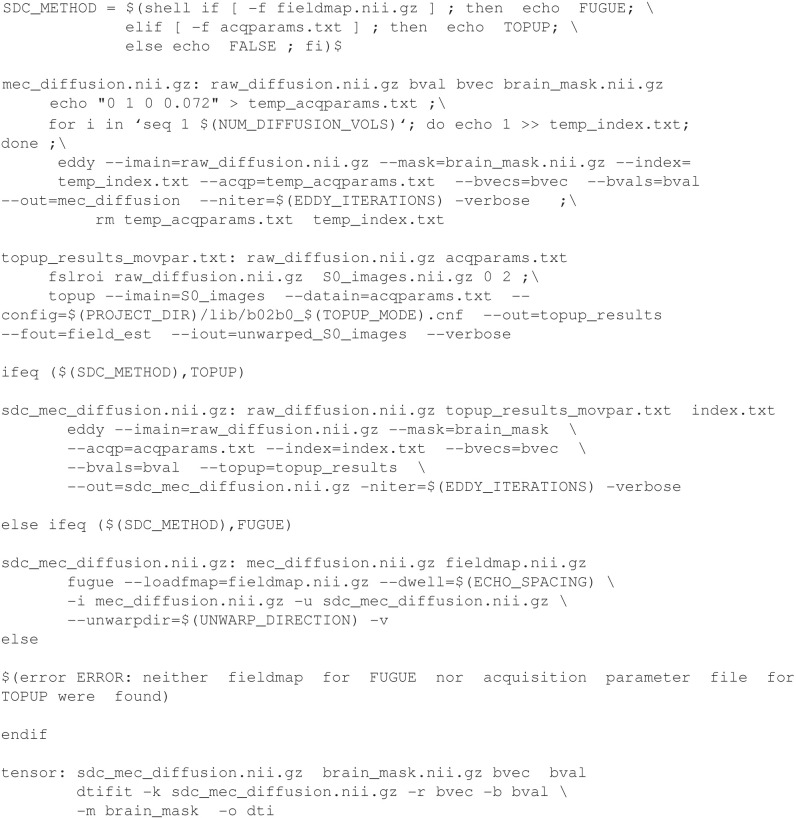
**DTI Distortion Correction with Conditionals**.

### Integration of software packages

One of the strongest motivations to write one's own workflow is to be able to use the best tools available for any task, even if some of those tools are found in other software packages. The challenge is making tools from different packages work together seamlessly when one is necessarily introducing an edge case that the developers of the software packages could not have anticipated. In Example 6 (explained in more detail in Supplemental Materials: Using ANTs Registration with FEAT), a directory created by FSL's feat for a first-level (timeseries) analysis is modified to allow for running a follow-up higher level feat analysis in standard space without relying on FSL tools for registration from functional to standard space. To accomplish this with registrations calculated using the ANTs package, ANTs is used to move the statistic images from the first level feat into standard space, and then output files are created using the naming conventions of the FSL package. This allows later FSL processing to continue without error. An interesting feature of this example is that it includes a Make function written to identify all of the available statistic (cope, varcope) images without having to specify them explicitly. The variable number of statistics images are identified and rules to Make are created when Make is run. With this method, Make can be allowed to parallelize registration of the statistic images which do not depend on each other.

**Example 6:**
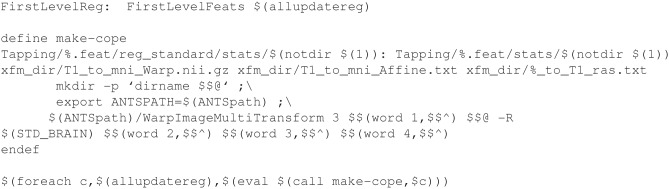
**Registration of feat outputs with ANTs**.

## Discussion

None of the individual tools described in this paper are novel. The novelty is that we demonstrate how they may be used in combination to quickly implement sophisticated and dynamic neuroimaging workflow, with a supporting manual, lab practicals, and data and examples downloadable from NITRC. The simplicity of using a build system to track workflow has been described for spike train analysis (Denker et al., 2010) and anecdotally noted in several blog postings as a feature for reproducible science (Butler, 2012; Hyndman, 2012; Bostock, 2013; Hambley, 2013; Jones, 2013). This has driven development of several Make-like systems geared toward specific types of data analysis (e.g., Drake, Factual, 2015, Nextflow, Tommaso, 2015). Instead of designing a new neuroimaging workflow system we have chosen to illustrate what can be done with existing technologies.

It should be noted that although using Make to track workflow helps scientists to be able to reproduce their findings in the sense of obtaining the same results using the same data and software, nothing about Make will help to improve reproducibility in the sense of conducting new experiments on different data (Collaboration, 2015). However, because workflows can be coded using Make and shared across UNIX systems with minimal software installation effort, like scripts, they can contribute to sharing and replicating identical processing steps on different data sets.

SPM and FSL provide graphical user interfaces for codified analyses that offer limited flexibility. Within FSL, tools like FEAT allow the user to select from pre-specified processing and analyses steps to be applied in a specific recommended order, and basic shell scripts can be used to modify output from these GUIs to generalize across subjects. Similarly, within the SPM GUI, individual steps can be selected and combined within the batch system to create simple MATLAB scripts. These systems are useful for the introductory user; however, they lose some of their appeal when the scientist wants to combine tools from multiple packages or introduce custom packages in the middle of the pre-specified workflow.

We argue that there is a lack of workflow options that exist just beyond scripting, for people who want to do a more conceptually sophisticated analysis, or even a simple analysis on a very large scale. With the availability of large open datasets, there is a growing market for both types of people. When workflows must be frequently modified and are expected to scale, minimizing the complexity of code that must be understood and edited to make these changes is important. This is the use case addressed by this paper. Our target audience is people who can become familiar with basic coding principles, but who either lack the skills or the interest for extensive software development.

One of the advantages of Make as a workflow solution for this audience is that it does not wrap or abstract the details of neuroimaging command arguments, except by defining phony targets. When standards are established, implementing layers of abstraction (while preserving performance) improves ease of use, in terms of skill required and time to implement, debug, and maintain workflows. Wrappers can also be used to add additional checks for correctness of inputs and calling conventions. However, in a dynamic neuroimaging environment, every time a new version of a wrapped package is released there is the potential for many things to break, and individual users must wait for the person maintaining the wrappers to fix them. Including a new package means having to wrap it (and maintain it) to interoperate with everything else. Therefore, the decision of how much abstraction a workflow system should provide should ultimately be based upon a cost-benefit analysis of the cost of the additional time and software expertise needed to maintain it (dependent upon the frequency of changes to pipelines and the underlying abstracted software layers) compared to the benefits of providing such abstractions (in terms of ease of use and improved functionality).

Make is not without limitations. It is necessary to be willing to code to use it; it does not have a graphical user interface for designing dependency graphs (such as LONI pipeline). However, complicated syntax is not required for many examples that afford the parallelization and fault tolerance not provided by a shell script, and more sophisticated features can be mastered by copying existing examples. It relies upon file timestamps, so it cannot transparently operate on web resources (e.g., images stored in XNAT databases) without first downloading the files. To handle large analysis directories it is necessary to select specific output files that represent late-stage products to “stand-in” for the analysis. Reliance upon the file system is a potential performance problem for neuroimaging applications as parallelism scales and a common file system becomes a bottleneck. However, some file output is necessary for fault tolerance. We envision that some optimization of existing neuroimaging applications will ultimately be necessary to use I/O more intelligently in a parallel environment. Because none of the neuroimaging applications are wrapped, provenance cannot be tracked by the wrappers themselves and must be handled elsewhere. We have described the use of R Markdown to generate provenance reports. Although cluster parallelism is seamless with qmake and SGE, a parallel scheduler-enabled version of Make does not exist for all cluster environments. We rely upon recursive Make to descend into subject directories and call itself to conduct subject-level processing. Much has been written on the pitfalls of recursive Make (Miller, 1997); however, the problems with descending into multiple build directories stem primarily from an inability to control dependencies between directories. We assume each subject is individual and fully exchangeable with others; therefore these criticisms do not apply to our use of Make.

GNU Make is open source and these features could be modified, or potentially addressed by use of one of the many other featureful build systems based on Make. In fact, in 1979 Feldman noted that more complex file comparisons than simple timestamps could be implemented to expand functionality. However, to make such modifications we would begin to sacrifice the stability and reliability of GNU Make. Having a stable workflow system is a necessity when neuroimaging applications and practices are constantly being changed as the science progresses. We suggest that Make represents a large step toward scientific goals of reproducibility, parallel execution and fault tolerance, with only a modest increase in programming demands over shell scripts.

## Author contributions

MA, TM, ZM, MR: Conception and development of technology. TMD, NK: Substantial editing of supplemental manual. All: Conception and design, drafting and revising, individual examples, and final approval.

### Conflict of interest statement

The authors declare that the research was conducted in the absence of any commercial or financial relationships that could be construed as a potential conflict of interest.

## Footnotes

^1^We used salary for skills from the 2015 Dice Tech Salary report (Dice.com., 2015) as a rough proxy for ability to approximately rank different programming languages (Hunt and Madhyastha, 2012). Bash is an average of bash, shell, and Linux.
