# Supplementary material for: Using Make for Reproducible and Parallel Neuroimaging Workflow and Quality-Assurance
Source: Front Neuroinform. 2016 Feb 2;10:2. doi: 10.3389/fninf.2016.00002 (PMC4735413; doi:10.3389/fninf.2016.00002)
Supplement: Supplementary file 1 [file SupplementaryMaterials.pdf]

IBIC

INTEGRATED BRAIN IMAGING CENTER

Using GNU MAKE for Neuroimaging Workflow

University of Washington

Using GNU `make` for Neuroimaging Workflow

Mary K. Askren*, Trevor K. McAllister-Day, Natalie Koh, Zoé Mestre, Jennifer N. Dines, Benjamin A. Korman, Susan J. Melhorn, Daniel J. Peterson, Matthew Peverill, Swati D. Rane, Melissa A. Reilly, Maya A. Reiter, Kelly A. Sambrook, Karl A. Woelfer, Xiaoyan Qin, Thomas J. Grabowski, and Tara M. Madhyastha†

University of Washington, Seattle

December 18, 2015

*askren@uw.edu

†madhyt@uw.edu

Contents

Contents ii

List of Figures vi

I Manual 1

1 Introduction to `make` 2
- Conventions Used in This Manual . . . 3
- Quick Start Example . . . 3
- A More Realistic Example . . . 5
- What is the Difference Between a Makefile and a Shell Script? . . . 7
- Additional Resources For Learning About `make` . . . 8

2 Running `make` in Context 9
- File Naming Conventions . . . 9
  - Subject Identifiers . . . 9
  - Filenames . . . 11
- Directory Structure . . . 12
- Setting up an Analysis Directory . . . 14
  - Defining the basic directory structure . . . 14
  - Creating the session-level makefiles . . . 15
  - Creating the common subject-level makefile for each session . . . 16
  - Creating links to the session-level makefile . . . 17
  - Creating links to subject-level makefile . . . 17
  - Running analyses . . . 17
- Setting Important Variables . . . 18
  - Variables that control `make`'s Behavior . . . 18
  - Other important variables . . . 19
  - Variable overrides . . . 20
  - Suggested targets . . . 21

3 Running make in Parallel **23**
Guidelines for Writing Parallelizable Makefiles 23
Each line in a recipe is, by default, executed in its own shell 23
Filenames must be unique 24
Separate time-consuming tasks 25
Executing in Parallel 26
Using multiple cores 26
Using the grid engine 26
Setting `FSLPARALLEL` for FSL jobs 27
Using `qmake` 27
How long will everything take? 28
Consideration of memory 28
Troubleshooting `make` 29
Find out what `make` is trying to do 29
Use the trace option in `make` 4.1 29
Check for line continuation 30
No rule to make target! 30
Suspicious continuation in line # 31
`make` keeps rebuilding targets 31
`make` doesn't rebuild things that it should 31

4 Recipes **32**
Obtaining a List of Subjects 32
Setting a variable that contains the subject id 33
Using Conditional Statements 33
Setting a conditional flag 33
Using a conditional flag 34
Conditional execution based on the environment 34
Conditional execution based on the Linux version 35

II Practicals **37**

1 Overview of make **39**
Lecture: The Conceptual Example of Making Breakfast 39
Practical Example: Skull-stripping 41
Manipulating a single subject 41
Pattern rules and multiple subjects 43
Phony targets 44
Secondary targets 45
`make clean` 46

2 make in Context **48**
Lecture: Organizing Subject Directories 48
Practical Example: A More Realistic Longitudinal Directory Structure . . 49
Organizing Longitudinal Data 49
Recursive `make` 51
Running `make` over multiple subject directories 52
Running FreeSurfer 53

3 Getting Down and Dirty with make **54**
Practical Example: Running QMON 54
Practical Example: A Test Subject 55
Estimated total intracranial volume 57
Hippocampal volumes 59
Implementing a Resting State Pipeline: Converting a Shell Script into a Makefile 60

4 Advanced Topics & Quality Assurance with R Markdown **64**
Creating a `make` help system 64
Makefile Directory Structure 65
The `clean` target 66
Creating New Makefile Rules On The Fly 67
Incorporating R Markdown into `make` 67

III Examples **73**

1 Downloading Data From XNAT **75**

2 Running FreeSurfer **78**

3 DTI Distortion Correction with Conditionals **81**

4 Quantifying Arterial Spin Labeling Data **86**

5 Processing Scan Data for a Single Test Subject **91**
Testsubject Main Makefile 91
Testsubject FreeSurfer 95
Testsubject Transformations 97
Testsubject QA Makefile 99

6 Preprocessing Resting State Data **104**

7 Generating A Methods Section **110**

8 Seed-based Functional Connectivity Analysis I **113**

9 Seed-based Functional Connectivity Analysis II **115**
Group Level Makefile - `tapping/Makefile` 115
Subject Level Makefile - `tapping/lib/resting/subject.mk` 116
Preprocessing - `tapping/lib/resting/Preprocess.mk` 117
Generation of Nuisance Regressors - `tapping/lib/resting/Regressors.mk` 119
ROI Timeseries Extraction - `lib/resting/timeseries.mk` 121
First Level Analysis - `lib/resting/fsl.mk` 122
Quality Assurance Reports - `lib/resting/qa.mk` 122

10 Using ANTs Registration with FEAT **124**
Group Level Makefile 124
Subject Level Makefile 125
Preparatory Registrations - `Prep.mk` 126
Running Feat and Applying ANTs Registrations - `Feat.mk` 127

11 Creating Result Tables Automatically Using Make **131**
Simple Result Tables 131
Multiple Group Analyses 132

12 Plotting Group FEAT Results Against Behavioral Measures **134**

Bibliography **142**

List of Figures

1.1 Basic `make` syntax. 4
1.2 A very basic `make` recipe. 4
1.3 Automatic `make` variables 5
1.4 A more realistic example 5
1.5 An expansion of Figure 1.4 6
1.6 A makefile expressed in `bash` 7

2.1 Example of good file naming conventions 11
2.2 An example of a project directory. 13
2.3 A longitudinal analysis directory. 15
2.4 Session-level Makefile 16
2.5 Subject-level makefile 16
2.6 Creating symbolic links to the session-level makefile 17
2.7 Making `test` 17
2.8 Output of recursive make 18
2.9 Setting `$(SHELL)` 18
2.10 Running DTIPrep in make 20
2.11 Controlling software version 20
2.12 Specifying BET flags in make 21

3.1 A non-functional multi-line recipe 24
3.2 A now-functional multi-line recipe 24
3.3 A multi-line recipe using "`&&\`" 24
3.4 How not to name files 24
3.5 The wrong way to run two long tasks 25
3.6 The right way to run two long tasks 25
3.7 Pattern-matching error handling 31

4.1 Obtaining a list of subjects from a file. 32
4.2 Obtaining a list of subjects using a wildcard. 33

4.3 Determining the subject name from the current working directory and a pattern. . . . 33
4.4 Setting a variable to determine whether a FLAIR image has been acquired. 34
4.5 Setting a variable to indicate the study site. . . . 34
4.6 Testing the site variable to determine the name of the T1 image. . . . 35
4.7 Testing to see whether an environment variable is undefined 35
4.8 `/etc/os-release` 36
4.9 Testing the Linux version to determine whether to proceed 36

1.1 Creating a waffwich 40
1.2 How to make a waffwich 41
1.3 Pattern-matching as a sieve 44

2.1 Script to create symbolic links within a longitudinal directory structure 50

3.1 `resting-script` file 62

4.1 `make`'s Help System 65
4.2 Example of a R Markdown File 70
4.3 QA report in R Markdown 72

1.1 XNAT access directory structure. 76

3.1 Flowchart of the Toy Makefile 83

4.1 Installing MATLAB Runtime Libraries and surround subtraction application 86
4.2 Entering location of installation folder for Surround Subtraction standalone MATLAB application. 87

7.1 Methods generation in R Markdown 112

12.1 Report plotting group FEAT results against behavioral measures. 141

Part I

Manual

Chapter 1

A Conceptual Introduction to `make` for Neuroimaging Workflow

This guide is intended for scientists working with neuroimaging data (especially a lot of it) who would like to spend less time on workflow and more time on science. The principles of automation and parallelization, taken from computer science, can easily be applied to neuroimaging to make certain parts of the analysis go quickly so that the computer can do what it's best at. This kind of automation supports reproducible science (or at the very least, allows you to rerun your analysis extremely quickly with a variant of the processing stream, or on a new dataset).

Over the last few years we have developed neuroimaging workflows using `make`, a program from the 1970s that was originally used to describe how to compile and link complicated software packages. It is an amazing program that is still in use today. It is fairly easily repurposed to describe neuroimaging workflows, where you specify how you "make" one file, which can depend on several other files, using some set of commands. Once you have expressed these "rules" or "recipes" in a file (a Makefile), you actually have a fully parallelizable program, which allows you to run the Makefile over as many cores as you have (or on a Sun Grid Engine). This has been done before: FreeSurfer incorporates `make` into their pipeline "behind the scenes." We have developed many examples of neuroimaging pipelines in multiple domains that are written using `make`.

Modern tools for neuroimaging workflow (nipype, LONIPipeline) incorporate data provenance (e.g., information about how and when the results were generated), wrappers for arguments so that you do not need to worry about all the different calling conventions of programs, and generation of flow graphs. However, `make` allows expression of neuroimaging workflow with only the programming concepts of parallelism and variables. Being able to incorporate programs from many different packages without wrapping them saves time and effort. T. M. M. has taught several

undergraduates, graduates, and staff to use it to develop, debug, and execute pipelines. These students have contributed examples to this manual. It is simple enough that the basic concepts can be understood by non-programmers. This results in more time teaching and doing neuroimaging than teaching (and doing) programming.

Conventions Used in This Manual

By convention, actual commands or filenames will be typeset in fixed-width (monospaced) font (for example, `ls`, `make`, FSL's `bet`, and `flirt`.) Makefile examples will also be typeset in monospaced font.

Commands to be executed in a shell (we assume `bash` throughout this manual) are written on a line beginning with a dollar sign ($) and displayed between two horizontal lines, as follows:

```
$ echo Hello World!
```

! You do not type the $ to execute `bash` commands; it stands for the prompt.

Examples of commands from Makefiles are shown in an outlined box with no special prompt character:

```
This is a make command.
```

Additionally, recipes are easily identified by their opening syntax, where the recipe target is usually rendered in blue (see Figure 1.1 or Figure 1.2).

Data for the lab practicals and examples documented in this manual are available from NITRC. We will assume that you have downloaded them into some location on your own machines (e.g. `makepipelines`) and will refer to these files directly. To run the example makefiles, you must set an environment variable, `MAKEPIPELINES` to the location of this directory (see Examples). If you are at IBIC you may find these examples on `/project_space/makepipelines`.

Throughout this manual we assume a UNIX style environment (e.g., Linux or MacOS) with common neuroimaging packages installed. All examples have been tested on Debian Wheezy using GNU Make 3.81. Please let us know of problems or typos, as this manual is a work in progress.

Quick Start Example

A makefile is a text file created in your favorite text editor (e.g., `txtedit`, `emacs`, `vi`, `gedit` – *not* Office or OpenOffice). By convention, it is typically called "Makefile" or "foo.mk" (i.e., it has a .mk extension). It is like a little program that is run by the

! Recipes in `make` need to begin with a TAB character. Not eight spaces, not nine, but exactly one TAB character.

program `make`, in the same way that a shell script is program run by the program `/bin/bash`.

A makefile contains commands that describe how to create files from other files (for example, a skull-stripped image is created from the raw T1 image, or a T1 to standard space registration matrix is created from a standard space skull-stripped brain and a T1 skull-stripped brain).

These commands take the form of "rules" that look like those in Figure 1.1.

```
target: dependencies

        Shell commands to create target from dependencies (a
        recipe), beginning with a [TAB] character.
```

Figure 1.1: Basic `make` syntax.

This may be best understood by example. Figure 1.2 is a simple makefile that executes FSL's `bet` command to skull-strip a T1 NIfTI file.

```
s001_T1_skstrip.nii.gz: s001_T1.nii.gz
        bet s001_T1.nii.gz s001_T1_skstrip.nii.gz -R
```

Figure 1.2: A very basic `make` recipe.

In Figure 1.2 the target file is `s001_T1_skstrip.nii.gz`, which "depends" on `s001_T1.nii.gz`. More specifically, to "depend on" something means that *a)* it cannot be created unless the dependency exits, and *b)* it must be recreated if the dependency exists, but is newer than the target.

The "recipe" is the `bet` command that creates the skull-stripped image from the T1 image. This executes in a shell, just as if you were typing it in the terminal.

If this rule is saved inside a file called `Makefile` in the same directory as `s001_-T1.nii.gz`, then you can create the target as follows:

```
$ make s001_T1_skstrip.nii.gz
```

Alternatively, in this instance, you could call:

```
$ make
```

If you specify a target as an argument to make, it will create that target. If you do not, `make` will build the first target in the file. In this case, they are the same.

A More Realistic Example

In the example above, we specified exactly what file to create and what file it depended upon. However, in neuroimaging analyses we normally have many subjects and want to do the same things for all of them. It would be a royal pain to type in every rule to create every file explicitly, although it would work. Instead we can use the concepts of variables and patterns to help us. A variable is a name that is used to store other things. You can set a variable to something and then refer to it later. Figure Figure 1.3 lists the syntax for common `make` variables. A pattern is a substring of text that can appear in different names, and is denoted using `%`. Suppose we have a directory that contains the T1 images from 100 subjects with identifiers s001 through s100. The T1 images are named `s001_T1.nii.gz`, `s002_T1.nii.gz` and so on. This makefile will allow you to skull strip all of them (and on as many processors as you can get a hold of (see Running `make` in Parallel) but I'm getting ahead of myself here).

`$@`	is the target.
`$<`	is the first dependency.
`$(VAR)`	is a `make` variable.

Figure 1.3: Automatic `make` variables

The first two lines in Figure 1.4 set variables for Make. Yes, the syntax is icky but it is well explained in the GNU `make` manual. We will summarize here. The first variable is assigned to the result of a "wildcard" operation that expands to all files with the pattern `s???_T1.nii.gz`. If you are not familiar with wildcards, if you do a directory listing of that same pattern, it will match all files that begin with an "s," are followed by exactly three characters, and then "_T1.nii.gz." In other words, the `T1files` variable is set to be all T1 files belonging to those subjects in the current directory.

```
T1files=$(wildcard s???_T1.nii.gz)
T1skullstrip=$(T1files:%_T1.nii.gz=%_T1_skstrip.nii.gz)
all: $(T1skullstrip)
%_T1_skstrip.nii.gz: %_T1.nii.gz
	bet $< $@ -R
```

Figure 1.4: A more realistic example

The second variable is set using pattern substitution on the list of T1 files, substituting the file ending (`_T1.nii.gz`) for a new file ending (`_T1_skstrip.nii.gz`)

to create the list of target files. Note that the percent sign (%) matches the subject identifier. It is necessary to use the percent sign to match only the subject identifier (and not the subject identifier plus the following underscore, or some other extension) when matching parts of file names in this way.

We have now introduced a new type of rule (`make all`) which has no recipe. Make will look for a file called `all` and this file will not exist. It will then try to create all the things that `all` depends on (and those files, the skull stripped images, don't exist either). So it will then take names of each skull stripped file, one by one, and look for a rule to make it. When it has done that, it will execute the (nonexistent) recipe to make target `all` and be finished. Because the file `all` still does not exist (by intention), trying to make the target will always result in trying to make all the skull stripped files.

This brings us to the final rule. The percent sign in the target matches the same text in the dependency. This target matches the name of each of the skull stripped files desired by target all. So one by one, they will be created. In the recipe for the final rule, we have used a shorthand of `make` to refer to both the dependency that triggered the rule (`$<`) and the target (`$@`). We do this because since the rule is generic, we do not know exactly what target we are making. However, we could also write out the variables (as seen in Figure 1.5).

```
%_T1_skstrip.nii.gz: %_T1.nii.gz
	bet $*_T1.nii.gz $*_T1_skstrip.nii.gz -R
```

Figure 1.5: An expansion of Figure 1.4

In this version of the rule, we use the notation `$*` to refer to what was matched in the target/dependency by `%`. If the previous version looked to you like a sneeze, this may be more readable.

Now you can run this makefile in many ways. As before, to create a specific file, you can type:

```
$ make s001_T1_skstrip.nii.gz
```

To do everything, you can type: `make all` or just `make`.

Suppose you do this and later find that the T1 for subject 036 was not properly cropped and reoriented. You regenerate this T1 image from the dicoms and put it in this directory. Now if you type make again, it will regenerate the file `s036_T1_-skstrip.nii.gz`, because the skull strip is now older than the original T1 image. Suppose you acquire four more subjects. If you dump their T1 images into this directory following the same naming convention and type `make`, off you go.

This probably seems like an enormous amount of effort to go to for some skull stripping. However, the benefit becomes clearer as the complexity of the pipelines increases.

What is the Difference Between a Makefile and a Shell Script?

This is a really good question. A makefile is basically a specialized program that is good at expressing how X depends upon Y and how to create it. The recipes in a makefile are indeed little shell scripts, adding to the confusion. By making these dependencies explicit, you enable the computer to execute as many of the recipes as it can at once, finishing the work as quickly as possible. It allows the computer to pick right back up if there is a crash or error that causes the job to die somewhere in the middle. It allows you, the scientist, to decide that you want to change some step three-quarters of the way down and redo everything that depends upon that step. However, `make` will do no more work than it has to. It will not rebuild anything that does not depend on anything you have changed, however indirectly. And magically, once you have made dependencies explicit, you never need to remember what needs to be updated following a change. The computer will do it for you.

Shell scripts are more general programs that can do all sorts of things, but inherently do them one at a time. For example, the shell script that is the equivalent to Figure 1.4 could be written something like Figure 1.6.

```
#!/bin/bash
for i in s???_T1.nii.gz
do
name=`basename $i .nii.gz`
bet $i $name_skstrip.nii.gz -R
done
```

Figure 1.6: A makefile expressed in `bash`

There is nothing in this script to explicitly tell the computer that each individual T1 image can be processed independently of the others; the order does not matter. So if you are on a multicore computer that can execute many processes at once, you could not exploit this parallelism with this shell script. Furthermore, you can see that to add a few subjects or redo a few subjects, you will need to edit the script to avoid rerunning everyone.

If you have ever found yourself writing a shell script to do a large batch job, and then commenting out some of the subject identifiers to redo the few that need redoing, then commenting out some other parts and adding new lines to the program, and so forth, you are dealing with the problems that `make` can help with. If you are not convinced, see Overview of `make` for a more detailed explanation of how `make` works.

Additional Resources For Learning About `make`

The focus of this manual is on structuring neuroimaging projects using `make`. These additional books and manuals will be extremely helpful for learning about `make` more generally.

GNU Make Manual, Free Software Foundation. Last updated Oct 5, 2014. Link to manual. Although this manual is for version 4.1, which is a somewhat newer version than we use in our examples, most of the information here is the same. This is an excellent reference for the syntax of `make` and its functionality.

Managing Projects with GNU Make, Third Edition by Robert Mecklenburg. Nov 2004. Link to open book content. This O'Reilly book covers a lot of basics but in a readable form for someone trying to manage a large scale project. Information about directory structures is probably less relevant for neuroimaging applications.

The GNU Make Book by John Graham-Cumming. April 2015. Link to purchase. This book has an enormous amount of useful advanced information, including details describing differences between different versions of `make`, many approaches to debugging, and the help system that we use in our examples.

Chapter 2

Running make in Context

In the previous chapter we presented some toy examples. However, in real neuroimaging life one deals with hundreds of subjects with multiple types of scans and many planned analyses. To keep this straight requires some conventions, whether you use `make` or not. Here we describe some conventions that are useful for managing realistic projects.

File Naming Conventions

File naming conventions are critical for scripting in general, and for Makefiles in particular. `make` is specifically designed to turn files with one extension (the extension is the last several characters of the filename) into files with another extension. For example, in Figure 1.2 we turned a file with an extension "T1.nii.gz" into a file with the extension "T1_skstrip.nii.gz."

! **"Extension"** often refers to the file suffix, e.g., ".csv," but there is no reason it must be limited to a fixed number of characters after a period.

So we can use naming conventions within a project, and across projects consistently to reuse rules that we write for common operations. In fact, `make` has many built-in rules for compiling programs that are absolutely useless to us. But we can write our own rules.

Thus, it is important to decide upon naming conventions.

Subject Identifiers

The first element of naming conventions is the subject identifier. This is usually the first part of any file name that we might end up using outside of its directory. For example, we might include the subject identifier in the final preprocessed resting state data, allowing us to dump all those files into a new directory for subsequent analysis with another program, without having to rename them or risk overwriting

other subject files. Some features (by no means exhaustive) that may be useful for subject identifiers are:

1. **They should contain some indicator of which project the subjects belong to.**
   This is particularly important if you work on multiple projects, or if you may be pooling data from multiple studies. Typically choose a multi-letter code that is the prefix to the subject ID.
2. **If applicable, they should contain some indicator of treatment group that the subject belongs to.**
   It is helpful to indicate in the subject ID (usually with another letter or digit code) whether the subject is part of a treatment group or a control.
3. **They should be short.**
   Short names are easier to type and to remember, and make it easier to work with lists of directories that are named according to the subject id.
4. **They should be the same length.**
   This is not necessary but helpful for pattern matching using wildcards in a variety of contexts within `make` and without.
5. **They should sort reasonably in UNIX.**
   Many utilities ultimately require a list of filenames, which is easy to generate using wildcards and the treatment group code if the subject ID sort order is reasonable. The classic problem is to name subjects S1 . . . S10, S11 – the subjects will not be in numeric order without zero-padding to the subject numbers.
6. **They should be easy to type.**
   `bash` has many tricks to help you avoid typing, but when you have to type, the farther you have to move the longer it takes. Do you really need capital letters?
7. **They should be easy to remember for short periods of time.**
   My attention span is very short when doing repetitive tasks; subject IDs that are nine-digit random numbers are harder to remember than subject IDs that are four letter random sequences.
8. **They should be easy to divide into groups using patterns.**
   My favorite set of subject IDs were randomly generated four letter sequences that sounded like fake English words, making it easy to divide the subjects into groups of just about any size using the first letters and wildcards. This makes it very easy to test something on a subset of subjects.
9. **They should be consistently used in all data sets within the project.**
   If the subject identifier is "RC4101" in a directory, it should not be "RC4-101"

in the corresponding REDCap database[1] and "101" in the neuropsychological data. It is easier if everyone can decide upon one form of name.

Filenames

File naming conventions begin with converted raw data (NIfTI files derived from the original DICOMs, for example), and continue on for each level of processing. We find it helpful to give these files specific names and then to perform subject-specific processing within the subject/session directory. The filenames for a recent active project are shown in Figure 2.1, where the subject ID is "RTI001."

Filename	Description
`RTI001_T1_brain.nii.gz`	Skull stripped T1 MPRAGE image
`RTI001_T1.nii.gz`	T1 MPRAGE image
`RTI001_read.nii.gz`	Reading task scan
`RTI001_read_rest.nii.gz`	Resting state scan for reading session
`RTI001_read_fMRIB0_phase.nii.gz`	B_0 Field map phase image for reading session
`RTI001_read_fMRIB0_mag.nii.gz`	B_0 Field map magnitude image for reading session
`RTI001_read_fMRIB0_mag_brain.nii.gz`	Skull stripped B_0 magnitude image
`RTI001_write.nii.gz`	Writing task scan
`RTI001_write_rest.nii.gz`	Resting state scan for writing session
`RTI001_write_fMRIB0_phase.nii.gz`	B_0 Field map phase image for writing session
`RTI001_write_fMRIB0_mag.nii.gz`	B_0 Field map magnitude image for writing session
`RTI001_write_fMRIB0_mag_brain.nii.gz`	Skull stripped B_0 magnitude image
`RTI001_DTI.nii.gz`	DTI image
`bvecs.txt`	b-vectors for DTI processing
`bvals.txt`	b-values for DTI processing

Figure 2.1: Example of good file naming conventions

[1] REDCap is the Research Electronic Data Capture web application that we use for storing subject-specific information.

The specific file names chosen are not as important as consistency in the use of extensions and names, and documentation of what these files are and how they were processed. The more you can keep things the same between projects, the more Makefiles you will be able to reuse from one study to another with minimal changes.

Directory Structure

Typically, we create a directory for each project. Within that project directory is storage for scripts, masks, data files from other sources (e.g., cognitive or neuropsychological data) and subject neuroimaging data. Subjects may have different timepoints or occasions of imaging data, as well as physiological data and task-related behavioral data. An example hierarchy (for project Udall) is shown in Figure 2.2. We have recreated this directory structure in `$MAKEPIPELINES/Udall`, without any actual MRI data, so that you can follow the structure. You can also look at chapter 2 for a description of how to set up a similar directory structure for an actual data set.

This directory is typically protected so that only members of the project who are permitted by the Institutional Review Board to access the data may `cd` into the directory, using a specific group related to the project. The setgid bit is set on the project directory and the file permissions are set by default to permit group read, write, and execute permission for each individual so that files people create within the directory substructure are accessible by others in the project.

? The `setgid` bit on a directory ensures that files created here assume the group to which the directory belongs, and not the group to which the person creating them belongs.

The structure is set up for a longitudinal project, where each subject is imaged at separate time points (sessions). All subject data is stored under the directory subjects (under subdirectories corresponding to each session). However, because often it is convenient to conduct analyses on a single time point, we create a convenience directory at the top level (e.g., session3) which has symbolic links to all of the subjects' session 3 data. This can make it easier to perform subject-specific processing.

Notice that in the project directory, we keep scripts that we write to process the data in the `bin` directory. Although `bin` historically is where "binary" executable files are kept, the distinction between executable `bash` scripts and compiled executables is not terribly important. In this example we separate `tcl` scripts and `R` scripts to make things neater.

Other data files and miscellaneous support files for workflow are stored in the `lib/` subdirectory. Again, your naming conventions may differ and there may be more categories than we have, but it is important to decide upon some structure that everyone agrees upon for the project. This makes it much easier to find things.

We find it useful to locate the results of some subject-specific processing (e.g. co-registration of files, subject-level DTI processing, subject-level task fMRI processing)

```
/project_space/Udall
├── bin ............................ Holds bash scripts written for this project
│   ├── R ........................................................ Holds R scripts
│   └── tcl .................................................... Holds tcl scripts
├── data .............................. Other sources of data for all subjects
├── lib ............... Holds things that are used by many scripts and analyses
│   ├── makefiles ................................. Makefiles for this project
│   ├── networkROIs .................... A set of ROI masks for fMRI analysis
│   └── tractographyMasks ............ A set of masks for tractography analysis
├── freesurfer ........................... Freesurfer (cortical thickness) runs
├── tbss ................... Group-level Tract-Based Spatial Statistics analyses
└── subjects ............... All subject data collected in neuroimaging sessions
    ├── SUBJECT ............................................ A specific subject
    │   ├── session1 ................................ Difference subject sessions
    │   ├── session2
    │   └── session3
    │       ├── behavioral .................. Subject behavioral (e.g., task) data
    │       ├── Dicoms ........................................... Original dicoms
    │       ├── Parrecs ........ PAR/REC files, the native Philips scanner format
    │       ├── physio ...................................... Physiological data
    │       └── xfm_dir ..................................... Registration files
    ├── session3/SUBJECT ..... Symbolic link to the subject/session3 directories
    └── incoming .................. Zip files with subject data from the scanner.
```

Figure 2.2: An example of a project directory.

in the subject/session directories. However, other types of single-subject (and group) analyses are more conveniently performed in directories (e.g. `freesurfer/`, `tbss/`) that are stored at the top level of the project.

Data files that are collected from non-neuroimaging sources are usually kept in text (tab or comma-separated) form in the `data/` directory so that scripts can find and use them (e.g., to use the subject age or some cognitive variable as a regressor in an analysis). We have found it very useful to document, manage, and merge this data very carefully, including the important QA variables that describe whether or not a subject's scan should be included in analyses.

Finally, note that we have a directory labeled `incoming`. This is a scratch directory (it could be a symbolic link to a scratch drive) for placing zip files that come from the scanner, that contain the dicoms for the scan, the Philips-specific PAR/REC files, the physiological data logged (used to correct functional MRI data for heart rate and respiration-related signal) and data from scanner tasks (e.g., EPRIME files).

Setting up an Analysis Directory

Up until this point we have only seen little examples of makefiles that process subjects within a single directory. However, real studies include many subjects and many analyses; each analysis often produces hundreds of files per subject. Often, several researchers are trying to conduct different analyses on the same data set at the same time.

For these reasons, we use a collection of makefiles to manage a project. This section describes this basic recipe (which is a little complicated). You can also see this recipe implemented in `Udall`. Typically the steps involving creating subject directories and links are done by a script or an "incoming" makefile at the time that the subject data appears from the scanner. Better, if you use an archival system that you can write programs to access (for example, XNAT) a script can create all the links and name files nicely for you (see Downloading Data From XNAT).

This recipe relies on the concept of a "symbolic link." This is a file that points to another file or directory. A symbolic link allows you to access a file or directory using a different pathname. If you remove the link, you will not remove the thing that it points to. However, if you remove the target of the link, the link won't work any more.

To create a symbolic link, use the `ln` command.

```
$ ln -s target linkname
```

`target` is the path of the file you're linking to, and `linkname` is the name of the new symbolic link.

Target paths can either be absolute or relative, but they must be relative to their new location (in `linkname`). For example, using the directory structure in Figure 2.2, the command to create the link `session1/RC4101` would look like this (run from `Udall/session1`:

```
$ ln -s ../subjects/RC4101/session1 RC4101
```

Defining the basic directory structure

Let us assume the project home (`PROJHOME`) is called `$MAKEPIPELINES/Udall/`. There are three scans per individual. Let us also assume there are two subjects: RC4101 and RC4103. These are the directories that need to be in place. Note we organize the actual files by subject ID and scan session. However, to make processing at each cross-sectional point easier, we create directories with symbolic links to the correct

```
/project_space/Udall
 |__ subjects ............... All subject data collected in neuroimaging sessions
 |    |__ RC4101
 |    |    |__ session1..................... Subject-level processing happens here.
 |    |    |__ session2
 |    |    |__ session3
 |    |__ RC4103
 |         |__ session1
 |         |__ session2
 |         |__ session3
 |__ session1/RC4103............. Symbolic link to subjects/RC4103/session1.
 |__ session2/RC4101............. Symbolic link to subjects/RC4101/session2.
 |__ session2/RC4103........................................... and so on.
 |__ session3/RC4101
 |__ session3/RC4103
```

Figure 2.3: A longitudinal analysis directory.

subject/session directories. This flexibility helps in many ways to make cross-sectional and longitudinal analyses easier.

All simple subject-specific processing is well-organized within the subject/session directories (for example, skull-stripping, possibly first level FEAT, DTI analysis, co-registrations). We normally run FreeSurfer in a separate directory, because it likes to put directories in a single place. Analyses that combine data across subjects or timepoints (e.g. TBSS) best go in separate directories.

Figure 2.3 shows an example directory for a longitudinal study.

Creating the session-level makefiles

We will do all the subject-level processing from the `PROJHOME/sessionN/` directories. You will need to create a Makefile in `PROJHOME/session1`, `PROJHOME/session2`, and `PROJHOME/session3` whose only purpose is to run `make` within all the subject-level directories beneath it. Figure 2.4 shows the example session-level makefile (`Udall/subjects/makefile_session.mk`).

? #: the `make` comment character.

This Makefile obtains the list of subjects using a wildcard (so it expects to be in a directory where each subject has its own subdirectory). We will create symbolic links to this Makefile later.

The name of the subject directory is declared to be a phony target, so even if it exists, `make` will try to rebuild it. The recipe to do this is the very last line, which

```
# Top level makefile
# make all will recursively make specified targets in each subject
directory.
SUBJECTS=$(wildcard RC4???)
.PHONY: all $(SUBJECTS)
all: $(SUBJECTS)
$(SUBJECTS):
	$(MAKE) -directory=$@ $(TARGET)
```

Figure 2.4: Session-level Makefile

calls `make` recursively within each subject directory. It also passes along a `TARGET` variable. If unspecified, this would be the first target in the subject specific makefile.

Creating the common subject-level makefile for each session

As we described, the session-level makefile only exists to call `make` within all the subdirectories (symbolic links) in each session. So, we need to create a makefile within each subdirectory. However, we expect that the only thing that is different about each session is session-specific processing, which can be controlled by a `SESSION` variable. We can create a single subject-specific makefile and set up symbolic links to it just like we intend to do with the session-level makefile. Figure 2.5 is an example of a subject-level makefile that can be seen at `Udall/subjects/makefile_subject.mk`.

```
SESSION=$(shell pwd|egrep -o 'session[0-9]'|egrep -o '[0-9]')
subject=$(shell pwd|egrep -o 'RC4[0-9][0-9][0-9]')
test:
	@echo Testing that we are making $(subject) from session
$(SESSION)
```

Figure 2.5: Subject-level makefile

The subject-level makefile defines critical variables, such as the `SESSION`, which it obtains from the directory path, and the `subject` variable, which it also obtains from the directory path and a regular expression that matches the expected subject name. To set these variables we use the program `egrep`, which allows us to extract a specific pattern from the current working directory.

The only rule is a little dummy rule (because we have no actual data in this test directory) that ensures we have set these variables directly.

Creating links to the session-level makefile

Recall that the session-level makefile needs to be located within the directories `Udall/session1`,`Udall/session2` and `Udall/session3`. We could just copy it there, but then if we modified it in one place we would have to remember to change it everywhere. This would probably cause inconsistencies at some point.

Instead, we create a symbolic link to `makefile_session.mk` from each session directory as follows:

```
cd $PROJHOME/Udall/session1
ln -s ../subjects/makefile_session.mk Makefile
```

Figure 2.6: Creating symbolic links to the session-level makefile

Creating links to subject-level makefile

The last step now is to create a symbolic link within each subject directory to the appropriate subject-level makefile. For example, within the directory `subjects/RC4101/session1/`, we can type

```
$ ln -s ../../makefile_subject.mk Makefile
```

Running analyses

Once these steps are completed, you can conduct single-subject processing for each session by changing directory to the correct location and issuing the specific `make` command. Here, we illustrate how to make the "test" target for all subjects within `session2/`.

```
cd $PROJHOME/Udall/subjects/session2
make TARGET=test
```

Figure 2.7: Making `test`

This should generate output similar to that shown in Figure 2.8, showing that `make` goes in to each of the subject directories and creates the `test` target.

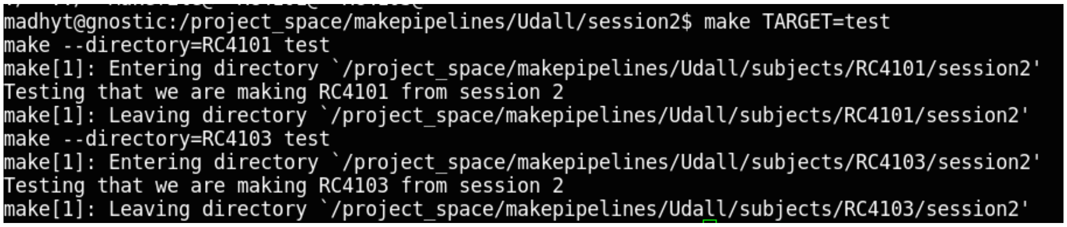

```
madhyt@gnostic:/project_space/makepipelines/Udall/session2$ make TARGET=test
make --directory=RC4101 test
make[1]: Entering directory `/project_space/makepipelines/Udall/subjects/RC4101/session2'
Testing that we are making RC4101 from session 2
make[1]: Leaving directory `/project_space/makepipelines/Udall/subjects/RC4101/session2'
make --directory=RC4103 test
make[1]: Entering directory `/project_space/makepipelines/Udall/subjects/RC4103/session2'
Testing that we are making RC4103 from session 2
make[1]: Leaving directory `/project_space/makepipelines/Udall/subjects/RC4103/session2'
```

Figure 2.8: Output of recursive make

Setting Important Variables

`make` uses variables to control a lot of aspects of its own behavior. We describe only a few here; see the GNU Make manual for the full list. However, it also allows you to set variables so that you can avoid unnecessary changes to makefiles when you move them to different projects. This is very important, because after going through the hassle of writing a makefile for an analysis once, we would like to reuse as much of it as possible for subsequent studies. It is reasonable to change the recipes to reflect the most appropriate scientific methods or new versions of software, but it's not fun to have to play with naming conventions, etc. We discuss some of the best practices we have found for using variables to improve portability.

Variables that control `make`'s Behavior

SHELL

By default the shell used by make recipes is `/bin/sh`. This shell is one of many that you can use interactively in Linux or MacOS, but it probably is not what you are using interactively (because it lacks nice editing capabilities and is less usable than other shells). Here, we set the default shell to `/bin/bash`, the same as what we use interactively, so that we can be sure when we test something at the command line that it will work similarly in a Makefile.

```
# Set the default shell
SHELL=/bin/bash
export SHELL
```

Figure 2.9: Setting `$(SHELL)`

Note that `SHELL` is accessed `$(SHELL)`, as are all `make` variables.

`TARGET`

In the strategy that we outline for organizing makefiles and conducting subject-level analyses, we run `make` recursively (i.e., within the subject directories) from the session-level directory. To do this very generally, we call make from the session-level by specifying the TARGET, or what it should "make" within the subject directory. You do not need to do this within the subject directory itself. For example:

(in `subjects/session1`)

```
$ make TARGET=convert_dicoms
```

(in `subjects/session1/s001`)

```
$ make convert_dicoms
```

`SUBJECTS`

We think it makes life easier to set this variable to the list of subjects in the study (or subjects for whom data has been collected). For example, given our directory structure, when in the top level for a session, the subject identifiers can easily be found with a wildcard on the directory names. The following statement sets the variable `SUBJECTS` to be all the six-digit files in the current directory (i.e., all the subject directories.)

```
SUBJECTS=$(wildcard [0-9][0-9][0-9][0-9][0-9][0-9])
```

`subject`

Often makefiles are intended to process a single subject. In this case, it is useful to set a subject variable to be the subject identifier.

`SESSION`

When subject data is collected at multiple time points, it is useful to set at `SESSION` variable that can be used to locate the correct subject files.

Other important variables

Ultimately, when it comes time to publish, it is important to state what version of the different software packages you have used. This means that you need to make

it difficult to accidentally run a job with a different version of the software. For example, consider this rule to run `DTIPrep`, a program for eddy correction, motion correction, and removal of noise from DTI images.

```
dtiprep/$(subject)_dwi_QCReport.txt: dtiprep$(subject)_dwi.nhdr
        DTIPrep --DWINrrdFile $< -p dtiprep/default.xml --default
--check --outputFolder dtiprep/
```

Figure 2.10: Running DTIPrep in make

Unless you take preventative measures, the version of `DTIPrep` that is used depends entirely on the caller's path. So if the version of `DTIPrep` on one machine is newer than that on another, results may differ. Alternatively, if my graduate student runs this makefile, and happens to have the newest version of DTIPrep installed in their own `bin/` directory, that is the version that will be used. This is a big problem for reproducibility.

A practical way to control for this is to specify the location of the program (if that conveys version information) as in Figure 2.11.

```
DTIPREPHOME=/usr/local/DTIPrep_1.1.1_linux64/DTIPrep
dtiprep/$(subject)_dwi_QCReport.txt: dtiprep$(subject)_dwi.nhdr
        DTIPrep --DWINrrdFile $< -p dtiprep/default.xml --default
--check --outputFolder dtiprep/
```

Figure 2.11: Controlling software version

What about when the programs are installed in some default location, e.g. `/usr/local/bin/`, with no version information? In our installation, this occurs frequently when using other workflow scripts that express pipelines more complicated than what might reasonably be put into a makefile.

In the case of simple scripts or statically linked programs, it is fairly easy to copy them into a project-specific bin directory, giving them names that indicate their versions. If you cannot do this, you need to check the version (if the program is kind enough to provide an option that will provide the version) or to check the date that the program was installed, to alert yourself to potential errors. It is useful to set variables for things like reference brains, templates, and so forth.

Variable overrides

It is probably a good idea not to edit a makefile too much once it works. But sometimes, it is useful to reissue a command with different parameters. Target-

specific variables may be specified in the makefile and overridden on the command line. In the example below, the default flags for FSL `bet` are specified as `-f .4 -B` .

```
BETFLAGS = -f .4 -B
%skstrip.nii.gz: %flair.nii.gz
        bet $< $@ $(BETFLAGS)
```

Figure 2.12: Specifying BET flags in make

However, these can be overriden from the command line as follows:

```
make BETFLAGS='-f .4 -R'
```

Suggested targets

These suggestions come from experience building pipelines. Having conventions, so that similarly named targets do similar kinds of things across different neuroimaging workflows, is rather helpful and comforting, especially when you spend a lot of time going between modalities and tools.

We propose splitting functions into multiple makefiles that can then be called from a common makefile. It is helpful to avoid overruling target names for common targets. See Processing Scan Data for a Single Test Subject for an example of how this is done in practice.

`all`

This is the default, the first target in the file. Nothing in this target should require human intervention.

`help`

We use a help system described by John Graham-Cumming, described in Advanced Topics & Quality Assurance with R Markdown, to document makefiles. You can approach any makefile by typing `make` and get a list of documented targets and their line numbers. From the programmer's perspective, it is easy to add documentation to a target, because the call to print help and the target are located right next to each other.

`clean`

Typically this target is used to remove all generated files (e.g., .o, dependency lists) and clean up the directory to its original state so that one can type "make" again and regenerate. So the idea is that "`make clean`" will clear the decks to allow you to restart the pipeline from scratch. This is particularly useful if you have accidentally

altered some files and would like to make sure that you know exactly what processing has occurred.

`mostlyclean` (or `archive`)
This is the same as clean, but does not remove things that are a pain to recreate (e.g., involve hand checking, or time-consuming analysis) or are critical results for publication. We use this because we are perpetually short of disk space, and this helps to clean up.

.PHONY

.PHONY (the period in front is necessary) is a special target used to tell `make` which targets are not real files. For example, common targets are `all` (to make everything) and `clean` (to remove everything). If you create a file named "all" or "clean" in the directory with the makefile, suddenly `make` will see that the target file "all" exists, and will not do anything if it is newer than its dependencies.

To stop this rather unexpected behavior, list targets that are not real files as .PHONY:

```
.PHONY: all clean anything_else_that_is_not_a_file
```

.SECONDARY

This target is used to define files that are created as intermediate products by implicit rules, but that you don't want deleted. This is critically important - perhaps a good philosophy is to define here all the files that are a pain to recreate. See Overview of `make` for a lesson on secondary targets.

.INTERMEDIATE

This target allows you to specify files that can be automatically deleted after the final targets are created. For example, during resting state preprocessing (Preprocessing Resting State Data) you create many intermediate files during the process (e.g., the output of motion correction, despiking). These are useful to check for QA purposes but in the end you may not want to keep them. Specifying them as intermediate targets will delete them after completion of the pipeline.

If you specify targets as intermediate, but you leave `.SECONDARY` blank, intermediates are treated as secondary and are not deleted automatically. However, if you delete them (e.g., in a `clean` target), they will not be recreated when you run make again so long as the targets depending on them already exist.

Chapter 3

Running `make` in Parallel

Although it is very powerful to be able to use makefiles to launch jobs in parallel, some care needs to be taken when writing the makefile, as with any parallel program, to ensure that simultaneously running jobs do not conflict with each other. In general, it is good form to follow these rules for all makefiles, because it seems highly likely that if you ignore them, there will come a deadline, and you will think to yourself "I have eight cores and only a few days" and you will run `make` in parallel, and all of your results will be subtly corrupted due to your lack of forethought, something you won't discover until you only have a few hours left. This is the way of computers.

Guidelines for Writing Parallelizable Makefiles

There are a few key things to remember when setting running `make` in parallel.

Each line in a recipe is, by default, executed in its own shell

This means that any variables you set in one line won't be "remembered" by the next line, and so on. The best thing to do is to put all lines of a recipe on the same line, by ending each of them with a semicolon and a backslash (`;\`). Similarly, when debugging a recipe in a parallel makefile gone wrong, look first for a situation where you have forgotten to put all lines of a recipe on the same line. For example, the recipe shown in Figure 3.1 will not work as intended, not matter what, because the first line of this recipe is not remembered by the second, and `brain.volume.txt` will be empty.

Instead, write the script as shown in Figure 3.2. Note the `;\` in red connects the two lines.

```
set foo=`fslmaths brain.nii.gz -V | awk 'print $$2'`
cat $$foo > brain.volume.txt
```

Figure 3.1: A non-functional multi-line recipe

```
set foo=`fslmaths brain.nii.gz -V | awk 'print $$2'` ;\
cat $$foo > brain.volume.txt
```

Figure 3.2: A now-functional multi-line recipe

You can also use `&&`, a `bash` operator that executes the next command only if the previous command was successful[1]. For example:

```
set foo=`fslmaths brain.nii.gz -V | awk 'print $$2'` &&\
cat $$foo > brain.volume.txt
```

Figure 3.3: A multi-line recipe using "`&&\`"

In this instance, `bash` will not attempt to `cat` the file if `fslmaths` or `awk` failed.

Filenames must be unique

It is very tempting to do something like the following, or the previous example, as you would while interactively using a shell script.

```
some_program > foo.out ;\
do something --now --with foo.out
```

Figure 3.4: How not to name files

This will work great sequentially; first `foo.out` is created and then it is used. You have attended to rule #1 and the commands will be executed together in one shell. But consider what happens when four processors run this recipe independently, in the same directory. Whichever recipe completes first will overwrite `foo.out`. This could happen at any time, so it is entirely possible that process A writes `foo.out` just in time for process B to use it. Meanwhile, process C can come along and rewrite it again. You see the point. The best way to avoid this problem, no matter how the

[1] In other words, has exited with an exit status (`$?`) of "0."

makefile is used, is to always create temporary files (like `foo.out`) that include a unique identifier. Alternately, you could use a subject identifier to form the name. For longitudinal analysis we play it safe and often use a timepoint identifier as well. Thus, whether you conduct your analysis using one subdirectory per subject or in a single directory for the entire analysis, you can take the recipe you have written and use it without modification. Another approach is to create unique temporary names using the `mktemp` program and delete them when you are finished.

Many neuroimaging software packages use the convention that files within a subject-specific directory that contains a subject identifier can have the same names. In that case, just go with it: They have done that in part to avoid this confusion while running the software in parallel on a shared filesystem.

Separate time-consuming tasks

Try to separate expensive tasks into seperate recipes. Suppose you have two time-consuming steps: `run_a` and `run_b`. `run_a` takes half an hour to generate `a.out` and `run_b` takes an hour to generate `b.out`. Additionally, `run_a` must complete before the other can begin. Examine the rule in Figure 3.5.

```
a.out b.out: b.in
        run_a ;\
        run_b
```

Figure 3.5: The wrong way to run two long tasks

This sort of works, but suppose another task only needs `a.out`, and doesn't depend at all on `b.out`. You would spend a lot of extra time generating `b.out`, especially if this was a batch job. A worse problem is that you have specified two targets, `a.out` and `b.out`. This is like reproducing this rule twice. If you run in parallel this rule will fire twice, once to create `a.out` and once to create`b.out`, and you will spend twice as much effort as you need to (but in parallel). So it is better for many reasons to write the rule in two separate lines, as in Figure 3.6.

```
a.out: b.in
        run a
b.out: a.out
        run_b
```

Figure 3.6: The right way to run two long tasks

Executing in Parallel

Using multiple cores

Most modern computers have multiple processing elements (cores). You can use these to execute multiple jobs simultaneously by passing the `-j` option to `make`. You can either specify the number of jobs after `-j` or you can omit the argument and let it use the maximum number of cores available.

It is good to know the number of cores on your machine. The command `nproc` might work, or you can ask your system administrator.

You have to be careful not to start a lot more jobs than you have cores, other wise the performance of all the jobs will suffer. Let us assume there are four cores available. Pass the number of jobs to `make`.

```
$ make -j 4
```

Now you will execute the four cores simultaneously. This will work well if no one else is using your machine to run jobs. For this reason, you may want to specify that `make` should use fewer cores than available so that you can still get good response time on the machine.

Recall that `make` will die when it encounters an error. When running jobs in parallel, `make` can encounter an error that much faster. If one job dies, all the rest will be stopped. This is rarely the behavior you want, because typically each job is independent of the others. To tell make not to give up when the going gets tough, use the `-k` (keep going!) flag.

```
$ make -j 4 -k
```

Using the grid engine

Four cores (or even eight or 12 or 24) is nice, but a cluster is even nicer. A cluster gangs together several multi-core machines to act like one. At IBIC, our cluster environment is the Sun Grid Engine (SGE) which is a batch queuing system. This software layer allows you to submit jobs, queue them up, and farm them out to one of the many machines in the cluster. Different sites may be configured differently, so check with your system administrator.

! In IBIC, you can submit jobs from any machine in a grid engine. In IBIC, `pole` and `pons` are easy to type.

Setting FSLPARALLEL for FSL jobs

There are two ways to use `make` to run jobs in parallel on the SGE. The first is to use the scripts for parallelism that are built in to FSL. In our configuration, all you need to do is set the environment variable `FSLPARALLEL` from your shell as follows:

```
$ export FSLPARALLEL=true
```

This must be done before running `make`! Then, you run your makefile as you would on a single machine, on a machine that is allowed to submit jobs to the SGE (check with your system administrator to find out what this is). What will happen is that the FSL tools will see that this flag is set, and use the script `fsl_sub` to break up the jobs and submit them to the SGE. You do not need to set the `-j` flag as above, because FSL will control its own job submission and scheduling.

Note that this trick will only work if you are using primarily FSL tools that are written to be parallel. What happens if you want to use something like `bet` on 900 brains (which is not parallelized), or other tools that are not from FSL?

Using qmake

By using `qmake`, a program that interacts with the SGE, you can automatically parallelize jobs that are started by a makefile. This is a useful way to structure your workflows, because you can run the same neuroimaging code on a single core, a multicore, and the SGE simply by changing your command line. You may need to discuss specifics of environment variables that need to be set to run `qmake` with your system administrator. If you are using `make` in parallel, you also will probably want to turn off `FSLPARALLEL` if you have enabled it by default.

There are two ways that you can execute qmake, giving you a lot of flexibility. The first is by submitting jobs dynamically, so that each one goes into the job queue just like a mini shell script. To do this, type

```
$ qmake -cwd -V -- -j 20 all
```

The flags that appear before the "--" are flags to `qmake`, and control grid engine parameters. The `-cwd` flag means to start grid engine jobs from the current directory (useful!) and `-V` tells it to pass all your environment variables along. If you forget the `-V`, we promise you that very bad things will happen. For example, FSL will crash because it can't find its shared libraries. Many programs will "not be found" because your path is not set correctly. Your jobs will crash, and that earthquake will kill all of us.

On the opposite side of the "--" are flags to make. By default, just like normal make, this will start exactly one job at a time. This is not very useful! You probably want to specify how much parallelism you want by using the -j flag to make (how many jobs to start at any one time). The above example runs 20 jobs simultaneously. The last argument, all, is a target for make that is dependent upon the particular makefile used.

One drawback of executing jobs dynamically is that make might never get enough computer resources to finish. For this reason, there is also a parallel environment for make that reserves some number of processors for the make process and then manages those itself. You can specify your needs for this environment by typing

```
$ qmake -cwd -V -pe make 1-10 -- freesurfer
```

This command uses the -pe to specify the parallel environment called make and reserves 10 nodes in this environment. The argument to make is freesurfer in this example. Note that we do not use this environment in IBIC.

How long will everything take?

A good thing to do is to estimate how long your entire job will take by running make on a single subject and measuring the "wall clock" time, or the time that it takes between when you start running it and when it finishes. If you will be going home for the night, add a command to print the system date (date) as the last line in the recipe, or look at the timestamp of the last file created. Suppose one subject takes 12 hours. Probably other subjects will take, on average, the same amount of time. So you multiply the number of subjects by 12 hours, and divide by 24 to get days. For 100 subjects, this job would take 50 days. This calculation tells you that it would be a long time to wait for your results on your four-core workstation (and in the meantime, it would be hard to do much else).

Suppose you have a cluster of 75 processors, and 100 subjects. If you can use the entire cluster, you can divide the number of subjects by processors and round up. This gives you two. If you multiply this by your average job time, you find that you can complete this job on the cluster in one day. If you think about this, you see that if you had 100 processors, you could finish in half the time (12 hours) because you would not have to wait for the last 25 jobs to finish.

Consideration of memory

Processing power is not the only resource to consider when running jobs in parallel. Some programs (for example, bbregister in our environment) require a lot of memory. This means that attempts to start more jobs than the available memory

can support will cause the jobs to fail. A good thing to do is to look at the system monitor while you are running a single job, and determine what percentage of the memory is used by the program (find a computer that only you are using, start the monitor before you begin the job, and look at how much additional memory is used, at a maximum, while it runs). If you multiply this percentage by the number of cores and find that you will run out of memory, do not submit the maximum number of jobs. Submit only as many as your available resources will support.

Troubleshooting `make`

One flaw of `make` (and indeed, many UNIX and neuroimaging programs and just life in general) is that when things do not go as expected, it is difficult to find out why. These are some hints and tricks to help you to troubleshoot when things go wrong.

Find out what `make` is trying to do

Start from scratch, remove the files you are trying to create, then execute `make` with the `-n` flag.

```
$ make -n all
```

You can also place the flag at the end of the command. This way, it is easy to hit ↑ and delete `-n` without having to muck about arrowing over to the flag placed at the beginning of the command. By doing this you could save seconds a day!

```
$ make all -n
```

The `-n` flag shows what commands will be executed without executing them. This is very handy for debugging problems, as it tells you what make is actually programmed to do. Remember, computers do exactly what you tell them.

Use the trace option in `make` 4.1

Debugging is such an issue that the latest version of GNU `make`, version 4.1, has tracing functionality to show you what is going on. Even if your system does not have this installed, you can download it and run it with the `-trace` option. Note that there are some subtle differences between `make` versions. For a thorough desciption of version differences and more debugging options, see (Graham-Cumming, 2015).

Check for line continuation

Ensure that you have connected subsequent lines of a recipe with a semicolon and a backslash (;\). If you don't do this, each line will be run in a separate shell, and variables from one will not be readable in the other.

No rule to make target!

Suppose you write a makefile and include in it what you think should be a rule to create a specific file (say, `data_FA.nii.gz`). However, when you type `make`, you get the following message:

```
make:  *** No rule to make target `data_FA.nii.gz', needed by `all'
```

This can be rather frustrating. There are multiple ways this error message could be generated. We recommend the following steps to diagnose your makefile.

1. Read the error message.
   Seriously. It is tempting to try to read a program's mind, but inevitably this fails. Mostly because they don't have minds. In the error above, the key aspects of the error above are as follows:
   (a) The error comes from `make`, as you can see by the fact that the line starts with `make:`. It is also possible that your Makefile fails because a program that you called within it fails (and you either did not specify the `-k` flag to keep going, or there is nothing left to do).
   (b) `make` claims that there is "no rule" to make the target "`data_FA.nii.gz`." This tells you that `make` could not find a target: dependency combination to create this particular target.
   (c) `make` is trying to create this target to satisfy the target `all`. This tells you that it was trying to make `data_FA.nii.gz` because this is a dependency of the target `all`.
2. If your error is indeed coming from `make`, then try to pinpoint the rule that is failing. *Look* at your makefile and check that the rule looks correct. Do all the dependencies of the rule (the things to the left of the colon) exist? Are they where they should be? Can you read them?
3. If everything looks ok, you are missing something. Rules that expand patterns and that use variables can be tricky to interpret (the same way that you generally like a debugger to look at code). To see what make is really doing with your rules, use the `-p` flag to print the database and examine the rules. We suggest doing this in conjunction with `-n` so that you do not actually execute anything.
4. Pattern matching has an odd behavior where, if there is no rule to create a dependency, it will tell you there is no rule to create the target.

For example, Figure 3.7 will fail with

```
...  No rule to make target `<subj>_T1_brain.nii.gz' ...
```

not

```
...  No rule to make target `foo' ...
```

```
%_T1_brain.nii.gz: %_T1.nii.gz foo
	bet $< $@
```

Figure 3.7: Pattern-matching error handling

Suspicious continuation in line

If you get this error while trying to save a makefile (using `emacs`, which is smart enough to help you ensure the syntax of your makefile is correct), it means that you probably have a space after the trailing `;\` at the end of the line. No, you can't have indiscriminate whitespace in a makefile!

`make` keeps rebuilding targets

A more subtle problem occurs when your makefile works the first time, but it also "works" the second time ... and the third ... and so on. In other words, it keeps rebuilding targets even when they do not "need" to be rebuilt. This means that `make` examines the dependencies of the target, decides that they are newer than the target (or that the target does not exist) and executes the recipe.

This cycle never stops if, somewhere, a target is specified that is never actually created (and that is not marked as phony). This is easy to do, for example, when trying to execute more complex neuroimaging pipelines (such as those provided by FSL) that create entire directories of files with specific naming conventions. Check that the commands that keep re-executing really create the targets that would indicate that the command has completed. For example, when running `feat` with a configuration file, the name of the output directory is specified in the configuration file. This must be the same as the target in the makefile, or it will never be satisfied.

`make` doesn't rebuild things that it should

This type of error usually indicates a logical problem in the tree of targets that begins with what you have told make to do. Recall that by default, `make` builds the first target that appears in the makefile. If you have included makefiles at the beginning of your file, it might happen that the default target is not actually what you think it is.

Chapter 4

Recipes

This section contains recipes for different kinds of small tasks you may wish to accomplish with `make`. Some of these recipes can be seen "in action" in real makefiles in the example data supplement to this manual. These real example makefiles are documented in Examples. When we refer to these files, we will assume that the path is relative to where you have unpacked these examples in your environment.

Obtaining a List of Subjects

A common thing you may want to do in a Makefile is to set a variable that includes the names of all subjects that you will be processing. Here are some examples of code to set the `SUBJECTS` variable. This first approach (Figure 4.1) keeps a list of subjects in a file called `subject-list.txt`. This is extremely handy for analyses that use only a subset of the subjects: for example, only a treatment group, or a selected subset of subjects from the entire study. For an example of this approach in use, see Downloading Data From XNAT.

```
SUBJECTS=$(shell cat subject-list.txt)
```

Figure 4.1: Obtaining a list of subjects from a file.

This second approach (Figure 4.2) simply uses the project directory as reference, finding all the subject directories it can, and then assumes if a subject directory exists, so does the data to run this analysis. For an example of this approach in use, see `oasis-longitudinal-sample-small/visit1/Makefile`, described in Practical 2.

Both approaches are appropriate for different circumstances. For example, we have used the first approach for a pilot study where several subjects in the pilot

```
SUBJECTS = $(wildcard OAS2_????)
```

Figure 4.2: Obtaining a list of subjects using a wildcard.

had some technical problems with their ASL scans. They were perfectly usable for other purposes but not for the ASL analysis. We have used the second approach for keeping up with FreeSurfer analyses of cortical thickness, running FreeSurfer on new subjects as soon as their dicoms are converted to nifti files and their T1 image is placed in their subject directories.

Setting a variable that contains the subject id

It is useful to set a variable that contains the subject identifier, to be used when naming or accessing files that incorporate the subject identifier. We assume that this is in the context of subject-specific processing within a directory. One way to do this robustly (so that it works whether or not you are accessing the subject directory via a symbolic link or from the directory itself) is as shown in Figure 4.3. For an example of this approach in use, see `oasis-longitudinal-sample-small/visit1/Makefile.subdir`, described in Practical 2.

```
subject=$(shell pwd | egrep -o `OAS2_[0-9]*')
```

Figure 4.3: Determining the subject name from the current working directory and a pattern.

By using the command `grep` and a pattern to identify the subject identifier from the current working directory, this method will work no matter where in the path the subject identifier is located.

Using Conditional Statements

Setting a conditional flag

Sometimes we wish to process data only if we have the correct file (or files). By design or by accident, it may be that a certain scan was not acquired or is not of sufficient quality to process. Here, we have created a file `00_NOFLAIR` for each subject data directory that is missing a Fluid Attenuated Inversion Recovery (FLAIR) scan sequence. We set a variable called `HAVEFLAIR` that we can later use to “comment out” commands that would fail miserably if the basis files do not exist (Figure 4.4). To see this in use, see `testsubject/test001/Makefile`, described in Practical 3.

```
HAVEFLAIR = $(shell if [ -f 00_NOFLAIR ] ; then echo false; else
echo true; fi)
```

Figure 4.4: Setting a variable to determine whether a FLAIR image has been acquired.

We could also just look for the FLAIR image and decide that if it isn't there, we shouldn't try to process it (not throwing an error). With hundreds of subjects, however, it takes a lot of time to cross reference the master spreadsheet to check whether the file is missing by design or whether it did not get copied correctly. Making little marker files such as `00_NOFLAIR` (which can contain missing data codes and reasons for the missing data) helps a lot to avoid re-checking.

Another example is a multisite study that occurs at several sites, each with different scanners. We may want to set a site variable that we can use to perform conditional processing (for example, adjusting for different sequence flags). Here, we create a site file called `00_XX`, where the XX is a two letter code for the specific site. The site variable is then set to `00_XX` (Figure 4.5).

```
SITE = $(shell echo 00_??)
```

Figure 4.5: Setting a variable to indicate the study site.

Finally, DTI Distortion Correction with Conditionals illustrates how to perform conditional processing based on the existence of specific files. In that example, the presence of certain files triggers different streams of diffusion tensor imaging (DTI) processing. This is a useful strategy for programming a makefile to be portable across different common acquisitions.

Using a conditional flag

Once set, it is possible to test a conditional flag and perform different processing. Having set a conditional flag, it can be used to select rules in your makefile. Here, for example, we use the `SITE` variable to reorient and rename the correct T1 image (which has a different name depending on the different sequence/scanner used at each site).

Conditional execution based on the environment

By default, environment variables set in the shell are passed to `make`. These variables may be overridden within a makefile. In this example (Figure 4.7), used in many of the example data makefiles, we check whether users have set an environment variable

```
ifeq ($(SITE),00_BN)
$(subject)_T1.nii.gz: $(wildcard *SCALP.nii.gz)
        fslreorient2std $< $@
endif
ifeq ($(SITE),00_MR)
$(subject)_T1.nii.gz: $(wildcard *t1_fl3d_SAG.nii.gz)
        fslreorient2std $< $@
endif
```

Figure 4.6: Testing the site variable to determine the name of the T1 image.

`MAKEPIPELINES`. This is used to refer to files within the example directories. If it is undefined it is set to the default IBIC location (so that IBIC users can try out the makefiles without worrying about setting the variable).

```
ifeq "$(origin MAKEPIPELINES)" "undefined"
MAKEPIPELINES=/project_space/makepipelines
endif
```

Figure 4.7: Testing to see whether an environment variable is undefined

Conditional execution based on the Linux version

In our environment we strive to run the exact same image of the Linux operating system, with all the same software packages installed, on all computers. However, we often migrate workstations to the latest version of the operating system over a period of several months. Different groups time their upgrades to minimally disturb image processing. In one case, there was a particular AFNI program necessary for our resting state analysis that was only installed in the latest OS. So, we check the version of the OS in order to conditionally set the targets that the makefile will build (Figure 4.9).

On IBIC workstations, operating system information is recorded in `/etc/os-release`. (See Figure 4.8.)

`source` this file in order to save the strings to the given variables, which can then be read easily in `make`. For example, one could test if the newest version of Debian – 7 (wheezy) – is installed on the current workstation.

! Not all systems use this file. Ask your system administrator what program or file to use if you cannot find it.

```
PRETTY_NAME="Debian GNU/Linux 7 (wheezy)" NAME="Debian
GNU/Linux" VERSION_ID="7" VERSION="7 (wheezy)" ID=debian
ANSI_COLOR="1;31" HOME_URL="http://www.debian.org/"
SUPPORT_URL="http://www.debian.org/support/" BUG_REPORT_-
URL="http://bugs.debian.org/"
```

Figure 4.8: `/etc/os-release`

```
SHELL=/bin/bash
RELEASE=$(shell source /etc/os-release && echo $$VERSION_ID)
ifeq ($(RELEASE),7)
all: processedrest_medn.nii.gz
else
all:
	@echo "The software to run this pipeline is only installed
on"
	@echo "the newest version of the OS, 7 (wheezy)"
endif
```

Figure 4.9: Testing the Linux version to determine whether to proceed

Part II

Practicals

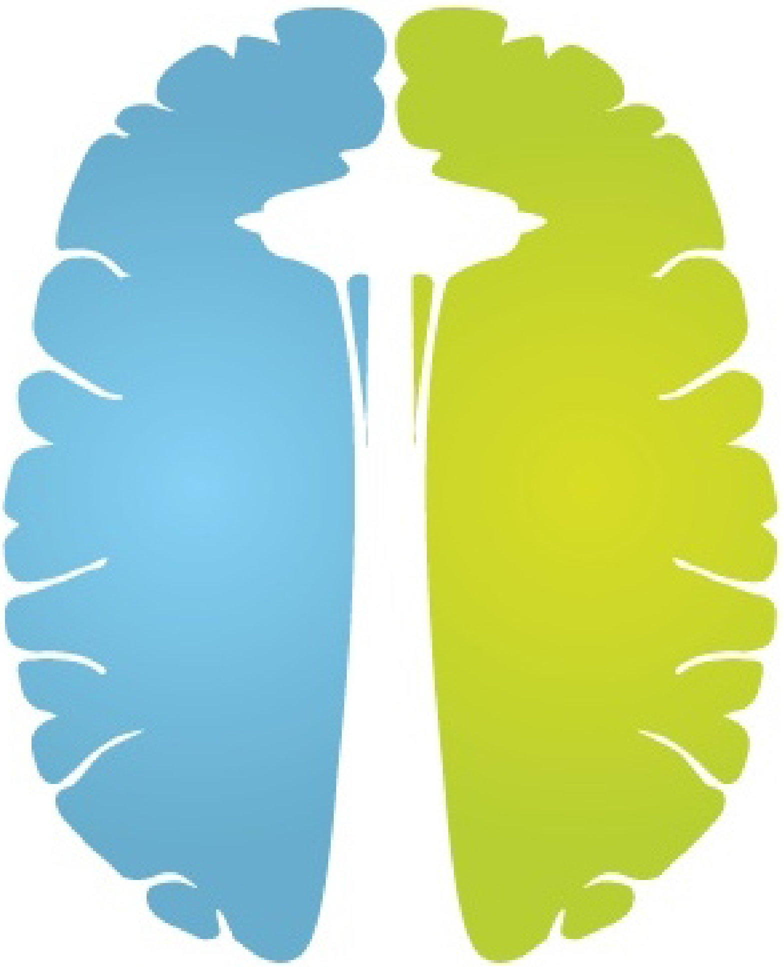

Part II will guide you through four `make` classes, using data available on the IBIC system and on NITRC. Each class is structured as a brief lecture followed by a practical designed to be completed as you read the chapter. Note that in Practicals and in Examples, Makefiles are printed with a yellow background so that they can easily be identified.

Practical 1

Overview of `make`

Learning objectives: Obtain a conceptual understanding of `make`, understand the basic syntax of makefiles and pattern rules.

Lecture: The Conceptual Example of Making Breakfast

`make` is a tool for implementing a workflow, or a sequence of steps that you need to execute. Probably the way that most of you have been implementing workflows up until this point is with some kind of program, possibly a shell script. The goal of this lecture and practicum is to motivate the reasons why you might want to move from a shell script to some language for better specifying workflow.

A few elements of good workflow systems are:

- Reproducibility, which a shell script can provide.
- Parallelism, which a shell script cannot provide.
- Fault tolerance, which a shell script can absolutely not deal with. If there is a problem in subject nine of your 100-subject loop, subjects 10-100 will not even begin, even if nothing is wrong with them.

We will start with a conceptual example of the difference between shell scripts and `make`. `make` organizes code into blocks called "recipes," so for our conceptual example, we will create a "waffwich", a delicious[1] breakfast food that one can eat with one hand on the way to the bus stop. We can create a pseudocode example of how to make a waffwich (Figure 1.1).

! Pseudocode does not follow any real programming syntax

This "script" tells you very neatly what to do. However it enforces a linear ordering on the steps that is unnecessary. If you had lots of sandwich-making resources you could use, you would not know from this description that the sandwiches do not have to be made sequentially. You could, for example, toast all the waffles for each person

[1] So I'm told.

```
for person in a b c
do
toast waffle
spread peanut_buttter --on waffle
arrange berries --on waffle --in squares --half
cut waffle
fold waffle
done
```

Figure 1.1: Creating a waffwich

before spreading the peanut butter on them. You could make person c's waffwich before person a's, but the script does not specify this flexibility.

You also would have no way of knowing what ingredients the waffwich depends on. If you execute the steps, and realize you don't have any berries, your script will fail. If you go out to the store and get berries, you would have to rewrite your script to selectively not retoast and spread peanut butter on the first person's waffle, or you would just have to do everything again.

This is a conceptual example, but in practice, having this information in place saves a whole lot of time. In neuroimaging, because we often process many subjects simultaneously, exploiting parallelism is essential. Similarly, there are often failures of processing steps (e.g., registrations that need to be hand-tuned, tracts that need to be adjusted). With higher-resolution acquisition, jobs run longer and longer, so the chance of computer failure (or a disk filling up, or something) during the time that you run a job is more likely. But all this information cannot be automatically determined from a shell script.

There has been a lot of work done on automatically inferring this information from languages such as C and MATLAB, but because shell scripts call other programs that do the real work, there is no way to know what inputs they need and what outputs they are going to create.

The information in Figure 1.1 can alternatively be represented as a directed acyclic graph (Figure 1.2).

Note that there are no circular references in this graph! (Hence, it is called an "acyclic" graph.) Also note that every arrow points in one direction (to what it depends upon). This is what makes this graph "directed". A graph like this is a very good way of describing a partially ordered sequence of steps. However, drawing a graph is a really nasty way of writing a program, so we need a better syntax for making dependencies. The `make` recipe for a waffwich would look something like the example below.

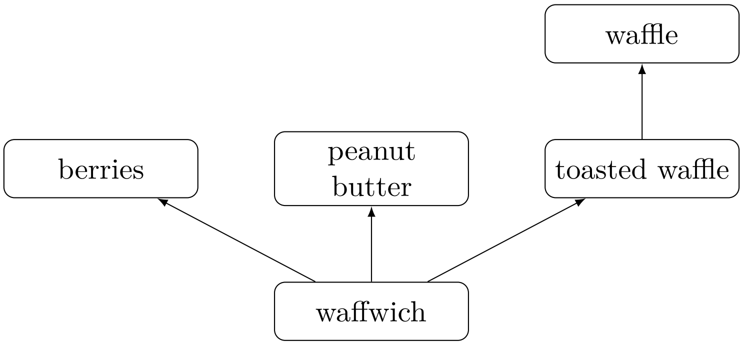

Figure 1.2: How to make a waffwich

```
waffwich: toastedwaffles berries BP
        spread PB --on waffle
        arrange berries --on waffle --in squares --half
        cut waffle
        fold waffle

toastedwaffle: waffle
        toast waffle
```

We must note: makefiles are not linear, unlike shell scripts. `toastedwaffle`[2] needs to be created before `waffwich`, but does not need to appear before it in the script. `make` will search for the targets that it needs to create in whatever order it needs to find them.

However, we've reached the point where we have to leave this conceptual example because `make` is aware of whether something exists or not, and whether it has to make it. It does this by assuming that the target and dependencies are files, and by checking the dates on those files. There are many exceptions to this; but let's accept this simplification for the moment, and move on to a real example with files.

Practical Example: Skull-stripping

Manipulating a single subject

Follow along with this example. Copy directory `$MAKEPIPELINES/oasis-multisubject-sample/` to your home directory. You can do this with the following command:

[2] Indeed, two instances of a toastedwaffle

```
$ cp -r $MAKEPIPELINES/oasis-multisubject-sample ~
```

This is a tiny sample of subjects from the OASIS data set (`http://www.oasis-brains.org`). Your task is to skull strip all of these brains. However, note that first you need to reorient each image (try looking at it in `fslview`). So there are two commands you need to execute:

```
$ fslreorient <subject>_raw.nii.gz <subject>_T1.nii.gz
```

and

```
$ bet <subject>_T1.nii.gz <subject>_T1.skstrip.nii.gz
```

The first command reorients the image and the second performs the skull stripping. By default, `make` reads files called “Makefile.” According to the manual, GNU looks for `GNUmakefile`, `makefile`, and `Makefile` (in that order) but if you stick to just one convention, you are unlikely to get confused.

Open the makefile (`Makefile`)that came with the directory with an editor. You should see the following:

```
OAS2_0001_T1_skstrip.nii.gz: OAS2_0001_T1.nii.gz
        bet OAS2_0001_T1.nii.gz OAS2_0001_T1_skstrip.nii.gz

OAS2_0001_T1.nii.gz: OAS2_0001_raw.nii.gz
        fslreorient2std OAS2_0001_raw.nii.gz OAS2_0001_T1.nii.
            gz
```

Play around with this. What happens when you execute `make` from the command line? What is it really doing?

Change the order of the rules. What happens? Note that by default, `make` starts by looking at the first target (the first thing with a colon after it). That is why, when you change the order of the rules, you get different outcomes, but *not* because it is reading them one by one. It’s creating a directed acyclic graph, but it has to start somewhere.

You can also tell it where to start. Delete any files that you may have created. Leave the makefile with the rules reordered (so that the `fslreorient2std` rule is first). Now type:

```
$ make OAS2_0001_skstrip.nii.gz
```

Now make starts with this target and goes on from there, working backward to figure out what it needs to do.

Pattern rules and multiple subjects

This is great, but so far we have only processed one subject. You can create rules for each subject by cutting and pasting the two rules you have and editing them. But that sounds like a huge chore. And what if you get more subjects? Yuck.

You can specify in rules that you want to match patterns. Every file begins with the subject identifier so you can use the % symbol to replace the subject in both the target and the dependencies. However, what do you do in the recipe when you don't know the actual names of the file you're presently working with? The % won't work there. However, the symbol $* does, as you can see in the next example.

! Patterns can be matched anywhere in the filename, not just at the beginning.

```
%_T1_skstrip.nii.gz: %_T1.nii.gz
        bet $*_T1.nii.gz $*_T1_skstrip.nii.gz

%_T1.nii.gz: %_raw.nii.gz
        fslreorient2std $*_raw.nii.gz $*_T1.nii.gz
```

Using the $* to stand in for patterns can get visually ugly. One common shortcut is to use the automatic variable $@ to replace the target, and $< for the first dependency.

Using these new variables, our code can now be written as in this example.

```
%_T1_skstrip.nii.gz: %_T1.nii.gz
        bet $< $@

%_T1.nii.gz: %_raw.nii.gz
        fslreorient2std $< $@
```

But try executing make now, with just those rules. (If you are completely lost now, look at the file Makefile.1 to see what your makefile should look like). You should get an error that there are no targets. Note that % does not work as a wildcard as in the bash shell. If you are used to that behavior, you might expect make will sense that you have a lot of files with subject identifiers, and it should automatically expand the % to match them all. It will not do that, so you have to be explicit about what you want to create.

For example, try typing:

```
$ make OAS2_0001_T1_skstrip.nii.gz
```

Now make has a concrete target in hand, and it can go forth and figure out if there are any rules that match this target (and there are!) and execute them.

`make` will first look for targets that match exactly, and then fall through to pattern matching.[3]

It can be helpful to visualize pattern matching as a sieve that catches and "knocks off" the part that it matched, leaving only the stem. It will additionally strip any directory information when present. For example, `foo/%_T1.nii.gz: %.nii.gz` will work just as you would like.

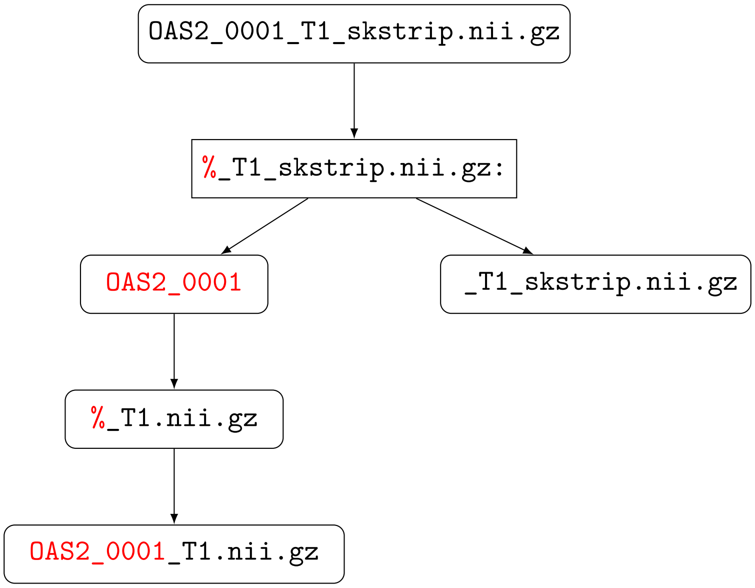

Figure 1.3: Pattern-matching as a sieve

Figure 1.3 shows how `make` identifies a pattern from an input and applies it to match the dependencies. Here, you can see that it is important to not include trailing (or leading!) underscores or other characters in your expected pattern.

Phony targets

Great, but how do you specify that you want to build all of these subjects? You can create a phony target (best placed at the top) that specifies all of the targets you really want to make. It is called a phony target because it does not refer to an actual file.

```
skstrip: OAS2_0001_T1_skstrip.nii.gz OAS2_0002_T1_skstrip.nii.
  gz
```

[3]It will use more specific rules before more generic ones: see the GNU `make` manual for more information on this behavior.

Add as many subjects as you're patient enough to type and execute

```
$ make skstrip
```

Of course, typing out all the subjects (especially with ugly names like `OAS2_0001_T1_skstrip.nii.gz`) is almost as time-consuming as copying the rule multiple times. Thankfully, there are a lot of ways to create lists of variables, shell commands, wildcards, and most other things you might think of. Conveniently, there is a file called `subjects` in this directory. We can get a list of subjects by using the following `make` command.

```
SUBJECTS=$(shell cat subjects)
```

Now we can write a target for skull-stripping.

```
skstrip: $(SUBJECTS:=_T1_skstrip.nii.gz)
```

You must tell `make` that `skstrip` is not a real file.[4] Do this by using the following statement:

```
.PHONY: skstrip
```

The pattern substitution for changing subjects to files comes from Section 8.2 of the GNU `make` manual. You can look up the other options, but the basic function of this command is to add the affix `_T1_skstrip.nii.gz` to each item in `SUBJECTS`.

The command uses the more general syntax, which can be used to remove parts of names before adding your new affix.

```
$(var:suffix=replacement)
```

As an esoteric example, this command could be used to replace all the final 5s with the string "five."

```
$(SUBJECTS:5=five)
```

`make` should now fail and tell you that there is no rule to make the target `OAS2_00five_T1.nii.gz`.

Secondary targets

Now if you type `make` here it is going to go through and do everything, if there's anything to do. But it's easier to see the point of secondary targets if you use the `-n` flag for `make`, which asks `make` to tell you what it's going to do, without actually doing it.[5] It's puzzling, isn't it, that all the T1 files are deleted? What's wrong here?

[4] Otherwise, if someone were to create a file named `skstrip` in this the directory, the makefile would stop working as expected.

[5] Trevor likes to use `make <cmd> -n`, which makes it easier to hit ↑ and remove the flag without any annoying arrowing around.

Because we explicitly asked for the skull-stripped brains, `make` figured out it needed to make the T1 brains. This is an *implicit* call. `make`, once it has finished running, goes back and erases anything that was implicitly created. This is a good thing, or a weird thing, depending on how you look at it. You can specify to `make` to keep those intermediary targets by adding it to the `.SECONDARY` target, like so:

```
.SECONDARY: $(SUBJECTS:=T1.nii.gz)
```

An alternative method is explicitly add targets you want to keep to the phony target.

```
skstrip: $(SUBJECTS:=_T1_skstrip.nii.gz) $(SUBJECTS=_T1_skstrip
   .nii.gz)
```

This is a little easier to interpret, as well. It also provides the advantage of allowing `make` to realize when an intermediary target needs to be remade. For example, if you erased only a T1 image (perhaps to rerun `bet`), and told it to `make skstrip`, it would look and see that all the `T1_brain` images are newer than the raw images, and therefore, no change needs to be made. While this problem could be solved by also removing the `T1_brain` image, that's more work and we want to do as little of that as possible.

make clean

What if you want to clean up your work? You've been deleting files by hand, but with phony targets, you don't have to. A common approach is to create a target called `clean`, which removes everything that the makefile created to bring the directory to its original state.

```
clean:
	rm -f *_T1.nii.gz *_T1_skstrip.nii.gz
```

Note that in the recipe, it's totally fine to use wildcards. Depending on how far along you got with your processing before you decide to clean and restart, not every file might have been created. For this reason we typically use the `-f` flag for `rm`. This flag hides error messages from attempting to erase nonexistent files.

The convention of putting a target for `clean` is just a convention; you need to muster the discipline to add every new file that you create in a makefile to the clean target. More dangerous is that sometimes to get a neuroimaging subject to a certain state, you need to do some handwork that would be expensive to recreate. For example, no one wants to blow away FreeSurfer directories after doing hand editing.

For these reasons, we like the looser convention of `mostlyclean` or `archive` which means to remove things that can easily be regenerated, but to leave important partial products (e.g., your images converted from dicoms, FreeSurfer directories, final masks, and other things).

Do not forget to add `clean`, `mostlyclean` and any other phony targets that you find useful to your list of phony targets:

```
.PHONY: skstrip clean
```

Compare your final makefile to the finished version in `Makefile.2` and you are done with the first practical.

Practical 2

`make` in Context

Learning objectives: Organize a subject directory, run make in parallel.

Lecture: Organizing Subject Directories

In practice, a project will involve multiple subjects, each with several types of scans. One could imagine a universal standard where every NIfTI file contained information about where it came from and what information it contained, and all neuroimaging programs understood how to read and interpret this information. We could dump all of our files into a database and extract exactly what we wanted. We wouldn't need to use something as archaic as `make` because we could specify dependencies based on the products of certain processing steps, no matter what they were called.

If you can't imagine this, that's totally fine, because it's a very long way off and won't look like that when it's here. Right now, we need to work with UNIX file naming conventions to do our processing.

Therefore, selecting good naming conventions for files and for directories is key. `make` specifically depends upon naming conventions so that people can keep track of what programs and steps were used to generate what projects. See Running `make` in Context for examples of good file naming conventions and typical project directory structures.

Many of our studies are longitudinal. Even if they don't start out that way, if you are trying to study a developmental or degenerative disease, and you scan subjects once, it is often beneficial to scan them again to answer new questions about longitudinal progression. However, this aspect of study design poses some challenges for naming conventions.

Different sites organize data in different ways. For example, the Waisman Brain Imaging Lab has a detailed description of their preferred data organization. Because our directory structure evolved from a large longitudinal study with cross-sectional and

longitudinal analyses, we organize multiple visits for each subject as subdirectories under that subject's main data directory. However, this organization is highly inconvenient for processing with `make`.

These basic issues and the goal of minimizing complexity drive the directory structure described in this practical.

Practical Example: A More Realistic Longitudinal Directory Structure

Organizing Longitudinal Data

Follow along with this example. Copy directory
`$MAKEPIPELINES/oasis-longitudinal-sample-small/`
to your home directory using the following command:

```
$ cp -r $MAKEPIPELINES/oasis-longitudinal-sample-small/
```

This is a very small subset of the OASIS data set (`http://www.oasis-brains.org`), which consists of a longitudinal sample of structural images. There are several T1 images taken at each scan session, and several visits that occurred a year or more apart. I have reduced the size of this directory by taking only one of the T1 images for each person, for each visit, and only of a small sample of subjects.

Look at the directory structure of `subjects/`. A useful command to do this is called `find`. For example, if you are in the `subjects` directory you can type:

```
$ find .
```

You can see that as we have discussed, each subject has one to five separate sessions. The data for each session (here, only a single MPRAGE) is stored under each session directory. I realize that creating a directory to store what is right now a single scan seems a bit like overkill, but in a real study there would be several types of scans in each session directory. Here, to focus on the directory organization and how to call make recursively, we are only looking at one type of scan.

Normally there are two types of processing that are performed for a study. The first are subject-level analyses — in short, analyses that are completely independent of all the other subjects. The second are group-level analyses, or analyses that involve all the subjects data, or a subset of the subjects. In general, a good rule of thumb is that the results of subject-level analyses are best placed within the subject directories.

Group-level analyses seem to be best found elsewhere in the project directory, either as a subdirectory within a specific timepoint or organized at the top level.

Change into the directory `visit1/` within your copy of `oasis-longitudinal-sample-small`:

```
$ cd visit1
```

What we want to do is create the symbolic links for each subject's first visit here. You can do one link by hand:

```
$ ln -s ../subjects/OAS2_0001/visit1 OAS2_0001
```

This command creates a symbolic link called `OAS2_0001` to the original directory `../subjects/OAS2_0001/visit1`. Now, if you enter `ls -l` into the command line while remaining in the `visit1` directory, you will notice that the subdirectory `OAS2_0001` is symbolically linked, as indicated by an arrow, to the original target directory. You can also check whether a file or directory is linked by using the `ls -F` shorthand command, which indicates symbolic links with an symbol. The command `ls -L` dereferences the symbolic links so that you can view information for the original target file or directory itself.

! Your `ls` command may be aliased to something pleasing, in which case you might see slightly different behavior than described here.

You can also create symbolic links in bulk. To do so, remove the link that you have just created and use the program in the `visit1` directory (`makelinks`) to create all the subject links for `visit1`.

Take a look at the `makelinks` script in Figure 2.1. This script loops through all of the subject directories that have a first visit (visit1). It extracts the subject identifier from the directory name (`$i`) by using the `egrep` command to find the bit of the name that matches the subject identifier pattern (`OAS2_[0-9]`). With this information, it can link the subject name to the directory.

! The current directory won't be in your PATH, so make sure to call it with `./makelinks`.

```
#!/bin/bash
for i in ../subjects/*/visit1
do
        subject=`echo $i| egrep -o 'OAS2_[0-9]*'`
        ln -s $i $subject
done
```

Figure 2.1: Script to create symbolic links within a longitudinal directory structure

Recursive make

Now let's look at the Makefile (`visit1/Makefile`). This is just a top-level "driver" makefile for each of the subjects. All it does is create a symbolic link, if necessary, to the subject-level makefile, and then it goes in and calls `make` in every subdirectory.

Do you know why the subject target is a phony target? The reason is that we want `make` to be triggered within the subject directory every time we call it.

Create the symbolic links to the subject-level makefile as follows:

```
$ make Makefile
```

Do you know why we use the `$PWD` variable to create the link? If we used a relative path to the target file, what would happen when we go to the subject directory?

Let us see how this works. Look at the subject-level makefile (`Makefile.subdir`). Go into a subject directory and run `make`.

By now, you might be getting really tired of seeing the same `fslreorient2std` and `bet` commands. `make`, by default, will echo the commands that it executes to the terminal. If you would like it to stop doing that, you can tell it not to do that by prepending the `@` character to each line.

```
%_T1_skstrip.nii.gz: %_T1.nii.gz
	@bet $< $@
```

Now go find the same subject via the subject subtree:

```
$ cd  /oasis-longitudinal-sample-small/subjects/visit1/OAS2_0001/visit1
```

Type `make` and see that it works. Part of this magic is that we set the subject variable correctly, even though where it appears in a directory path is different in each place.

There is a useful little rule defined in the GNU Make Book (Chapter 2, p. 44) that may be useful for checking that you have set variables correctly. Add the following lines to the subject-level makefile.

```
print-%:
	@echo $* = $($*)
```

Now if you type:

```
$ make print-subject
```

You can see the value that the variable `subject` is set to. Note that even if you place this new rule at the very top of the makefile, it will not execute this rule by default.

It will fall through to the next non-pattern rule. This is one of the ways in which pattern rules (implicit rules) differ subtly from explicit rules.

Running `make` over multiple subject directories

Now that we have verified that the individual makefile works, we can go to the `visit1` directory and process all the subjects. First, go back and edit the subject-level makefile to remove the `@` characters in front of the commands so that they are printed.

From within the `visit1/` directory, type:

```
$ make -n TARGET=skstrip
```

Note that what this is doing is (recursively) going into each subject directory and calling `make`. It will do this whether or not there is anything to do within the subject directory, because each subject directory has no dependencies. However, because we have specified the `-n` flag, it prints out what commands it will perform without actually executing them.

We can do this work in parallel. Bring up a system monitor (`gkrellm` is installed on the IBIC systems). See how many processing units you have and how busy they are. Note these numbers and get familiar with the configuration of the computers that you have in the lab. In general, each job will require a certain amount of memory and can use at maximum one CPU. A safe calculation for most things is that you can normally run as many jobs at one time on a computer as you have CPUs. This is a huge oversimplification but it will suffice for now.

You can specify to make how many CPUs to use. For example, if we specify the `-j` flag with an argument of 2 (processors), we can parallelize execution of `make` over two CPUs.

```
$ make -j 2 TARGET=skstrip
```

If you specify `make -j` without any options, it will use as many CPUS as you have on your machine. This is great if all the work you have "fits" into the computing resources that are available. However, if it does not, you can use a computing cluster.

In our environment, we use the Sun Grid Engine (SGE) to distribute jobs across machines that are "clustered" together. To run on a cluster, you need to be logged on one of the machines that is allowed to submit to the grid engine.[1] Once there, you can use the command:

[1]In IBIC, they are: `broca`, `dentate`, `homunculus`, `pole`, `pons`, `precuneus`, and `triangularis`.

```
$ qmake -cwd -V - -j 20 TARGET=skstrip
```

Here, we use `qmake` instead of `make` to interact with the grid engine. The `-cwd` flag says to run from the current working directory, and `-V` says to pass along all your environment variables. You will normally want to specify both of these flags. The `-` specifies to `qmake` that we are done specifying `qmake` flags and are now giving normal flags to `make`. For example, in this command we specify 20 jobs.

As an optional exercise, here you might want to set up the same directory structure for `visit2/` and build everything from scratch in parallel.

Running FreeSurfer

Now let us look at an example of a subject-level analysis that we typically don't run within the subject directories. FreeSurfer (see Desikan et al., 2006; Fischl et al., 2004b,a) is a program that we use for cortical and subcortical parcellation. It itself is a complicated neuroimaging pipeline that can use `make` to drive it. It likes to put all subject directories for an analysis in one directory, which makes it difficult to enforce the subject-level structure described. But in general, it is much wiser to work around what the programs want to do than to reorganize their output in ways that might break when the software is updated! This is our approach.

The FreeSurfer example is documented in Testsubject FreeSurfer.

Practical 3

Getting Down and Dirty with `make`

Learning objectives: Understand how to write a simple makefile for a single subject pipeline.

Practical Example: Running QMON

As we mentioned in the previous practical, we can determine how many processing units we have on our computer by running the system monitor. To do so, type `gkrellm` into the command line. As you will see, this software also allows us to monitor the status of CPUs, memory usage, and disk space.

If the number of jobs that you need to run exceeds the number of cores that you have, submitting jobs to the grid engine (explained in Chapter 2) might be a good idea. But how do we check whether the machines in the cluster we are interested in are available for batch processing? The `qmon` monitor is a graphical user interface that comes with the Sun Grid Engine software. It gives us the ability to track the status of machines in a cluster (and more besides, but you do not have to worry about that for now).

! You may have a different system monitor installed at your site. Ask your system administrator.

To run `qmon`, you need to be logged in to one of the cluster workstations. This can be done by initiating a secure shell session, as follows:

! A list of the clusters and machines available for use at IBIC can be found on the wiki.

```
$ ssh -X <username>@broca.ibic.washington.edu
```

If you do not specify the `-X` flag, you will be able to log in to the remote machine, but graphical output (such as from `fslview` and `emacs`) will not show up on your graphical display. The `-X` flag forwards this output to the computer that is managing your graphical display.

Once you have entered your password at the prompt, type `qmon` into the command line. Of interest to us is the first button in the panel: "Job Control." When you click on this, you will be able to see the number of jobs running on the cluster and the number of jobs in the queue, along with the username of the person who submitted those jobs. Ideally, you want to submit your jobs to the cluster when the cluster is free.

However, this scenario does not necessarily always present itself. If you use `qmake`, you will get an error by default if the grid engine is busy. You can get around this by using the `-now no` options to `qmake`, which will cause it to wait until the grid engine is free. For more information on how to use the grid engine to run a parallel `make` workflow, refer to the manual, or Practical 2 where we covered the use of `qmake`.

If you are primarily employing FSL tools with `make`, you can use `FSLPARALLEL` to submit jobs for parallel processing. Before running `make`, set up the following environment variable in your shell:

```
$ export FSLPARALLEL=true
```

Once you have done this, you may go ahead and use `make` *without parallelization* to submit jobs to the grid engine. This calls a script called `fsl_sub` that FSL uses to schedule and submit jobs in parallel in the way that some of its cluster aware tools, such as MELODIC, are capable of doing. Jobs parallelized using `FSLPARALLEL` will automatically be queued under "Pending Jobs" if the cluster workstations are busy processing other jobs.

Practical Example: A Test Subject

If you have not already, copy `testsubject` to your home directory as follows:

```
$ cp -r $MAKEPIPELINES/testsubject ~
```

This copy will take a few minutes, depending on what kind of file systems you are copying from and to, because this directory is fairly large.

Go into this directory and look at its contents.

```
$ cd testsubject; ls
```

You will see four subdirectories: `bin/`, `lib/`, `test001/` and `freesurfer`. As I am sure you have noticed, this is a much simplified structure as compared to the longitudinal directory structure that we looked at in the previous practical. For this

example, I have eliminated the individual subject subdirectories (because there is only one) and the symbolic links (because there is only one timepoint). This will allow us to focus on the makefile structure for multiple types of analyses.

While there is no strict convention dictating the manner in which you should organize your project directory, it is good practice to set up a directory tree structure that clearly separates the various parts of your projects from one another. In most cases, a `bin/` directory stores executables and scripts that have been written for the project. The `lib/` directory, on the other hand, typically holds makefiles used for the project (although in some IBIC project directories, a `templates/` directory is used for this purpose instead). The `testsubject/` directory in this example stores a single subject's processed data.

Now, `cd` into the `test001/` directory and have a look at the top-level makefile. In the first section of the makefile (prior to the listing of the various targets and dependencies), I have set two conditionals to check whether we have a FLAIR and T1 image. While this may not useful when we are dealing with a single subject, you will find that it makes processing a large number of subjects much easier. What the two conditionals are doing is that they are asking shell to go into a subject directory and look for files called '`00_NOFLAIR`' and '`00_NOT1`'. For this to work, you will need to create these files beforehand in the subject directories of subjects whom you know do not have these images. This is relatively easy to do and makes processing less of a pain. It will also help you to debug `make`, which is likely to break when it finds that it is missing dependencies that do not exist.

If a file '`00_NOT1`' is found, a FALSE boolean is returned, and `make` will not make targets that require a T1 image as a dependency. This is specified in the next conditional:

```
❶ ifeq ($(HAVET1),true)
all: $(call print-help,all,Do skull stripping, etiv, HC volume
   calculation) T1_skstrip.nii.gz first etiv
❷ else
all:  $(call print-help,all,Do skull stripping, etiv, HC volume
    calculation)
        @echo "Subject␣is␣missing␣T1␣-␣nothing␣to␣do␣here."
endif
```

❶ If the variable HAVET1 returns TRUE, `make` will make the targets listed in `all`.

❷ If the variable HAVE1 returns FALSE, `make` will echo to your screen that the subject is missing a T1 image.

Test out this conditional by making a `00_NOT1` file in the `testsubject` directory like so:

```
$ touch 00_NOT1; make -n
```

You will find that `make` will tell you that the subject is missing a T1 image and that there is nothing to do.

Remove `00_NOT1` and try running `make` again. You can see that `make` is now running our edited version of the first script, to conform to the Enigma protocol (see Stein et al., 2012). The protocol may be viewed online.

Because running FIRST will take a little time, start it now and place it in the background:

```
$ make first &
```

Estimated total intracranial volume

Let's now turn our attention to the calculation of estimated total intracranial volume (etiv). This can be estimated by running FreeSurfer, but FreeSurfer takes a long time to run and had some difficulties with automatic processing of many of the subjects in the study that this makefile was modified from.

One technique for estimating the total intracranial volume is simply to linearly register the brain to standard space (see http://enigma.ini.usc.edu). The transformation matrix describes the scaling that is necessary to align the brain to the standard brain. The inverse of the determinant of this transformation matrix is a scaling factor that can be multiplied by the size of the template (e.g., the volume of the standard space brain) to obtain an actual volume.

If you type `make -n etiv`, the first command that will be executed is to skull-strip the brain. This uses the `-B` flag, which does bias field correction and is rather slow. However, you can see that the `T1_skstrip` file is actually sitting in that directory. So why does it want to recreate it?

Look at the dates at which the files in your `testsubject/` directory were last modified. Now look at the dates in the master directory `project_space`. Copying, using `cp -r`, did not preserve the dates! Everything got the new date that it was created. Up until now, we have created everything in the directories in which we are working, from scratch.

You can avoid this by copying the files in some way that preserves the dates. For example, you can type:

```
$ cp -r -preserve=time-stamps
```

Alternatively, you can use `tar` to create an archive, copy the archive, and unarchive it. For example, to tar up a directory called `mydirectory` into an archive called

archive.tar in your home directory, type:

```
$ tar cvf ~/archive.tar mydirectory
```

To untar this archive that you have just created, type:

```
$ tar xvf ~/archive.tar
```

How can we "trick" make into not recreating this skull-stripped file? An easy way is simply to touch the skull-stripped file, like so:

```
$ touch T1_skstrip.nii.gz
```

Once you have done this, go ahead and make etiv:

```
$ make etiv
```

This time, make will not try to recreate the skull-stripped file, because touching it makes it newer than the image from which it is created. Instead, it will run FLIRT on the T1 skull-stripped image. FLIRT is a FSL tool for linear registration, and is used to align a brain to standard space by transforming the skull-stripped brain image using an affine matrix. Following this, make will extract the intracranial volme from the output matrix generated by FLIRT and print this to a comma-separated value (csv) file.

To look at the contents of the file eTIV.csv, type:

```
$ cat eTIV.csv
```

Often, there are statistics that need to be collected for each subject. One way to go about this is to write a program that gathers the correct statistics for each individual, and then decide whether or not you want to call the program from within make. I find, however, that often I want to know what those numbers are while I am examining the output for a single individual, such as now. Thus, I normally create a little comma separated value (csv) file that contains the subject ID and whatever statistics I am interested in looking at. This way, I do not need to remember the commands to obtain the statistics I want (e.g. what NIfTI file should I be looking at? What command do I need to use to extract the right value?). This will also allow me to go to the "top level" directory that contains all the subjects within it and use cat to gather all the files into a single file for data analysis later on.

As it is, I happen to know that this subject has a big head. And yet, the etiv file tells us that the scaling factor is approximately 0.6. This is, of course, a contrived example. But have a look at the skull-stripped image to see what went wrong. You will see that too much of the brain was removed, which may have occured because there is a lot of neck in the T1 image. As such, you will need to correct the skull-strip.

One way to do this is by specifying the center of the brain:

```
$ bet T1.nii.gz T1_skstrip.nii.gz -c 79,120,156
```

This improves the situation but it is still not great. Moreover, in a large study (suppose you are looking at 1,000 brains), even a modest 10% failure in running a skull-stripping program like FSL's BET would mean having to handle 100 brains by hand.

Look back at the makefile. You can see that there are two alternative rules for obtaining the skull-stripped brain. My favorite way is to obtain a brain mask from FreeSurfer. It takes many hours to run, but if you have run it, why not just use it?

But for now, consider the program `robex`. This is a robust skull stripping program (Iglesias et al., 2011) that we can run.

```
$ make robex
```

Now check to see that the skull-stripping is better. Use whatever skull-strip you think best represents the brain volume (so that the scaling will be improved, at least – even if it is not perfect) and type:

```
$ make etiv
```

You should see that the scaling factor is higher than it was. Note that it is still less than one. This is because the MNI template has "drifted" and is larger than most actual brains.

Hippocampal volumes

By now, you should have a file called `hippo.csv` that contains the hippocampal volumes from the Enigma protocol. However, we have not done the quality checking step that is recommended by the protocol. In this example, we will add the step to create a webpage that displays the registrations to the makefile.

This command is as follows:

```
$ ${FSLDIR}/bin/slicesdir -p ${FSLDIR}/data/standard/MNI152_T1_1mm.nii.gz
*_to_std_sub.nii.gz
```

Run it and you can see the output. Then, delete the directory, `slicesdir`, that you just created. Now, as an optional exercise, create a rule called `firstqa` that will execute this command and make sure that it is part of the target to make `first`.

Implementing a Resting State Pipeline: Converting a Shell Script into a Makefile

In the `testsubject/test001` directory, you will find a bash script called `resting-script`. The goal for this part of the practical is to convert that shell script into a makefile.

A few things that are worth taking note of:

1. Note that the shell script assumes it will never be re-run again and simply calls `mkdir xfm_dir`. If this directory already exists, however, you will get an error. To avoid this, add a `-p` flag to the command `mkdir`. This allows it to create the specified directory if it does not exist, but to not give an error otherwise. This is especially useful in makefiles that create deep directory structures.
2. Many FSL commands (and other neuroimaging commands) automatically add file extensions such as `.nii.gz` to the input files if you do not append them. However, a makefile will not do this. Therefore, when converting commands from a script, you need to make sure that you don't take such shortcuts in a makefile. A good rule of thumb is to specify all extensions in your targets.
3. The decision whether to make something a multi-line recipe, a shell script of its own, or a short recipe is a little arbitrary. If I think that I want to do something often on a command line, I make it into a short shell script and call it from a makefile. In this example, despiking with AFNI is short enough that it can fit into a recipe.
4. However, anything complicated that constitutes a pipeline in itself is better coded from the beginning as a makefile. Not only does this allow parallelism and fault tolerance at a fine granularity, but you can see that it is easy to ask someone for commands to do registrations or obtain a skull-strip from FreeSurfer. Makefile commands can easily be shared, updated, and replaced with the latest methods. Instead of using FSL FLIRT for registration, try editing your makefile such that registration is done with `epi_reg`.
5. Note that `3dDespike` is an AFNI program that has been compiled to use all the cores in a machine. You will want to turn that off because you will do the

parallelization yourself; this can be achieved by entering the following line in a makefile: `export OMP_NUM_THREADS=1`

To go about translating this script into a makefile, it may help to do things one step at a time. I recommend that you write and *test* one rule at a time for each step in the workflow in the command line. This will help with debugging. Create a rule, test it with the `-n` flag (which echoes to your shell what `make` intends to do to make the target), then run `make` without the flag to create your specific target. Then delete all the things that you have created and add the next rule. This will allow you to see if things chain together. One of the worst things you can do (if you are not very comfortable with `make`) is to write your entire makefile and then try to figure out why it does not work as expected.

> **!** To create a makefile in emacs, you need to ensure that you are in "`make`" mode. Type `esc x makefile_gmake_mod` into the emacs console to change the mode you are operating in.

For the purposes of this exercise, create a makefile named `Makefile.rest` in order to distinguish it from the top-level makefile that already exists.

To ensure that you are calling the right makefile in the command line, you should do the following:

```
$ make -f Makefile.rest nameoftarget
```

The `-f` flag lets you specify the name of your makefile where the rule to creating your target of interest exists. Recall that by default `make` will look for commands in files called `Makefile` or `makefile`. Because `Makefile.rest` has a different name, you need to tell `make` about it.

Now, let us have a look at the first part of `resting-script`.

If you need help with identifying your target and dependency from the shell commands alone, you should look at the usage of the command in your terminal window. E.g. for FLIRT, simply type `flirt`. You will be able to understand what the flags mean, and from there find out what your input and output is.

The script from Figure 3.1 would look like the rules below in a makefile:

```
1  PROJECT_HOME=/mnt/home/username/testsubject/testsubject
2  SHELL=/bin/bash
```

1 When creating your makefile, you must specify your project home.
2 Be sure to tell `make` to use `bash` by setting the default shell.

```
3  .PHONY = all clean

4  all: xfm_dir/T1_to_MNI.mat rest_mc

5  xfm_dir/T1_to_MNI.mat: T1_skstrip.nii.gz
           mkdir -p xfm_dir ;\
```

```
#!/bin/bash

mkdir xfm_dir

#Create transformation matrices

#Making the 2mm MNI -> ANAT matrix
echo "Registering the T1 brain to MNI"
flirt -in T1_skstrip.nii.gz -ref /usr/share/fsl/data/standard/MNI152_-
T1_2mm_brain.nii.gz -omat xfm_dir/T1_to_MNI.mat
echo "Preprocessing RESTING scans..."

### MOTION CORRECTION
echo "Begin motion correction for rest.nii.gz"
mcflirt -plots rest_mc.par -in rest.nii.gz -out rest_mc -rmsrel
-rmsabs
```

Figure 3.1: `resting-script` file

```
        flirt -in T1_skstrip.nii.gz -ref /usr/share/fsl/data/
           standard/MNI152_T1_2mm_brain.nii.gz -omat xfm_dir/
           T1_to_MNI.mat

6 rest_mc.nii.gz: rest.nii.gz
        mcflirt -plots rest_mc.par -in rest.nii.gz -out rest_mc
            -rmsrel -rmsabs
```

3 Define your phony variable which is a special target used to tell `make` which targets are not real files. `clean` is a common rule to remove unnecessary files, while `all` is a common rule to make all your other targets.

4 Define `all` to include all your targets.

5 Format your commands to take the form of "rules" to include a target and dependency. Every command following your [target:dependency] definition should be entered on a new line beginning with a TAB character. The target is your output and the dependency is your input (you can, however, have several targets and dependencies listed under a single rule). Remember to also use `;\` at the end of each line to tell `make` to read each line separately, as in the example for creating transformation matrices. Here, we are using FSL FLIRT to create an output matrix called `T1_to_MNI.mat`.

6 For motion correction, our expected output (i.e. target) is `rest_mc.nii.gz`,

and our input (i.e. dependency) is `rest.nii.gz`. The last line tells `make` to run FSL `mcflirt` on `rest.nii.gz`.

Practical 4

Advanced Topics & Quality Assurance with R Markdown

Learning objectives: Understand how to write self-documenting makefiles in an organized directory structure, use macros, and write quality assurance reports using R markdown.

Creating a `make` help system

It is sometimes useful to know what a makefile does without having to look through the entire makefile itself. One way of getting that information at a glance is to create a `make` help system that prints out the targets and a short summary explaining what each target does.

To go about creating this help system, you need to create a separate "help" makefile that tells `make` what to do when you call for help. Before we expand on that, however, let us take a look at the main makefile in the `testsubject` directory copied over from `/project_space/`.

```
$ cd  /testsubject/test001
```

Let the variable $(PROJECT_HOME) represent the pathway to your `testsubject` directory. You can set this variable in top-level makefile.

```
PROJECT_HOME=/yourhomedirectory/testsubject
```

Open up `Makefile` in an editor. You will notice that one of first few lines at the top of the file asks `make` to include a makefile called `help_system.mk`; this is the "help" makefile that we alluded to earlier. The `include` directive tells `make` to read

other makefiles before continuing with the execution of other things in the current makefile.

As you scroll down, you will see that some of the targets are followed by a `call` command before their dependencies. For instance, look at the line:

```
robex: $(call print-help,robex,Alternate skull stripping with
   ROBEX) T1.nii.gz
```

This tells `make` to return the target `robex` along with its description when `print-help` is called (`print-help` is defined in `help_system.mk`, as we will see later). Other targets such as `freesurferskstrip` and `etiv` also make calls to `print-help`.

Now, have a look at `$(PROJECT_HOME)/lib/makefiles/help_system.mk`.

```
help: ; @echo $(if $(need-help),,Type `$(MAKE)$(dash-f) help`
   to get help)
```

This line tells `make` to echo the fellowing when you type `make` into the command line.

```
$ make
Type 'make help' to get help
```

Figure 4.1: `make`'s Help System

The variable `need-help` is set such that `make` will filter for the word `help` in your command line entry to decide whether or not you need help. If so, the function `print-help` will be called, and this will result in `make` printing the name of your targets and their descriptions to your shell. Depending on whether or not this will be helpful for you, you may want to copy `help_system.mk` to your own project directory and include it in your top-level Makefile.

We will not reproduce the details of how `help_system.mk` works in this practical. For an excellent description of this simple but useful system, please refer to page 181 of John Graham-Cumming's GNU Make Book, or to his blog posting.

Makefile Directory Structure

Often several people collaborate to work on analyzing different types of data from a project and this calls for several makefiles. Or, you may wish to split up a processing pipeline into various parts as we have in our Makefile example. In the main makefile `Makefile` from the `$(PROJECT_HOME)/test001` directory – and this was mentioned earlier – you should have noticed that several other makefiles were included.

```
include $(PROJECT_HOME)/lib/makefiles/help_system.mk \\
include $(PROJECT_HOME)/lib/makefiles/resting.mk \\
include $(PROJECT_HOME)/lib/makefiles/xfm.mk \\
include $(PROJECT_HOME)/lib/makefiles/fcconnectivity.mk \\
include $(PROJECT_HOME)/lib/makefiles/QA.mk
```

Here, we see that there are makefiles for:

1. defining the `make` help system.
2. processing resting-state data called `resting.mk` (this is documented in detail in Preprocessing Resting State Data).
3. obtaining transformation matrices for registrations called `xfm.mk` (see Testsubject Transformations for documentation).
4. running seed-based connectivity analysis called `fcconnectivity.mk` (see Seed-based Functional Connectivity Analysis I for documentation).
5. creating QA reports called `QA.mk` (see Testsubject QA Makefile for documentation).

For a full description of the makefiles (excluding `help_system.mk`), we encourage you to refer to their full documentation.

Note that makefiles should be stored in the `lib` directory as we did in this example. This is good practice and helps keep your directories organized and less cluttered. The importance of creating a tidy directory tree structure has been continually emphasized throughout this course. Refer to Practical 3 or Chapter 2 of the manual for a more thorough discussion of the directory structure common to most IBIC projects.

The `clean` target

In `make`, the `clean` target normally delete all unnecessary files that were created in the process of running `make`. Remember that storage is limited on IBIC systems! Cleaning up your project directory will make space for future projects that you will be working on. The target itself is usually specified last in a typical `make` recipe, and must be included in your list of `.PHONY` targets if you choose to define it.

Now, go to your `$(PROJECT_HOME)/test001/` directory and open `Makefile` in an editor. Scroll all the way down. You will see both an `archive` and `clean` target. `archive` is a target intended to clean up the directory for archiving after a paper has been accepted. The purpose of this target is to retain important results but remove the partial products. Obviously, what you define as “important results” depend upon the kind of analysis that you are doing and the amount of risk you are willing to assume.

The `clean` target in this makefile includes `clean_qa` and `clean_rest` which are themselves targets in other makefiles in the `lib/` directory. Typically, files that are "easy" to make and are no longer necessary can be removed. Other files, such as those generated by FreeSurfer's `recon-all`, require more compute resources and are not as easily remade. Other examples of files that you may not want to remove include hand-edited files, and end stage products of quantitative processing pipelines. You should therefore think carefully about what you want to remove and what you want to keep.

Creating New Makefile Rules On The Fly

One of the drawbacks of implicit rules in `make` is that they can only substitute one pattern at a time. For example, we may give multiple fMRI task blocks (which could be matched by one pattern) but then wish to process each contrast of parameter estimates (COPE) using an implicit rule. Thankfully, there is a way to "create new makefile rules on the fly" using functions built in to `make`. We will work through an example that overrides default Feat registration and does just this (see Using ANTs Registration with FEAT).

Incorporating R Markdown into `make`

Quality assurance (QA) is an important step in a neuroimaging analyses pipeline. At IBIC, data is typically preprocessed and checked for quality before proceeding with further analyses. This can be done both qualitatively and quantitatively. Some of the common aspects of data that are looked at include motion outliers, quality of brain registration/normalization to a subjects-specific or standard template, and whether brain segmentation has been performed correctly.

While neuroimaging packages usually include QA tools of their own (such as FSL's FEAT report), built-in QA tools have limited application when you are using more than one neuroimaging package to process your data or if you want to view your data in a non-traditional way.

Know that you may certainly create QA measures for each subject in your project and inspect these one-by-one or examine NIfTI images created at various stages with a previewer, but this may be inconvenient and time-consuming. Because there usually are several things that have to be inspected during a quality assurance procedure, it is incredibly helpful to have images and data parameters or statistics displayed on a single page for each subject in a project. Alternatively, you may choose to look at a single aspect of your data and would like to concatenate a QA measure from all of your subjects into one report. This is where R Markdown comes in.

R Markdown is an authoring format that allows us to generate reports using a simple markup language (http://rmarkdown.rstudio.com). It is extremely versatile in that it can incorporate code from multiple languages (including HTML, `bash`, `python` and `R`) to generate HTML and PDF documents, or even interactive web applications.

Although QA reports be viewed using PDF documents or HTML pages, at IBIC we prefer to use the HTML format for a couple of reasons. For one, a HTML report gives us the ability to look at moving GIF images, which are useful when we want to view the collected brain scans as a time series. In addition, it circumvents the issue of pagination and allows us scroll seamlessly through all our images and statistics in a single page. PDFs, tend to be more cumbersome to use as images may be awkwardly split between pages and cause page breaks if they are not properly sized.

Go to the directory `$(PROJECT_HOME)/freesurfer/QA` and open up `QA_check.html` with your internet browser.

```
$ iceweasel QA_check.html
```

Clicking on the link `testsubject` will redirect you to a page that holds the images generated by FreeSurfer. Here, you can see the segmented and parcellated brain of the subject, along with images showing the surface curvature of the brain. This page is automatically created by FreeSurfer's recon-all.

We can also create our own QA images to, say, check whether a T1 brain has been properly registered to a brain in standard space. These images can be specified as targets in your makefile as follows.

```
QA/images/T1_to_mni_deformed.png: xfm_dir/T1_to_mni_deformed.
    nii.gz $(FSLpath)/data/standard/MNI152_T1_2mm_brain.nii.gz
	mkdir -p QA/images ;\
	$(FSLpath)/bin/slicer $(word 1,$^) $(word 2,$^) -s 2 -x
	     0.35 sla.png -y 0.35 slb.png -z 0.35 slc.png ;\
	pngappend sla.png + slb.png + slc.png QA/images/
	    intermediate1.png ;\
	$(FSLpath)/bin/slicer $(word 2,$^) $(word 1,$^) -s 2 -x
	     0.35 sld.png -y 0.35 sle.png -z 0.35 slf.png ;\
	pngappend sld.png + sle.png + slf.png QA/images/
	    intermediate2.png ;\
	pngappend QA/images/intermediate1.png - QA/images/
	    intermediate2.png $@ ;\
	rm -f sl?.png QA/images/intermediate?.png
```

Here, FSL `slicer` is used to create 2D images from 3D images. To get a better understanding of how slicer is used, simply type `slicer` into the command line. This FSL page also provides a brief description of what slicer does.

In the context of the above example, slicer first overlays the T1_to_mni_-deformed image on top of the MNI152 brain. Subsequently, it creates several 2D images named sl?.png that come from sagittal, coronal or axial slices. This is indicated by the -x/y/z flags used followed by the name of the image generated. Note that these files are temporary! Once pngappend has been used to paste these images next to each other to generate a single image, the temporary files are removed. If you are using FSL slicer for more than one instance in a makefile, the temporary files created may cause make to crash if they are given the same names and are put into the same directory when make is run in parallel. Make sure that the names of your outputs from FSL slicer do not overlap each other.

Given that the code used to generate QA images can be very messy and ugly, you may want to create a bash script to create those images and call that from your QA makefile instead. This also avoids potential problems that may arise when many similarly named temporary files are generated that may cause make to continually overwrite these files when using a tool like FSL slicer in parallel. A wonderful example of such a script can be found at $MAKEPIPELINES/tapping/bin/sliceappend.sh (credit goes to Matt Peverill!).

Once we have created all the QA images we want to include in our HTML report, we need to create a .Rmd file that tells R Markdown where to find these images.

Now let us have a look at a subsection of a .Rmd file on the next page. The file is $(PROJECT_HOME)/lib/Rmd/fMRI.Rmd.

A .Rmd file should always begin with a demarcated section listing the title of the report and the type of file to be created. We see that the output here is a HTML document. toc refers to table of contents.

In R Markdown, the size of headers depend on the number of #(hash) symbols used before the name of a header. This is akin to how the titles of major sections of a text are rendered in the largest text size, and subsections along with sub-subsections are printed in smaller-size text. A double hash symbol before a title will mean that the title will be treated as a "sub-section" and be printed in smaller size than the main headers. As the number of hash symbols used before a title increases, the text becomes smaller.

Notice that HTML syntax is used to insert images into the report. Heights and widths of images can be specified as well.

Assuming we want to print out some information about the subject into the report, we can use bash within a R Markdown file to grep for the stuff we need or cat a value from a file. In the example above, we open the bash script portion of the file with three backticks and a curly bracket telling R that the following text is written in bash code. Here, we are asking bash to cat the values from two text files that gives us the mean absolute and mean relative displacement in mm (see cat help if you are not familiar with its usage). These will then be printed out into our HTML report.

```
--
title: Quality Assurance of Preprocessing for TASK for ID SUBJECT
output:
html_document:
keep_md: no
toc: yes
force_captions: TRUE
--
#T1 Skull-Strip

<img src="/project_space/makepipelines/testsubject/test001/QA/images/T1_-
skstrip.gif" width="500px" />

#Parameters

##Time Series Difference Analysis

<img src="/project_space/makepipelines/testsubject/test001/QA/images/TASK_-
tsdiffana.gif" width="750px" />

# MOTION

##Motion Statistics

```{r engine='bash', echo=FALSE}
val=`cat /project_space/makepipelines/testsubject/test001/rest_-
dir/TASK_mean_abs_disp.txt`
echo "Mean Absolute Displacement : $val mm"
val=`cat /project_space/makepipelines/testsubject/test001/rest_-
dir/TASK_mean_rel_disp.txt`
echo "Mean Relative Displacement : $val mm"
```
```

Figure 4.2: Example of a R Markdown File

Once we have a .Rmd file ready, we want to tell make to create the HTML report for us. By doing so, we can parallelize the generation of QA reports. We do this by inserting a target such as the following into our QA makefile (which is fully

documented in Testsubject QA Makefile):

```
QA/rest_Preprocessing.html:  (PROJECT_HOME)/lib/R/fMRI.Rmd TSNR
    MotionGraphs SkullstripQA
        sed -e ‘s/SUBJECT/$(subject)/g’ -e ‘s/TASK/rest/g’  $(
           word 1,$^) > QA/rest_Preprocessing.Rmd ;\
        R -e ‘library(‘‘rmarkdown’’);rmarkdown::render(‘‘QA/
           rest_Preprocessing.Rmd’’)’
```

The target is your HTML file, and the dependencies are your `.Rmd` file and your images or whatever else you decide to include in your report. The output is dependent on the `.Rmd` file to trigger to regenerate when the source has been changed. We then use `sed` to replace instances of the string “SUBJECT” in the R Markdown file with the actual subject ID. The subject ID variable `$subject` should have been set at the initial portion of your makefile.

`make` will then call R to load the package “rmarkdown” so that it can read R Markdown, and proceed to generate/render your report, which will look something like Figure 4.3.

Holy Bad Skullstripping! This is one of the many reasons why we do QA. The full report can be viewed in your favorite Internet browser and can be found at `$(PROJECT_HOME)/test001/QA/rest_Preprocessing_example.html`.

And that’s it!

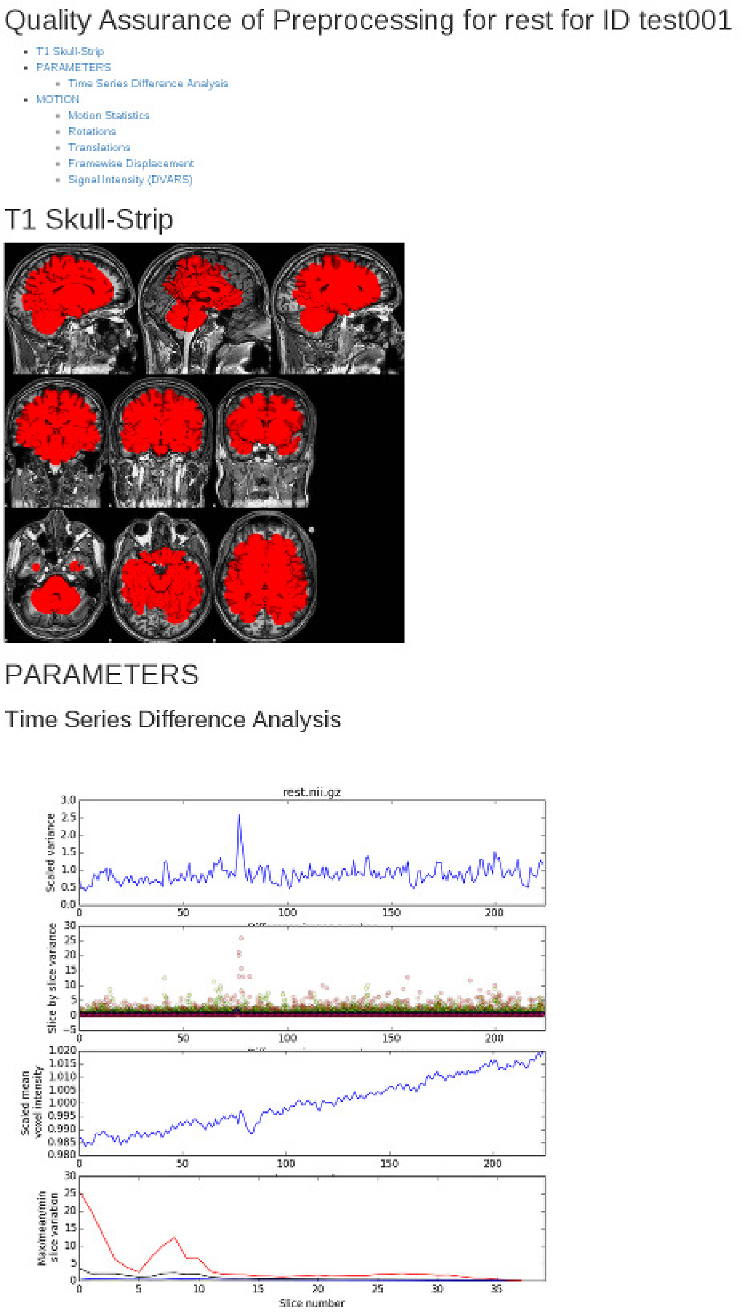

Figure 4.3: QA report in R Markdown

Part III

Examples

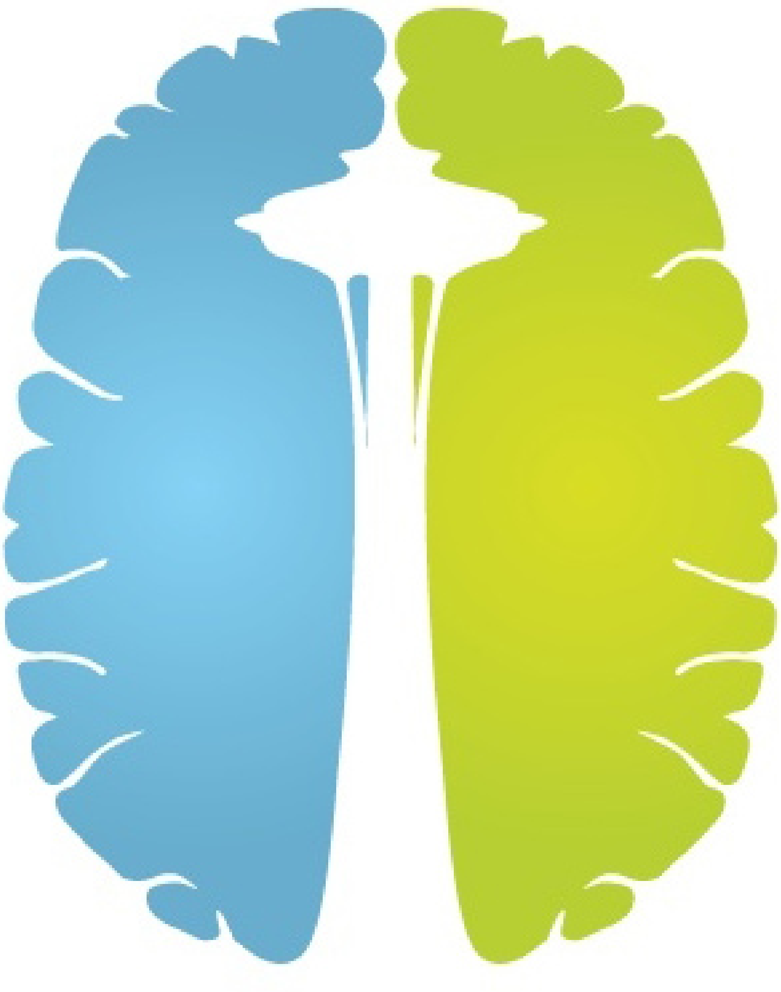

This part includes examples of Makefiles included in the practical data. We assume that you have unzipped the practical data into a directory somewhere in your local environment. Once you have done that, you should set an environment variable called `MAKEPIPELINES` to refer to this directory. In the command below we assume you have placed it in your home directory:

```
$ export MAKEPIPELINES=~/makepipelines
```

The examples included below will reference this environment variable as necessary to find the correct files.

Please contact the example's author with questions, or Katie Askren or Tara Madhyastha where no specific author is listed.

Example 1

Downloading Data From XNAT

Karl Woelfer[1]

This is an example of how to use a Makefile to create and populate a project directory with images from an open dataset stored in an XNAT (eXtensible Neuroimaging Archive Toolkit) database, in this case from the NITRC (Neuroimaging Informatics Tools and Resources Clearinghouse) 1000 Functional Connectomes project image repository at `http://www.nitrc.org/ir`. The code for this example is located at `fcon_1000/Makefile`.

To run this pipeline you will need to have first created an individual user account on NITRC, at `http://www.nitrc.org/account/register.php`, and obtained access to the 1000 Functional Connectome project.

To simplify this example, we download only a subset of the subjects in the repository. A file with the names of these subjects is used to determine what files to download.

In our example, we selected 23 Baltimore subjects, saved in the file `Subjects_Baltimore`. To recreate this file you can do the following. From the NITRC Image Repository home page “Search,” choose Projects → 1000 Functional Connectomes. At the 1000 Functional Connectomes project home page, select Options → Spreadsheet to download a CSV file of the 1288 subjects `AnnArbor_sub00306 ...Taipei_sub91183`. Select a subset, or all, of the subjects from the first column of the downloaded spreadsheet, and save to a text file.

The directory hierarchy that this Makefile creates under your project home, from which `make` is run, is shown in Figure 1.1. Note that this Makefile assumes that the directory `visit1` already exists.

```
  # Site hosting XNAT
1 NITRC=http://www.nitrc.org/ir

2 SHELL=/bin/bash

  # Obtain the list of subjects to retrieve from NITRC
3 SUBJECTS = $(shell cat Subjects_Baltimore)

4 .PHONY: clean all allT1 allT1_brain allrest allsymlinks
```

This portion of the Makefile defines key variables and targets.

1 We set the “base name” of the XNAT web site in a variable `NITRC`. This can be changed when using another XNAT repository, and the variable can be named accordingly.

2 By default, `make` uses `/bin/sh` to interpret recipes. Sometimes this can cause confusion, because `sh` has only a subset of the functionality of `bash`. We set the `make` variable `SHELL` explicitly.

3 The `SUBJECTS` variable will contain a list of the subject data we wish to download. The individual subject names will be used to create directory names.

4 We define six targets that do not correspond to files, so these are denoted as phony targets.

```
5 all: sessionid allT1 allT1_brain allrest allsymlinks
6 allT1: $(SUBJECTS:%=subjects/%/visit1/T1.nii.gz)
```

[1] kwoelfer@uw.edu

```
fcon_1000/
├── subjects/
│   ├── Baltimore_sub17017/
│   │   └── visit1/
│   │       ├── rest.nii.gz
│   │       ├── T1_brain.nii.gz
│   │       └── T1.nii.gz
│   ├── Baltimore_sub19738/
│   │   └── visit1/
│   │       ├── rest.nii.gz
│   │       ├── T1_brain.nii.gz
│   │       └── T1.nii.gz
│   └── ...
└── visit1/
    ├── Baltimore_sub17017/ ..................... A symlink to ../subjects/Baltimore_sub17017/visit1
    ├── Baltimore_sub19738/
    └── ...
```

Figure 1.1: XNAT access directory structure.

```
7  allT1_brain: $(SUBJECTS:%=subjects/%/visit1/T1_brain.nii.gz)
8  allrest: $(SUBJECTS:%=subjects/%/visit1/rest.nii.gz)
9  allsymlinks: $(SUBJECTS:%=visit1/%)
```

5 `all` is the default target, and simply defines the five dependencies.

6 We need to derive the names of all the files that we intend to create from the list of subjects. We do this using pattern substitution to define several targets. Here, we use pattern matching to generate a list of the T1 image names that we need to create. Each of the subject names, e.g. `Baltimore_sub17017`, is used to create the corresponding T1 image (e.g. `subjects/Baltimore_sub17017/visit1/T1.nii.gz`).

7 As above, we form the names of the skull stripped T1 images.

8 And the resting state images.

9 The last thing the Makefile does is create a `visit1/` directory after the `subjects/` directory has been populated. Here we use pattern matching, as above, to generate the list of symbolic links that need to be created. Each `visit1/SUBJECT` directory will be a symbolic link to the actual `subjects/SUBJECTS/visit1/` directory.

```
10 sessionid:
        @echo -n "Username: " ;\
        read username ;\
        curl --user $$username $(NITRC)/REST/JSESSION > $@
```

10 Here we use the client URL Request Library (`cURL`) to create a session with the XNAT server. The first line prompts for the user's name on the XNAT server, the second line reads and stores that in the variable `username`. With one single REST transaction, the `cURL` call on the following line, we authenticate with the XNAT server, entering a password only once, and saving the return value `SESSIONID` in a file named `sessionid`. This single session will persist for an hour. Obtaining a session identifier is important to reduce load on the remote XNAT server.

```
11 subjects/%/visit1/T1.nii.gz:  | sessionid
12      mkdir -p `dirname $@`; \
13      curl --cookie JSESSIONID=`cat sessionid` $(NITRC)/data/projects/fcon_1000/
    subjects/$*/experiments/$*/scans/ALL/resources/NIfTI/files/
    scan_mprage_anonymized.nii.gz > $@
```

11 This recipe downloads all of the T1 images required by target `allT1` (**6**). It has an order-only dependency upon the file `sessionid` because we assume that if these files exist, it does not matter if they are older than the `sessionid` file. They will only be recreated if they do not exist.

12 This command creates the directory `subjects/SUBJECT/visit1` if it does not exist (where SUBJECT is the actual subject identifier).

13 This `curl` command uses the `SESSIONID` which was stored in the `sessionid` file. The URL defined here is specific to the location where scan data of interest is stored on the NITRC instance of XNAT. Note that `$*` is used in two places to refer to the subject identifer, denoted by `%` in the target. The XNAT file `scan_mprage_anonymized.nii.gz` is downloaded and saved under the local name `T1.nii.gz`.

```
$subjects/%/visit1/T1_brain.nii.gz:  | sessionid
        mkdir -p ‘dirname $@‘; \
        curl --cookie JSESSIONID=‘cat sessionid‘ (NITRC)/data/projects/fcon_1000/
           subjects/$*/experiments/$*/scans/ALL/resources/NIfTI/files/
           scan_mprage_skullstripped.nii.gz > $@
```

This recipe is analogous to the previous one, except that it creates the skull stripped T1 images needed by target `allT1_brain`.

```
subjects/%/visit1/rest.nii.gz:  | sessionid
        mkdir -p ‘dirname $@‘; \
        curl --cookie JSESSIONID=‘cat sessionid‘ $(NITRC)/data/projects/fcon_1000/
           subjects/$*/experiments/$*/scans/ALL/resources/NIfTI/files/scan_rest.
           nii.gz > $@
```

Similarly, this recipe creates the resting state images needed by `allrest`.

```
visit1/%:
        ln -s ../subjects/$*/visit1 $@
```

This recipe populates the project top-level `visit1/` directory with symbolic links, pointers to the actual locations of the subjects' `visit1` data downloaded above. This enables an alternate way to access the subject data.

```
clean:
        rm -rf subjects; \
        rm -rf visit1/*; \
        rm -f sessionid
```

This `clean` recipe will delete everything in `subjects/`, links in the `visit1/` directory, and the `sessionid` file.

Example 2

Running FreeSurfer

This is an example of how to use a makefile to execute FreeSurfer's longitudinal pipeline. Note that FreeSurfer itself is a large pipeline built using `make`. However, we do not need to know that if we treat the program `recon-all` as a single executable and show how to use `make` to call it. Here, the Makefile functions more as a way to permit parallel execution of `recon-all` rather than a way to track dependencies.

The code for this example is in `oasis-longitudinal-example-small/freesurfer/Makefile`.

```
  PROJHOME=$$MAKEPIPELINES/oasis-longitudinal-sample-small
❶ SUBJECTS_DIR=$(PROJHOME)/freesurfer

  QA_TOOLS=/usr/local/freesurfer/QAtools_v1.1
  FREESURFER_SETUP = /usr/local/freesurfer/stable5_3/SetUpFreeSurfer.sh
  RECON_ALL = /usr/local/freesurfer/stable5_3/bin/recon-all $(RECON_FLAGS)
❷ RECON_FLAGS = -use-mritotal -nuintensitycor-3T -qcache -all  -notal-check

❸ SHELL=/bin/bash
```

❶ FreeSurfer normally likes to work with all subjects in a single directory. We set the `make` variable `PROJHOME` for convenience, and the `SUBJECTS_DIR` because it is required by FreeSurfer.

❷ Because we have multiple versions of FreeSurfer installed, and because it is possible to run FreeSurfer with different flags, we set several variables that describe what version of FreeSurfer and what options we are using in the Makefile. Note that the definition for `RECON_ALL` refers to `RECON_FLAGS` seemingly before it is set. Recall that `make` dereferences variables when it uses them, so the order that these variables are set does not matter. This is not like `bash`!

❸ By default, `make` uses `/bin/sh` to interpret recipes. Sometimes this can cause confusion, because `sh` has only a subset of the functionality of `bash`. We can avoid such confusion by setting the `make` variable `SHELL` explicitly.

```
❹ SUBJECTS=$(notdir $(wildcard $(PROJHOME)/subjects/*))
```

❹ We need to obtain a list of subject identifiers to process. Here, we form this list by using a wildcard to obtain all the subject directories in `PROJHOME` and then stripping away all the directory prefixes using the `notdir` call.

```
  SESSION=1
❺ inputdirs=$(SUBJECTS:%=%.t$(SESSION))

❻ .PHONY: qa setup freesurfer

❼ setup: $(inputdirs)

❽ %.t$(SESSION):  $(PROJHOME)/subjects/%/visit$(SESSION)/mpr-1.nifti.nii.gz
      mkdir -p $@/mri/orig; \
```

```
        cp $^ $@/mri/orig; \
        cd $@/mri/orig; \
        mri_convert mpr-1.nifti.nii.gz 001.mgz
```

5 This Makefile is intended to handle a longitudinal acquisition. Normally, one indicates the timepoint (here, the `SESSION` variable indicates the timepoint) by appending some suffix to the subject identifier. Here, we append the suffix `.t1` to each subject identifier to indicate that we are processing the first session. To run the makefile on the second timepoint, one could either edit it, or set this variable when calling `make` as follows:

```
$ make SESSION=2
```

6 We define three targets that do not correspond to files, so these are denoted as phony targets.

7 The phony target `setup` depends upon the input directories we defined in **6** .

8 This recipe creates the input directories by transforming the first MPRAGE image from the subject directory into mgz format. More complicated recipes may include conditionally choosing one of multiple MPRAGE images, using two images if available, and so forth.

```
freesurfer: $(inputdirs:%=%/mri/aparc+aseg.mgz)

9  %.t$(SESSION)/mri/aparc+aseg.mgz: $(PROJHOME)/subjects/%/visit$(SESSION)/mpr-1.
   nifti.nii.gz
        rm -rf `dirname $@`/IsRunning.*
        source $(FREESURFER_SETUP) ;\
        export SUBJECTS_DIR=$(SUBJECTS_DIR) ;\
        $(RECON_ALL) -subjid $*.t$(SESSION) -all
```

9 FreeSurfer creates many output files when it runs. Here, we select one of the critical files that should exist upon successful completion, `mri/aparc+aseg.mgz` to be the target of this rule. It depends upon the directory having been created so that we can call `recon-all` by specifying the subject directory. You might think that it would be wise to specify multiple FreeSurfer output files as targets. In this case, if the multiple targets are specified using a pattern rule, the recipe would be executed only once to create the targets. However, if we did not use a pattern rule, the recipe could be executed once per target. This is clearly not the intended behavior. To avoid confusion, we usually pick a single late-stage output file to be the target.

```
10 qa: $(inputdirs:%=QA/%)

11 QA/%: %
        source $(FREESURFER_SETUP) ;\
        $(QA_TOOLS)/recon_checker -s $*
```

10 We can create a number of quality assurance (QA) images from the FreeSurfer directories using the `recon_checker` program. The `qa` target depends upon directories within the `QA` subdirectory. These are created by `recon_checker` in **12** .

```
12 Makefile.longitudinal:
        $(PROJHOME)/bin/genctlongitudinalmakefile > $@
```

11 After all the cross-sectional runs have been completed, we can run the longitudinal pipeline. The first step in this pipeline is to create an unbiased template from all timepoints for each subject. The second step is to longitudinally process each timepoint with respect to the template.

Here, we have a bit of a problem specifying these commands to `make` because each subject may have a different number of timepoints and subjects may be missing a timepoint that is not the first or last. The syntax of the `recon-all` command to create an unbiased template does not lend itself well to using wildcards to resolve these issues:

```
$ recon-all -base <templateid> -tp <tp1id> -tp <tp2id> ...  -all
```

We solve these problems by writing a shell script that generates a correct Makefile (`Makefile.longitudinal`). This is an example of taking a "brute force" approach rather than trying to use pattern rules or something more sophisticated. It gets the job done.

The new makefile defines a target `longitudinal`, and can be called as follows, adding additional flags for parallelism.

```
$ make -f Makefile.longitudinal longitudinal
```

Example 3

DTI Distortion Correction with Conditionals

Daniel J. Peterson[1]

In this example, we will use a Makefile to apply the appropriate distortion correction procedure automatically, using conditionals. The code for this example may be found in `dti_suceptibility_correction/subjects`, in Makefiles in the directories `fugue_DTI`, `topup_DTI`, and `toy_test` as described below.

First, a bit of background: The most common way to acquire a series of MRI images as quickly as possible is to use what is called an "echo-planar imaging readout," or "EPI." A drawback that comes with this rapid acquisition is that EPI images are prone to distortions due to an uneven magnetic field. Sometimes these distortions are called "susceptibility-induced distortions," because it is the differing magnetic susceptibility of tissue, bone, and air that causes the magnetic field to be uneven. Fortunately, because these distortions are constant and predictable within an imaging session, they can be undone.

We will consider two ways of undoing these distortions, each requiring at least one extra image to be acquired. The first method is to acquire an image of the uneven magnetic field, called a "fieldmap." This image contains a map of the deviation from the main magnetic field, in radians/sec (the resonance frequency of the water protons is directly proportional to the strength of the magnetic field as per the Larmor equation: $\omega = \gamma B_0$). To do this we will use a tool called `fugue`, which is distributed as part of FSL. `fugue` will convert this fieldmap into a nonlinear warp, and then we can apply that warp to undo the susceptibility-induced distortions. The other method is to acquire an additional image with the EPI readout going in the other direction, meaning that the direction of the susceptibility-induced distortion will be reversed. We can then use a tool called `topup` (also from FSL), that will warp two images with opposite distortions towards each other, until they meet in the middle. We can then apply this computed warp to the rest of our data.

These are two different procedures that require different sets of files (an acquisition parameters file for `topup` and a fieldmap for `fugue`), but can be part of an otherwise similar pipeline. We can use `make` to sense which procedure to use based on what files are present, and assemble the appropriate prerequisites.

To help understand the behavior of `make`, we have created an example of a "toy" makefile, which does not call any real programs, and can operate on empty "dummy" files. When debugging and developing makefiles it can be useful to write such simplified makefiles, and run them with `make -n`. The code for this Makefile is in `dti_suceptibility_correction/subjects/toy_test/Makefile`.

1

```
SDC_METHOD = $(shell if [ -f fieldmap ] ; then echo FUGUE; \
             elif [ -f acqparams ] ; then echo TOPUP; \
             else echo FALSE ; fi)

motion_corrected_dataset: raw_diffusion_dataset
    toy_eddy.sh raw_diffusion_dataset
```

[1] djpeters@uw.edu

```
topup_result: raw_diffusion_dataset acqparams
    toy_topup.sh raw_diffusion_dataset acqparams

❷ ifeq ($(SDC_METHOD),TOPUP)

fully_corrected_diffusion_dataset: raw_diffusion_dataset topup_result
    toy_eddy.sh raw_diffusion_dataset topup_result

else ifeq ($(SDC_METHOD),FUGUE)
fully_corrected_diffusion_dataset: motion_corrected_dataset fieldmap
    toy_fugue.sh motion_corrected_dataset fieldmap

else
❸ $(error ERROR: neither fieldmap for FUGUE \
               nor acquisition parameter file for TOPUP were found)
endif

tensor: fully_corrected_diffusion_dataset
    toy_solve_tensor.sh fully_corrected_diffusion_dataset
```

❶ If there is a file called `fieldmap` in the working directory we want to use `fugue`, and if `acqparams` exists instead, we want to use `topup`.

❷ When `make` runs, it will insert a different recipe into the set of rules depending on the value of `SDC_METHOD`. The conditional block extends until `endif`. The `else ifeq` statement in the middle is part of the same conditional block (i.e. you only need one `endif`).

❸ It's always a good idea to halt and raise an error if none of the expected conditions are met.

If we run this makefile to build the `tensor` target (using the `-n` flag) in a directory that contains both `raw_diffusion_dataset` and `fieldmap`, the result looks like this:

```
$ make -n tensor
toy_eddy.sh raw_diffusion_dataset
toy_fugue.sh motion_corrected_dataset fieldmap
toy_solve_tensor.sh fully_corrected_diffusion_dataset
```

However, if both `raw_diffusion_dataset` and `acqparams` (and not `fieldmap`) are in the directory, we see:

```
$ make -n tensor
toy_topup.sh raw_diffusion_dataset acqparams
toy_eddy.sh raw_diffusion_dataset topup_result
toy_solve_tensory.sh fully_corrected_diffusion_dataset
```

Figure 3.1 is a flowchart of the toy makefile.

If we were to implement this process in a `bash` script, the earlier "upstream" processes would need to be part of the conditional block. An advantage of using `make` for workflows is that *only* the part that causes the "split" in the branching logic needs to be surrounded by the if-then-else-end logic. Also note that if we were using `topup` with a fieldmap, but for some reason we wanted to do just the motion correction (like for QC, or for debugging purposes), that unused "branch" of the makefile would be available with a simple `make motion_corrected_dataset` command.

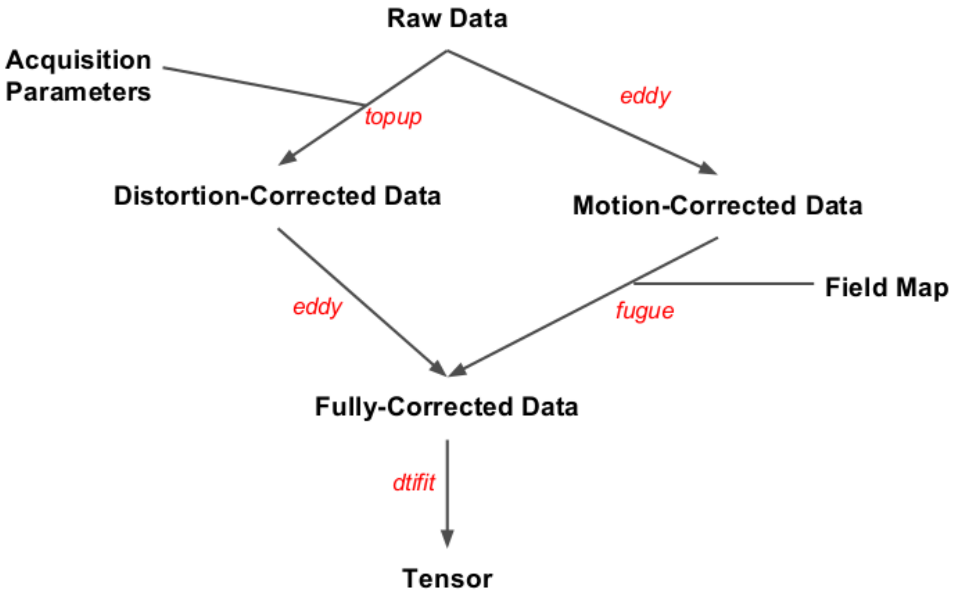

Figure 3.1: Flowchart of the Toy Makefile

Here's the full Makefile, located in `dti_suceptibility_correction/subjects/fugue_DTI` and `dti_suceptibility_correction/subjects/topup_DTI`. You can see each of these files is in fact a symbolic link to the same file, located in `dti_suceptibility_correction/lib/diffusion.Makefile`.

```
ifeq "$(origin MAKEPIPELINES)" "undefined"
MAKEPIPELINES=/project_space/makepipelines
endif

PROJECT_DIR=$(MAKEPIPELINES)/dti_suceptibility_correction

SDC_METHOD = $(shell if [ -f fieldmap.nii.gz ] ; then echo FUGUE; \
                     elif [ -f acqparams.txt ] ; then echo TOPUP; \
                     else echo FALSE ; fi)


1 NUM_DIFFUSION_VOLS =$(shell fslval raw_diffusion.nii.gz dim4 | tr -d
    '\040\011\012\015')


2 EDDY_ITERATIONS = 1
TOPUP_MODE=fast
ECHO_SPACING =.00072
UNWARP_DIRECTION=y-

.PHONY: clean tensor

3 mec_diffusion.nii.gz: raw_diffusion.nii.gz bval bvec brain_mask.nii.gz
```

```
        echo "0 1 0 0.072" > temp_acqparams.txt ;\
        for i in `seq 1 $(NUM_DIFFUSION_VOLS)`; do echo 1 >> temp_index.txt ; done
            ;\
        eddy --imain=raw_diffusion.nii.gz --mask=brain_mask.nii.gz \
                --index=temp_index.txt --acqp=temp_acqparams.txt --bvecs=bvec \
                --bvals=bval --out=mec_diffusion --niter=$(EDDY_ITERATIONS) \
                --verbose  ;\
        rm temp_acqparams.txt temp_index.txt

❹ topup_results_movpar.txt: raw_diffusion.nii.gz acqparams.txt
        fslroi raw_diffusion.nii.gz S0_images.nii.gz 0 2 ;\
        topup --imain=S0_images --datain=acqparams.txt \
                --config=$(PROJECT_DIR)/lib/b02b0_$(TOPUP_MODE).cnf \
                --out=topup_results --fout=field_est --iout=unwarped_S0_images \
                --verbose

ifeq ($(SDC_METHOD),TOPUP)
sdc_mec_diffusion.nii.gz: raw_diffusion.nii.gz topup_results_movpar.txt index.txt
        eddy --imain=raw_diffusion.nii.gz --mask=brain_mask --acqp=acqparams.txt \
                --index=index.txt --bvecs=bvec --bvals=bval \
                --topup=topup_results \
                --out=sdc_mec_diffusion.nii.gz --niter=$(EDDY_ITERATIONS) \
                --verbose

else ifeq ($(SDC_METHOD),FUGUE)
sdc_mec_diffusion.nii.gz: mec_diffusion.nii.gz fieldmap.nii.gz
        fugue --loadfmap=fieldmap.nii.gz --dwell=$(ECHO_SPACING) \
                -i mec_diffusion.nii.gz -u sdc_mec_diffusion.nii.gz \
                --unwarpdir=$(UNWARP_DIRECTION) -v
else
$(error ERROR: neither fieldmap for FUGUE nor acquisition parameter file for TOPUP
    were found)
endif

tensor: sdc_mec_diffusion.nii.gz brain_mask.nii.gz bvec bval
        dtifit -k sdc_mec_diffusion.nii.gz -r bvec -b bval -m brain_mask -o dti

clean:
        rm -f dti_* sdc_mec_diffusion.* mec_diffusion.* S0_images* \
        field_est.nii.gz topup_results* unwarped_S0_images.nii.gz
```

❶ `fslval` returns a trailing space as part of its output, which we pipe to `tr` for deletion.

❷ The settings here are for a quick test run for demonstration purposes. For more accurate (but slower) processing, `TOPUP_MODE` can be changed to 'accurate', and `EDDY_ITERATIONS` can be increased to 5. Placing the settings clearly in the Makefile makes it easy to extract them for quality assurance reports.

❸ In addition to running `eddy`, this recipe creates a simple acquisition parameters file and a simple index file (`eddy` requires that you supply one). This tells `eddy` that all the images were acquired with the phase encoding along the same direction. These files are deleted afterwards.

❹ The first two images are assumed to be the two non-diffusion weighted images (i.e. the S_0 images), with the phase encoding along different directions.

There are a few more files and commands here than in the toy example, but the basic structure is the same. It's still missing some elements of a full-featured DTI preprocessing pipeline (for example, unwarping the brain mask, coregistration of the fieldmap with the diffusion data, and rotation of the b-vectors), but this example illustrates how using conditional statements in makefiles can make them more robust and versatile.

More information about the options and the formats of the files supplied to `eddy`, `topup`, and `fugue` is available on the FSL website.

Example 4

Quantifying Arterial Spin Labeling Data

Swati D. Rane[1]

This is an example of how to use a makefile to quantify cerebral blood flow (CBF) from pseudo-continuous arterial spin labeling (pCASL) data.

The code for this example is in `$MAKEPIPELINES/ASLTestsubject/S001/Makefile`. S001 is the name of the subject and the folder name. In this example we assume we have one folder per subject. The folder name reflects the subject name and contains the `SUBJECTNAME_ASL.nii.gz` and `SUBJECTNAME_M0.nii.gz` files. The ASL file contains alternating volumes from the control and label acquisitions, where the odd volumes are control volumes and the even volumes are the label volumes. The M0 file is a single 3D volume of the reference proton density weighted image.

A unique feature of this example is that we have performed surround subtraction in MATLAB to improve the signal-to-noise ratio of the ASL data. We have included a standalone MATLAB application that can be installed with the necessary runtime binaries and libraries to complete this step. Note that this approach does not require you to have MATLAB installed on your computer. Similarly, if you have a limited number of floating licenses available, deploying a standalone MATLAB application does not require a floating license.

If you are not at IBIC, before running this example you must install the MATLAB runtime libraries and surround subtraction application as follows:
Change directory to `ASLTestsubject/bin` and run the program `SSInstaller_mcr.install` as shown in Figure 4.1.

```
cd $MAKEPIPELINES/ASLTestsubject/S001/Makefile
./SSInstaller_mcr.install
```

Figure 4.1: Installing MATLAB Runtime Libraries and surround subtraction application

The installer will, perhaps after some delay, open a splash screen with an opportunity to change connection settings. Just click next. The next screens will prompt for a location for the installation folder (see Figure 4.2). Enter the location of `$MAKEPIPELINES/ASLTestsubject/bin` whenever asked. Note that here, the value of `MAKEPIPELINES` is set to `/project_space/makepipelines`. In your environment this will be different.

[1] srleven@uw.edu

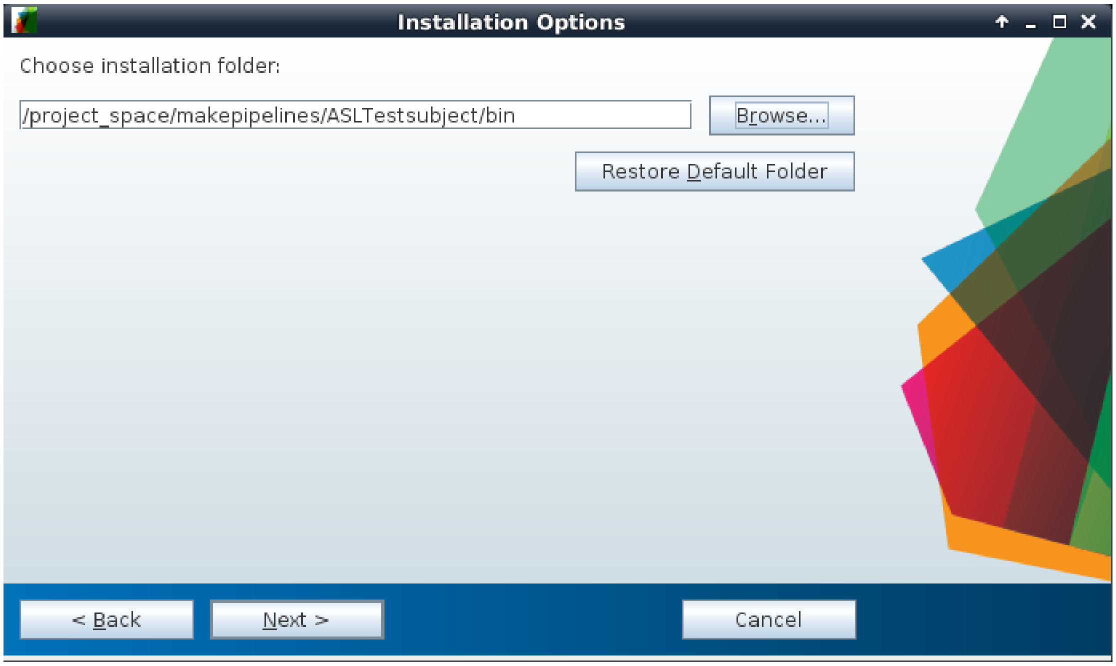

Figure 4.2: Entering location of installation folder for Surround Subtraction standalone MATLAB application.

```
SHELL=/bin/bash

❶ tau = 1.5
pld = 1.525
label_eff = 0.85
T1b = 1.627
lambda=0.9

ifeq "$(origin MAKEPIPELINES)" "undefined"
MAKEPIPELINES=/project_space/makepipelines
endif

PROJECT_HOME = $(MAKEPIPELINES)/ASLTestsubject/S001

❷ #FSLDIR=/usr/share/fsl/5.0
FSL_PATH = ${FSLDIR}/bin


❸ MATLAB_PATH = $(MAKEPIPELINES)/ASLTestsubject/bin
STD_BRAIN2mm = ${FSLDIR}/data/standard/MNI152_T1_2mm_brain.nii.gz
STD_BRAIN2mm_MASK = ${FSLDIR}/data/standard/MNI152_T1_2mm_brain_mask.nii.gz
STD_BRAIN2mm_GM = ${FSLDIR}/data/standard/tissuepriors/avg152T1_gray.img

SUBJECT = $(shell basename ${PROJECT_HOME})
```

❶ CBF quantification requires specific sequence parameters such as label duration (`tau`), post labeling delay (`pld`), longitudinal relaxation, T1 of blood (`T1b`). `label_eff` and `lambda` are constants defining the labeling efficiency for pCASL and the tissue-blood partition coefficient respectively. For convenience, we have predefined the paths for subject directory `PROJECT_HOME` as well as standard template brains and masks under `STD_BRAIN2mm`, `STD_BRAIN2mm_MASK`, and `STD_BRAIN2mm_GM`.

❷ Because we have multiple versions of FSL installed, we set variables to describe what version of FSL we are using in the Makefile.

❸ The MALTAB app is a `.mcr` file. Double clicking will open an installation window to copy the codes to a user-specified location. In this project, we have copied these files and folders in the `bin` directory under `ASLTestSubject`. This can serve as the common directory for all subjects. The `surround_subtraction.sh` is the shell file that calls `run_Surround_Subtraction.sh` generated by the MATLAB app.

```
.PHONY =  all clean
all:${SUBJECT}_CBF_MNI.nii.gz  QA/ASL.gif

${SUBJECT}_mc_ASL.nii.gz: ${SUBJECT}_ASL.nii.gz ${SUBJECT}_M0.nii.gz
	${FSL_PATH}/mcflirt -in $< -reffile $(word 2,$^) -out $@;
```

In this segment of code, we motion correct the ASL data using MCFLIRT in FSL. Because the ASL and M0 data are acquired as separate scans, all ASL volumes are registered to the M0 volume. This is unlike the motion correction typically performed in BOLD fMRI where the central dynamic is considered as the reference volume. All remaining volumes are registered to this central volume.

```
#Implement surround subtraction for calculating differences in MATLAB
❹ ${SUBJECT}_DeltaM.nii.gz: ${SUBJECT}_mc_ASL.nii.gz
	${MATLAB_PATH}/surround_subtraction.sh $< ${MATLAB_PATH}

❺ ${SUBJECT}_CBF.nii.gz:${SUBJECT}_DeltaM.nii.gz ${SUBJECT}_M0.nii.gz;
	${FSL_PATH}/fslmaths $< -div $(word 2,$^) temp;\
	${FSL_PATH}/fslmaths temp -mul \
	$(shell echo "scale=5; 6000*${lambda};" | bc -l) temp ;\
```

```
        ${FSL_PATH}/fslmaths temp -mul  \
        $(shell echo "scale=5; e(${pld}/${T1b});" | bc -l) temp;\
        ${FSL_PATH}/fslmaths temp -div \
        $(shell echo "scale=5; 2*${label_eff}*${T1b}*(1-e(-${tau}/${T1b}));" \
        | bc -l) $@;\
        rm temp.nii.gz
```

In this section, we compute absolute values of CBF. ❹ The `surround_subtraction.sh` script evaluates the dimensions of the ASL data (rows, columns, slices) and calls the MATLAB script via `run_Surround_-Subtraction.sh`. ❺ CBF is computed in ml/100gm/min, from the ASL white paper (Alsop et al., 2015).

```
${SUBJECT}_CBF_MNI.nii.gz: ${SUBJECT}_M0.nii.gz ${SUBJECT}_CBF.nii.gz
        ${FSL_PATH}/flirt -in $< -ref ${STD_BRAIN2mm} -out temp.nii.gz \
        -omat cbf_to_MNI.mat -bins 256 -cost corratio -searchrx -90 90 \
        -searchry -90 90 -searchrz -90 90 -dof 12  -interp trilinear;\
        ${FSL_PATH}/flirt -in $(word 2, $^) -ref ${STD_BRAIN2mm} \
        -out temp.nii.gz -applyxfm -init cbf_to_MNI.mat;\
        ${FSL_PATH}/fslmaths temp.nii.gz -mul ${STD_BRAIN2mm_MASK} $@;\
        rm temp.nii.gz
```

This portion of the Makefile registers the CBF map to MNI space and removes background. We did not have a corresponding T1 image for this ASL data. Hence we regististered the M0 image directly to the the standard 2mm MNI template available in FSL. We then applied the subsequent transformation matrix to the CBF map.

Ideally, if a T1 image were available, this step would be different. We would register the M0 image to the subject's native T1 image. The T1 image would be registered to the MNI template, and we would combine the two transformation matrices to register the CBF map to MNI space.

```
QA/ASL.png:${SUBJECT}_CBF_MNI.nii.gz
        mkdir -p ${PROJECT_HOME}/QA;\
        ${FSL_PATH}/slicer $< -i 0 150 -a QA/ASL.png;\
        ${FSL_PATH}/fslmaths ${STD_BRAIN2mm_GM} -thr 130 temp;\
        ${FSL_PATH}/fslmaths $< -mas temp temp;\

        gm_cbf=`${FSL_PATH}/fslstats -t temp -M`;\
        echo $$gm_cbf;\
        echo "---" > QA/ASL_QA.Rmd;\
        echo "output: html_document" >>QA/ASL_QA.Rmd;\
        echo "---" >> QA/ASL_QA.Rmd;\
        echo "# ASL QA" >> QA/ASL_QA.Rmd;\
        echo '<img src="../QA/ASL.png" width=900px; />' >> QA/ASL_QA.Rmd;\
        echo        >> QA/ASL_QA.Rmd;\
    ❻   echo  'Typical GM CBF = 49(2) ml/100gm/min'  >> QA/ASL_QA.Rmd;\
        echo        >> QA/ASL_QA.Rmd;\
        echo  'This subject, GM CBF = '$$gm_cbf >> QA/ASL_QA.Rmd;\
    ❼   R -e 'library("knitr");knitr::knit2html("QA/ASL_QA.Rmd","QA/ASL_QA.html")'
```

In the last section, we make a QA report for the ASL quantification process. ❻ Typical CBF values in the gray matter in healthy adults is 49 ± 2 ml/100gm/min and is provided as reference. This value is lower than the standard 55 ml/100gm/min, because we did not correct for partial volume effects. In this piece of code, we evaluate CBF in the gray matter. Because no native T1 images are available for this subject, we used the the standard 2mm resolution gray matter mask available in FSL. As part of the QA, we generate an image of CBF maps along the mid-sections in all three planes.

❼ The image for QA and the CBF values (typical and measured) are embedded in a R markdown file which is then knit to obtain an HTML report for every subject.

```
clean:
```

```
        rm -f $(wildcard [A-Z][0-9][0-9][0-9]_CBF*.nii.gz) \
        $(wildcard [A-Z][0-9][0-9][0-9]_Del*.nii.gz) dimen.txt;\
        rm  -f $(wildcard [A-Z][0-9][0-9][0-9]_mc_*.nii.gz) temp.nii.gz \
        cbf_to_MNI.mat ASL_QA.md;\
        rm -r QA
```

The final target `clean` removes intermediary NIFTI files and matrix or text files using wildcards. Preprocessed images generated for QA reports can also be cleaned up this way.

Example 5

Processing Scan Data for a Single Test Subject

Testsubject Main Makefile

This is an example of a subject-specific makefile that includes pipelines (written using `make`) that have been developed by several people to process different types of subject-level MRI data. Because there is only one subject in this example, this subject specific makefile is not a symbolic link, as it is in the `oasis-longitudinal-sample-small` directory.

The code for this example is in `$MAKEPIPELINES/testsubject/test001/Makefile`.

```
❶ .PHONY = etiv fast flex robex freesurferskullstrip

❷ FSL_DIR=/usr/share/fsl/5.0

STD_BRAIN=$(FSL_DIR)/data/standard/MNI152_T1_2mm.nii.gz

ifeq "$(origin MAKEPIPELINES)" "undefined"
MAKEPIPELINES=/project_space/makepipelines
endif

❸ t1subj := $(shell pwd)
subject := $(notdir $(t1subj))

PROJECT_HOME=$(MAKEPIPELINES)/testsubject/

❹ include $(PROJECT_HOME)/lib/makefiles/help_system.mk
include $(PROJECT_HOME)/lib/makefiles/resting.mk
include $(PROJECT_HOME)/lib/makefiles/xfm.mk
include $(PROJECT_HOME)/lib/makefiles/fcconnectivity.mk
include $(PROJECT_HOME)/lib/makefiles/QA.mk
include $(PROJECT_HOME)/lib/makefiles/methodsgenerator.mk

❺ export OMP_NUM_THREADS=1

SHELL=/bin/bash

❻ FLEXPATH=$(PROJECT_HOME)/bin/wmprogram/sb/cross_platform/scripts
```

❶ We begin by specifying our phony targets. These will be defined later. ❷ This line that sets `FSL_DIR` will be commented out in your makefile, but you should find out where your version of FSL is installed and make sure that it is correct. If we do not specify the version of the programs that we run in a makefile, `make`

will use the individual's PATH variable to find them. Because different people may have different PATH variables, this will result in unpredictable results (the opposite of reproducibility!) Recall that make inherits variables from your environment, and you can override them by setting them.

❸ It is useful to have a variable to refer to the subject. Here, we simply obtain the subject from the last part of the directory name. This won't work for more complicated linking structures as described in make in Context.

❹ We include lots of other makefiles that we need. This keeps this makefile short and readable.

❺ Certain programs do their own parallelization on computers with multiple processing elements (cores). To turn this off, we override the environment variable OMP_NUM_THREADS. This allows us to use the -j flag to make to parallelize execution of the entire makefile.

❻ FLEX is a program for white matter hyperintensity quantification that we use for this project. This variable simply specifies its location. If you do not have it installed you just won't be able to run that part of the processing. It is not mandatory.

```
❼ FLAIR = $(shell if [ -f 00_NOFLAIR ] ; then echo false; else echo true; fi)

  HAVET1 = $(shell if [ -f 00_NOT1 ] ; then echo false; else echo true; fi)

❽ ifeq ($(HAVET1),true)
❾ all: $(call print-help,all,Do skull stripping, etiv, HC volume calculation)
     T1_skstrip.nii.gz first etiv
  else
  all:  $(call print-help,all,Do skull stripping, etiv, HC volume calculation)
        @echo "Subject is missing T1 - nothing to do here."
  endif
```

❼ In large and complicated studies, a certain type of image may be missing, but this does not stop us from processing all the data that we have. Here, we test for a FLAIR acquisition by looking for a marker file in the directory called 00_NOFLAIR (and similarly for the T1).

❽ Here we modify the target all depending on whether or not the subject has a T1 image. We also use the help system described in Advanced Topics & Quality Assurance with R Markdown to document what this target does.

```
T1_skstrip.nii.gz: T1.nii.gz
        bet $< $@ -B

robex: $(call print-help,robex,Alternate skull stripping with ROBEX) T1.nii.gz
        $(PROJECT_HOME)/bin/ROBEX/runROBEX.sh T1.nii.gz T1_skstrip.nii.gz

freesurferskstrip: $(call print-help,freesurferskullstrip, Alternate skull
   stripping with FreeSurfer) $(PROJECT_HOME)/freesurfer/$(subject)/mri/brain.mgz
        subj=$(subject) ;\
        mri_vol2vol --mov $(PROJECT_HOME)/freesurfer/$${subj}/mri/brain.mgz \
        --targ $(PROJECT_HOME)/freesurfer/$${subj}/mri/rawavg.mgz \
        --regheader --o brain-in-rawavg.mgz ;\
        mri_convert brain-in-rawavg.mgz brain-in-rawavg.nii.gz ;\
        fslreorient2std brain-in-rawavg.nii.gz T1_skstrip.nii.gz ;\
```

We have rules in this makefile for three methods of skull stripping. The default is simply to call bet with the bias correction option (which worked well on the data for the study this makefile was modeled after). However, when this method did not work well, we tried alternative methods using ROBEX and FreeSurfer. Note that because the targets robex and freesurferskstrip are phony, they can be created at any time and will always overwrite T1_skstrip.nii.gz.

```
etiv: $(call print-help, etiv, Estimation of ICV using enigma protocol) eTIV.csv
```

```
brain_to_std.mat brain_to_std.nii.gz: T1_skstrip.nii.gz
	flirt -in $< -ref $(STD_BRAIN) -omat $@ -out brain_to_std.nii.gz

eTIV.csv: brain_to_std.mat
   10   etiv=‘$(PROJECT_HOME)/bin/mat2det brain_to_std.mat \
	| awk ’{print $$2 }’‘ ;\
	echo  $(subject)","$$etiv > $@
```

We estimate intracranial volume (ICV) using the ENIGMA protocol (as described in Getting Down and Dirty with `make`). This approach calculates the inverse determinant of the linear transformation of the T1 image to standard space. This is a scaling factor that we can multiply the volume of the standard space brain by to obtain an estimated ICV volume.

9 Note that when calling the `mat2det` script and echoing the results to a file, we need two dollar signs (`$$`) for every one that we intend to pass to the shell.

```
first: first_all_fast_firstseg.nii.gz T1.nii.gz hippo.csv

first_all_fast_firstseg.nii.gz : T1.nii.gz
	$(PROJECT_HOME)/bin/run_first_all_edit -s "L_Hipp,R_Hipp"  -d -i T1.nii.gz
	    -o first

hippo.csv: first_all_fast_firstseg.nii.gz
	rh=‘fslstats $< -u 54 -l 52 -V|awk ‘{print $$2}’’ ;\
	lh=‘fslstats $< -u 18 -l 16 -V|awk ‘{print $$2}’’ ;\
	echo $$lh $$rh > hippo.csv
```

We run FSL FIRST to calculate hippocampal volumes. We put these into a comma separated value file to make it easy to remember how to extract these numbers from the `first_all_fast_firstseg.nii.gz` and check them (or include them in a QA report) although there is no reason you could not write a separate program to gather them all.

```
ifeq ($(FLAIR),true)
flex: $(call print-help,flex, Run flex for white matter hyperintensity
   quantification) flair.nii.gz flair_skstrip.hdr flair_skstrip_flwmt_lesions.hdr
    wmhstats.csv

flair_restore.nii.gz: flair.nii.gz
	fast -B -o flair -t 2 $<

flair_skstrip.nii.gz: flair_restore.nii.gz
	bet $< $@ -R

flair_skstrip.hdr: flair_skstrip.nii.gz
	fslchfiletype ANALYZE $< $@

flair_skstrip_flwmt_lesions.hdr: flair_skstrip.hdr
	@echo "Flex processing " $<
	$(FLEXPATH)/sb_flex -fl $<

flair_skstrip_renamed.nii.gz: flair_skstrip.nii.gz
	cp $< $@

flair_wmh_mask.nii.gz: flair_skstrip_flwmt_lesions.hdr
	fslmaths $< -uthr 1 $@

else
flex:
```

```
	@echo No FLAIR, nothing to do
endif

wmhstats.csv: flair_skstrip_flwmt_lesions.hdr flair_skstrip_renamed.nii.gz
	@echo Writing wmhstats.csv
	tot=`fslstats  flair_skstrip_renamed.nii.gz -V | awk '{print $$2}''; \
	wmh=`fslstats  flair_skstrip_flwmt_lesions.hdr -u 2 -V \
	| awk '{print $$2}'' ; \
	per=`echo $$wmh $$tot | awk '{print ($$1/$$2)*100}'' ; \
	echo  $(subject)", "$$wmh", " $$per >> $@
```

These are rules to run FLEX, a program for white matter hyperintensity quantification from FLAIR images. This example comes from a multi-site study where not all subjects obtained a viable FLAIR scan. If they do have a FLAIR scan, we bias correct, skull strip, and process the data using the program `sb_flex`. The volume of white matter hyperintensities (absolute volume and percent of skull stripped brain) are written to `wmhstats.csv`. If we don't have a FLAIR scan, there is nothing to do. You may not have FLEX installed on your system in which case you can disregard this part of the makefile.

```
archive:
	rm -rf flair_bc_*
	rm -rf flair_skstrip_axcor* flair_skstrip_flf_* flair_skstrip_WMT*
	rm -rf *~ \#* brain_to_std.nii.gz flair_skstrip.hdr flair_skstrip.img

clean:  $(call print-help,clean,Clean up everything from all makefiles)
    clean_rest clean_qa clean_transform clean_fcconnectivity clean_provenance
	rm -rf flair_skstrip* flair_pve* flair_restore* *~ wmhstats.csv eTIV.csv \
	brain_to_std* flair_mixeltype.nii.gz T1_skstrip_mask.nii.gz \
	first-L_Hipp* first-R_Hipp* T1_to_std_sub* \
	T1_skstrip.nii.gz hippo.csv first*
```

Finally, we define two targets to help us clean up. The first target, `archive`, is intended to remove files that we don't need when we intend to "tidy up". What we remove here is very subjective and dependent upon the needs of the researchers.

The second target, `clean` is intended to remove everything from all makefiles to clean up everything. Note that `clean` depends upon targets such as `clean_transform` and `clean_rest` that are defined in the makefiles we have included at the beginning.

Testsubject FreeSurfer

This is an example of how to use a makefile to execute FreeSurfer in a cross-sectional context (in contrast to Testsubject FreeSurfer), which describes a longitudinal pipeline. In addition, here we use FreeSurfer to create a brain mask for skull stripping.

The code for this example is in `$MAKEPIPELINES/testsubject/freesurfer/Makefile`.

```
ifeq "$(origin MAKEPIPELINES)" "undefined"
MAKEPIPELINES=/project_space/makepipelines
endif

PROJHOME=$(MAKEPIPELINES)/testsubject
```

This first section of the code looks to see if the environment variable `MAKEPIPELINES` is set. This allows people who are not at IBIC to override the default location of these files.

```
❶ include $(PROJHOME)/lib/makefiles/help_system.mk

❷ SUBJECTS=test001

export SUBJECTS_DIR=$(PROJHOME)/freesurfer
export FREESURFER_SETUP = /usr/local/freesurfer/stable5_3/SetUpFreeSurfer.sh
export RECON_ALL = /usr/local/freesurfer/stable5_3/bin/recon-all
export TKMEDIT = /usr/local/freesurfer/stable5_3/bin/tkmedit
QA_TOOLS=/usr/local/freesurfer/QAtools_v1.1

export SHELL=/bin/bash

❸ outputstats=$(SUBJECTS:%=%/mri/aparc+aseg.mgz) $(SUBJECTS:%=%/mri/brainmask.mgz)
        $(SUBJECTS:%=%/mri/brainmask.nii.gz)

❹ inputdirs = $(SUBJECTS:%=%)

.PHONY: all clean qa freesurfer

.SECONDARY: $(inputdirs) $(outputstats)
```

This portion of the Makefile defines key variables and targets. ❶ We make use of the help system described in Advanced Topics & Quality Assurance with R Markdown. ❷ There is only one subject here, so for clarity we simply write it out. However, in real life you would have many subjects, and obtain them through a wildcard or from a file (see Obtaining a List of Subjects). ❸ We use pattern substitution to specify all of the output targets we wish to create. These are the `aparc+aseg.mgz` file, the `brainmask.mgz` file, and the brainmask converted to NIfTI format (`brainmask.nii.gz`). Note that all of these files are designated as SECONDARY targets so that they are not deleted at the end! ❹ We also use pattern substitution to set up the input directories.

```
all: $(call print-help,all,Setup directories, Run Freesurfer, and Run QA) setup
    freesurfer qa

freesurfer: $(call print-help,freesurfer,Run FreeSurfer) $(outputstats)
```

The `all` and `freesurfer` targets here are documented using the help system.

```
%/mri/brainmask.mgz: %
        subj=$* ;\
        export FREESURFER_SETUP=$(FREESURFER_SETUP) ;\
        export WATERSHED_PREFLOOD_HEIGHTS='05 15 25 35' ;\
        rm -rf $${subj}/scripts/IsRunning.* ;\
        source $$FREESURFER_SETUP ;\
```

```
        export SUBJECTS_DIR=$(SUBJECTS_DIR) ;\
        recon-all  -subjid  $${subj} -multistrip -autorecon1 ;\
        height=‘cat $${subj}/mri/optimal_preflood_height’ ;\
        if [[ $$height == 05 ]]; then max=$$(( $$height + 5 )); \
        WATERSHED_PREFLOOD_HEIGHTS=‘echo $$height $$max’; \
        else min=$$(( $$height - 5 )); max=$$(( $$height + 5 )); \
        WATERSHED_PREFLOOD_HEIGHTS=‘echo $$min $$height $$max’; fi ;\
        export WATERSHED_PREFLOOD_HEIGHTS ;\
        recon-all -s $${subj} -multistrip -clean-bm -gcut
```

This rule creates the brain mask (in .mgz format). We use the `-multistrip` flag to `recon-all` which allows us to try multiple preflood heights, trying different thresholds automatically. Then `recon-all` is continued using the optimal height. Note that this approach will begin a separate process for each preflood height (e.g., four processes). This makes it a bit hard to run this pipeline on many brains in parallel. Normally, we use this approach when we are processing subjects one at a time after they have been scanned.

```
%/mri/brainmask.nii.gz: %/mri/brainmask.mgz
        export FREESURFER_SETUP=$(FREESURFER_SETUP) ;\
        source $$FREESURFER_SETUP ;\
        mri_convert $< $@
```

This rule converts the brain mask from `mgz` format to NIfTI format.

```
%/mri/aparc+aseg.mgz:  %/mri/brainmask.mgz
        subj=$* ;\
        export FREESURFER_SETUP=$(FREESURFER_SETUP) ;\
        source $$FREESURFER_SETUP ;\
        export SUBJECTS_DIR=$(SUBJECTS_DIR) ;\
        recon-all -s $${subj} -autorecon2 -autorecon3
```

This rule finishes the remaining stages of `recon-all`. It depends upon the brain mask being generated (the result of the previous rule).

```
 setup: $(call print-help,setup,Setup subject directories) $(inputdirs)

%:  $(PROJHOME)/%/T1.nii.gz
        mkdir -p $@/mri/orig; \
        cp $^ $@/mri/orig; \
        cd $@/mri/orig; \
        mri_convert *nii.gz 001.mgz
```

The `setup` target depends upon the T1 image in the subject directory (here, in `$PROJHOME`). It creates a FreeSurfer style subject directory, copies the file there, and converts it to NIfTI format.

```
qa: $(call print-help,qa, Run QA - do this interactively with screensaver \
        shut off) $(inputdirs:%=QA/%)

QA/%: %
        source $(FREESURFER_SETUP) ;\
        $(QA_TOOLS)/recon_checker -s $*
```

We run the FreeSurfer QA tools to generate images that we can put into our own reports. One problem with QA is that the images cannot be generated in parallel in batch. We include a warning in the help system that they should be generated interactively without a screensaver.

```

echo rm -rf $(inputdirs)
```

Finally, we define a `clean` target to remove all processing results.

Testsubject Transformations

This is an example of using a makefile to create a set of transformation matrices using different registration methods available in FSL.

The code for this example is in `$MAKEPIPELINES/testsubject/lib/makefiles/xfm.mk`. It is included by `$MAKEPIPELINES/testsubject/test001/Makefile`. Therefore, certain variables that this example uses are defined there. This approach helps to organize multiple makefiles and reuse rules across projects.

```
.PHONY=clean_transform transforms

transforms:  $(call print-help,xfm,Create resting state to MNI transformations)
   xfm_dir xfm_dir/MNI_to_rest.mat
```

The first line defines two phony targets (clean_transform and transforms). The `.PHONY` target can be set as many times as you need to, and note that each makefile included by `testsubject/test001/Makefile` defines phony targets.

The second target, `xfm`, uses the `print-help` call introduced in Advanced Topics & Quality Assurance with R Markdown to document this main function, to create an MNI to resting state transformation.

```
    xfm_dir:
(1)         mkdir -p xfm_dir

(2) xfm_dir/T1_to_MNI.mat: xfm_dir T1_skstrip.nii.gz
            flirt -in T1_skstrip.nii.gz -ref $(STD_BRAIN) -omat $@
```

(1) We define a target to create a directory, `xfm_dir`, to hold all of our transformations. This is handy because it allows us to reuse transformations for other analyses. We know that the registrations saved here will be checked.

(2) This is just a simple rule to call `flirt` to perform linear registration of the skull stripped T1 image to the standard brain. Note that the definition for `STD_BRAIN` comes from the including makefile, as do the rules to create the file `T1_skstrip.nii.gz`.

```
rest_dir/rest_mc_vol0.nii.gz: rest_dir/rest_mc.nii.gz
        fslroi $< $@ 0 1

xfm_dir/rest_to_T1.mat: rest_dir/rest_mc_vol0.nii.gz T1_skstrip.nii.gz
        mkdir -p xfm_dir ;\
    (3) epi_reg --epi=rest_dir/rest_mc_vol0.nii.gz --t1=T1.nii.gz \
        --t1brain=T1_skstrip.nii.gz --out=xfm_dir/rest_to_T1
```

These rules use FSL's `epi_reg` program to register the resting state data to the subject's structural data. We noticed that `epi_reg` used a lot of memory when running, limiting the number of processors that we could use in parallel to preprocess resting state data. (3) This requirement can be circumvented by using only the first volume of the resting state data, obtained in the first rule.

```
xfm_dir/T1_to_rest.mat: xfm_dir/rest_to_T1.mat
        convert_xfm -omat $@ -inverse $<

xfm_dir/MNI_to_T1.mat: xfm_dir/T1_to_MNI.mat
        convert_xfm -omat $@ -inverse $<

xfm_dir/MNI_to_rest.mat:  xfm_dir/T1_to_rest.mat xfm_dir/MNI_to_T1.mat
        convert_xfm -omat xfm_dir/MNI_to_rest.mat \
        -concat xfm_dir/T1_to_rest.mat  xfm_dir/MNI_to_T1.mat
```

We obtain the T1 to resting matrix by inverting the resting to T1 matrix, and similarly for the MNI to T1 matrix. Finally, these matrices are concatenated to create the final target, `MNI_to_rest.mat`. Notice that everything else we needed was automatically created as necessary to make this final target.

```
clean_transform:
        rm -rf  xfm_dir
```

Finally, we define a target to remove what we have created and clean up. Notice that we call it `clean_transform`, rather than simply `clean`, so that it does not override any other targets for cleaning up that are included by the including Makefile.

Testsubject QA Makefile

This is an example of using a makefile to create quality assurance (QA) images, and then generate a final QA report in HTML using R Markdown.

The code for this example is in `testsubject/lib/makefiles/QA.mk`, and it is included by `testsubject/test001/Makefile`.

```
1 NIPYPATH=/usr/local/anaconda/bin
  FSL_DIR=/usr/share/fsl/5.0

2 .PHONY: TNSR MotionGraphs SkullstripQA QAReport

3 qa:   $(call print-help, qa, Create QA report) TSNR MotionGraphs SkullstripQA
     QAreport
```

1 As is customary in a makefile, we first define paths to locations that we want to refer to later on.
2 Here, our phony targets are targets that are not actual files.
3 This line tells the `make` help system what to do when you are unsure of what this makefile does. Here, it will print out what the `qa` target does (i.e., create a QA report). See Advanced Topics & Quality Assurance with R Markdown for more information about the help system.

```
4 TSNR: QA/images/rest_tsdiffana.gif

  QA/images/%_tsdiffana.gif: rest.nii.gz
      5  mkdir -p QA/images ;\
      6  pngout=`echo $@|sed 's/gif/png/g'` ;\
      7  $(NIPYPATH)/nipy_tsdiffana --out-file $$pngout $< ;\
         convert $$pngout $@ ;\
         rm -f $$pngout
```

4 Our first target creates TSNR images for the QA. In this example, the phony target TSNR only wants `make` to create a single `gif` image.
5 This line creates a directory called `QA/images` if it does not already exist. The `-p` flag tells `mkdir` not to throw an error if that directory exists, but create it if it does not. 6 The variable `pngout` is defined to take the filename of your target and substitute `gif` with `png`.
7 Subsequently, the code calls the script `nipy_tsdiffana` which is located in the directory `$(NIPYPATH)` you defined earlier.
The python script will generate a `png` image comprised of 4 graphs showing the scaled variance, slice-by-slice variance, scaled mean voxel intensity and the max/mean/min slice variation of your resting-state time series, as seen in the image below.

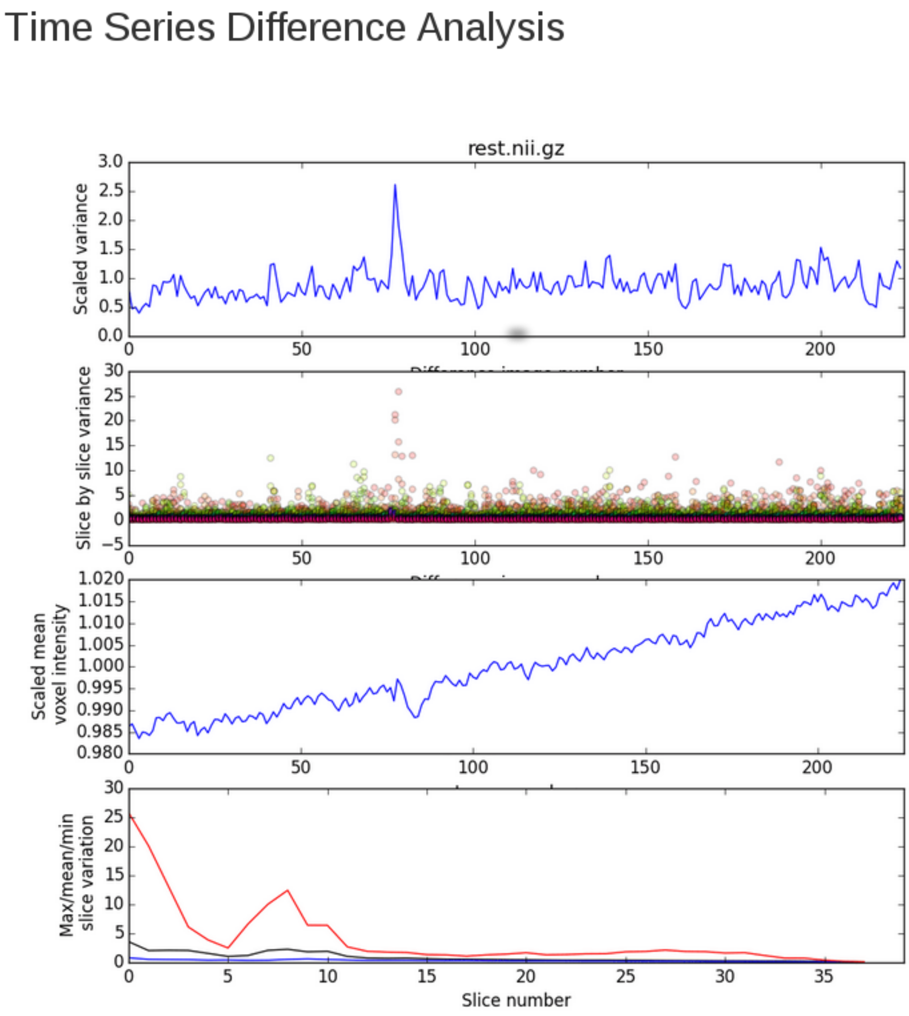


```
MotionGraphs: QA/images/rest_MotionGraphRotations.gif

QA/images/rest_MotionGraphRotations.gif: rest_dir/rest_mc.nii.gz
    8  $(PROJECT_HOME)/bin/R/MakingGraphs.Rscript  QA/images rest
```

8 `MakingGraphs.Rscript` is a R script that will generate 4 separate graphs for you:

1. A motion rotations graph showing rotations along the x/y/z planes.
2. A motion translations graph showing translations along the x/y/z planes.
3. A framewise displacement (FD) graph to show displacement in mm across acquired volumes.
4. A signal intensity (DVARS) graph to show signal intensity across acquired volumes.

To understand the usage of an R script, it is usually necessary to look at the code itself. In this line, the R script called with the output directory `QA/images` as the first argument, followed by the prefix `rest` to be used for naming the output images.

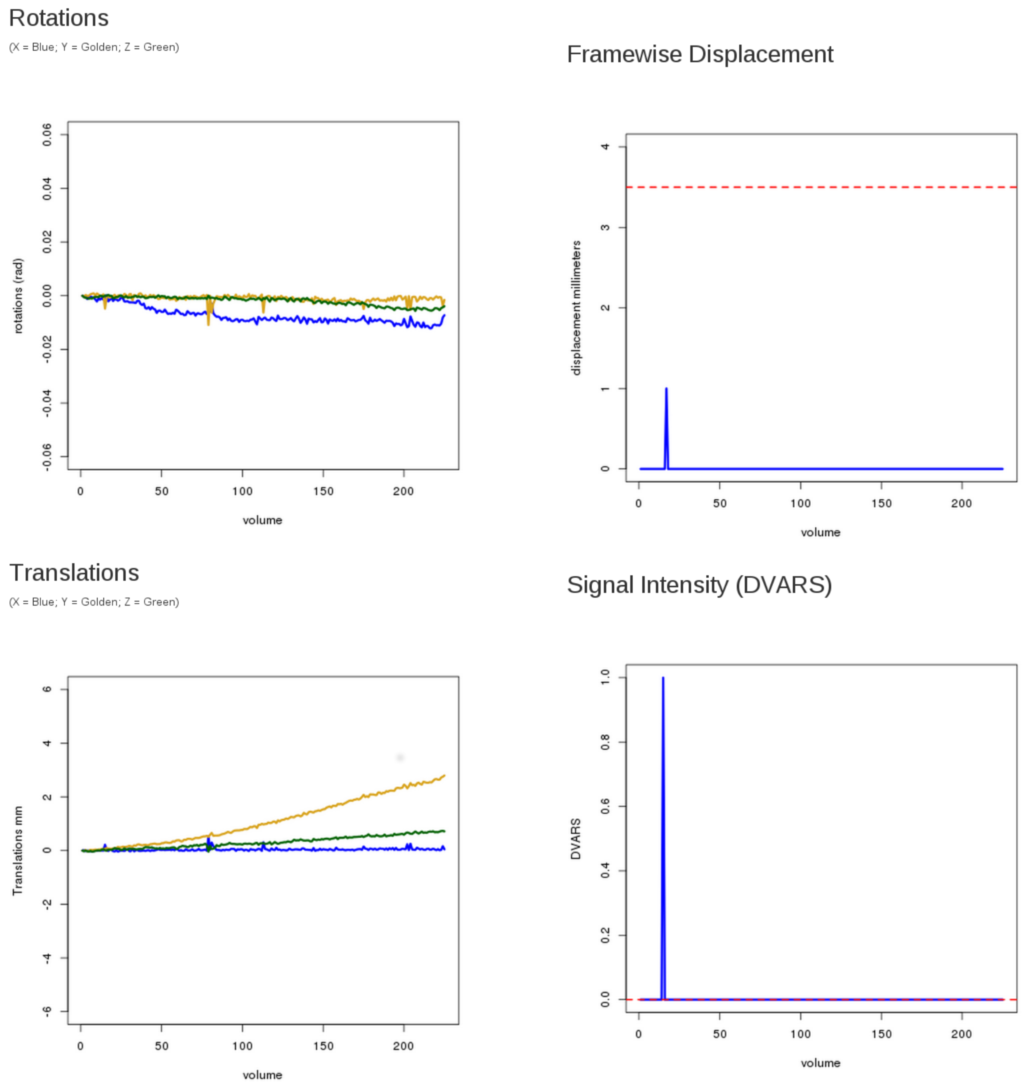


```
SkullstripQA: QA/images/T1_skstrip.gif

QA/images/T1_skstrip.gif: T1.nii.gz T1_skstrip_mask.nii.gz
        mkdir -p QA/images ;\
    9   $(FSL_DIR)/bin/overlay 1 1 $< -a $(word 2,$^) 1 10 \
        rendered_T1_brain.nii.gz ;\
   10   $(PROJECT_HOME)/bin/slices rendered_T1_brain.nii.gz \
        -o `dirname $@`/`basename $@ .gif`.png ;\
   11   convert `dirname $@`/`basename $@ .gif`.png -resize 500 $@ ;\
        rm rendered_T1_brain.nii.gz ;\
        rm `dirname $@`/`basename $@ .gif`.png
```

To ensure that our skull-strip does not remove too much of the brain or too little of the skull, we can create an image to overlay the skull-stripped mask generated from `resting.mk` on top of the T1 brain.

9 FSL `overlay` is a tool that is used to overlay a 3D images over another. It is capable of overlaying a maximum of 2 images on top of a reference image. Type `overlay` into your command line to understand how it is used. In this line, we call `overlay`. The `1`s that are provided as arguments specify the color and output type of your overlay. The next argument is the background image, which in this case is the first dependency we have listed, i.e. `T1.nii.gz`. The $^ refers to this file. The final output image is called `rendered_T1_brain.nii.gz`. Again, to understand the flags, you must look at overlay's usage.

10 FSL `slices` is a script that calls the FSL tool `slicer` to create an image consisting of 3 axial, 3 sagittal

and 3 coronal slices. Here, we feed it the `rendered_T1_brain.nii.gz` file that we want FSL `slices` to use. The output will be a file called `QA/images/T1_skstrip.png`. Instead of typing out the full name of the file, however, we simply provide the directory name and basename of our target and replace `.gif` with `.png`.
11 Following this, we convert our PNG image into a GIF image, using ImageMagick's `convert` that performs the conversion and resizes the image.[1]

T1 Skull-Strip

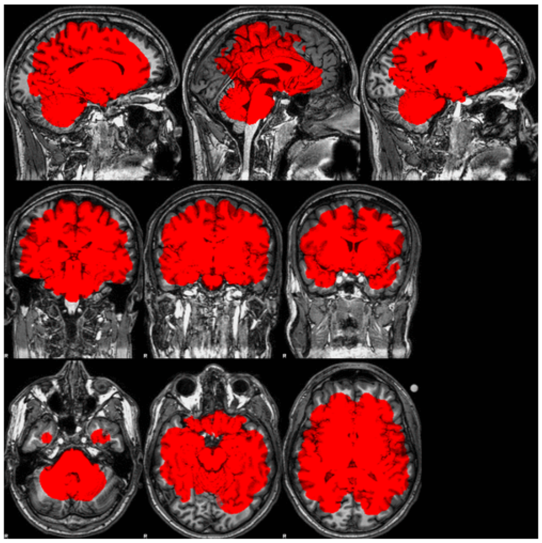

```
QAreport: QA/rest_Preprocessing.html

QA/rest_Preprocessing.html: $(PROJECT_HOME)/lib/Rmd/fMRI.Rmd TSNR MotionGraphs
    SkullstripQA
    12   sed -e 's/SUBJECT/$(subject)/g' -e 's/TASK/rest/g' $(word 1,$^) > \
         QA/rest_Preprocessing.Rmd ;\
    13   R -e 'library("rmarkdown"); \
         rmarkdown::render("QA/rest_Preprocessing.Rmd")' ;\
         rm -f QA/rest_Preprocessing.Rmd QA/rest_Preprocessing.md
```

Finally, to generate our QA HTML report, we use R Markdown. We do this by writing a file called `fMRI.Rmd`, which is the first dependency listed here. The `fMRI.Rmd` file reads the QA images that were generated in the previous portions of this makefile to create a HTML page (see Advanced Topics & Quality Assurance with R Markdown for a brief explanation of what goes into a Rmd file and how to write one).
12 In this line, we substitute pattern strings in the `fMRI.Rmd` file with variables that have been defined in the makefiles by using `sed`. `SUBJECT`, for instance, will be replaced with the `$(subject)` variable defined in the main makefile `$(PROJECT_HOME)/test001/Makefile`. `TASK` will be replaced with `rest`, because we are only interested in looking at the QA report for resting-state functional scans for now. We can define another variable called `task` if we have several types of runs that we want to generate QA reports for (e.g., task runs or multiple resting state runs). Once the pattern strings have been substituted, the `fMRI.Rmd` file will be copied over to the `QA` directory and be renamed as `rest_Preprocessing.Rmd`.
13 This line tells R to load the '`rmarkdown`' library so that it can read the R Markdown file. R will then render `QA/rest_Preprocessing.Rmd` to create your HTML report!

To view the full report, you can open the file `testsubject/test001/QA/rest_Preprocessing_example.html` in a browser.

[1] This step was necessary in earlier versions of R Markdown that had trouble including PNG images, but may not be necessary for you.

```
clean_qa:
rm -rf QA
```

Finally, with other makefiles, we define a `clean` target specifically for QA. Because quality assurance is an intermediate step in the neuroimaging processing pipeline, we do not necessarily need to retain the QA images and reports once they have been checked.

Example 6

Preprocessing Resting State Data

Benjamin A. Korman[1]

This is an example of how to use a makefile to preprocess functional resting state data. The pipeline incorporates typical preprocessing steps taken when preparing resting state data for analysis. This example includes motion and slice time acquisition correction, brain extraction, signal despiking, spatial smoothing and the calculation and removal of nuisance variables.

The code for this example is in `testsubject/lib/makefiles/resting.mk`.

```
❶ cwd=$(shell pwd)

SUBJECTS_DIR=$(PROJECT_HOME)/freesurfer
SCRIPTpath=$(PROJECT_HOME)/bin

FREESURFER_HOME=/usr/local/freesurfer/stable5_3

❷ HWHM=3
TR=2
SLICEORDER='ascending'
NPC=5
PCTVAR=0
erosionfactor=1
```

❶ In any script or makefile, it is a good idea to set working directory variables early on to ease the script writing process and to avoid later confusion. In this pipeline we have set a path variable for our subject subdirectory `cwd` as well as for the subdirectory that is home to the FreeSurfer pipeline's output `SUBJECTS_DIR`. In addition, we have set the variable `SCRIPTpath` to the subdirectory home of additional scripts that will be called during various preprocessing steps. ❷ Besides directory variables, it is helpful for us to set processing-relevant variables which can be used throughout the pipeline. Included here are variables for the half-width-half-maximum (`HWHM`) kernel size needed for spatially smoothing the resting state data, the time of repetition (`TR`) needed for slice time correction, as well as the number of principal components (`NPC`) and percent variance (`PCTVAR`) needed when using principal component analysis to calculate regressors for nuisance variables. Including these variables in the Makefile also makes it easy to extract them for inclusion in a generated QA report.

```
❸ .PHONY: rest  Freesurfer_test regressors Run_aCompCor postregression clean_rest
.SECONDARY:
```

❸ The targets listed in the `.PHONY` section are targets that do not correspond to actual files. Some of these targets (ex. `regressors`, `postregression`) are intended to be used to process the resting state data in grouped steps whereas other `.PHONY` targets are related to cleanup or testing. One such target is `clean_rest`

[1] bkorman@uw.edu

which may be called on to eliminate all of the pipeline's generated output when testing and debugging the pipeline.

The `.PHONY` target `rest` is used here to easily invoke all preprocessing steps of the pipeline. This is the target we would use to completely preprocess a subject. It is documented using the help system (see Example 4) and depends upon all preprocessing targets, described below.

```
❹ rest_dir/rest.nii.gz: rest.nii.gz
    ❺ echo "Setting up resting-state specific directory"
      mkdir -p rest_dir ;\
      if [ ! -h rest_dir/rest.nii.gz ];\
      then \
      ln -s $(cwd)/rest.nii.gz rest_dir/rest.nii.gz;\
      fi;\
      if [ ! -h rest_dir/rest.nii.gz ];\
      then \
      ln -s $(cwd)/T1.nii.gz rest_dir/T1.nii.gz;\
      fi
```

❹ This target creates resting state specific subdirectory to keep our project directory clean and organized. We first create `rest_dir` and then create symbolic links within this directory to the files needed for preprocessing. The required files for resting state preprocessing in this pipeline include the subject's resting state NIfTI image and its corresponding T1 anatomical image. ❺ Before we do this, we echo (i.e., print to the screen) an informative message to provide progress information while the pipeline runs. Similar informational messages are distributed throughout the remainder of the makefile.

```
rest_dir/rest_mc.nii.gz: rest_dir/rest.nii.gz
        echo "Preprocessing RESTING scans..." ;\
        echo "Beginning motion correction for rest.nii.gz using 4dRegister" ;\
    ❻ $(SCRIPTpath)/4dRegister.py --inputs $^ --tr $(TR) \
        --slice_order $(SLICEORDER) --time_interp True
```

Our first preprocessing step once a resting state-specific subdirectory has been created is to correct the resting state data for signal changes caused by motion and slice time acquisition. To do this we require the unprocessed resting state data, which is the sole dependency in this recipe. ❻ We will use `4dRegister.py`, a script provided by the Neuroimaging in Python (NIPY) community, to apply 4D motion and slice time correction simultaneously. The dependency `rest_dir/rest.nii.gz`, our input, is referred to as `$^` for short in the recipe. The resulting output will renamed with the `_mc` extension to indicate that it has been motion corrected.

```
❼ rest_dir/rest_bet.nii.gz: rest_dir/rest_mc.nii.gz
        echo "Skull stripping resting data with FSL's BET" ;\
        bet $< $@ -F
```

❼ Following motion and slice time correction, we invoke FSL's BET with the `-F` flag to skullstrip the 4D resting state data. The output, referred to as `$@` for short in the recipe, will be renamed `_bet` to indicate that it has undergone brain extraction (aka skull stripping).

```
rest_dir/rest_despike.nii.gz: rest_dir/rest_bet.nii.gz
        echo "Despiking the functional data with AFNI" ;\
    ❽ 3dDespike -ssave spikiness -q $^ ;\
        3dAFNItoNIFTI despike+orig.BRIK ;\
        3dAFNItoNIFTI spikiness+orig.BRIK ;\
        echo "Renaming output, zipping it and deleting unnecessary despike files"
           ;\
    ❾ mv despike.nii rest_dir/rest_despike.nii ;\
        mv spikiness.nii rest_dir/rest_spikiness.nii ;\
        rm -rf rest_dir/rest_despike.nii.gz rest_dir/rest_spikiness.nii.gz ;\
```

```
        gzip rest_dir/rest_despike.nii ;\
        gzip rest_dir/rest_spikiness.nii ;\
        rm -f despike+orig* ;\
        rm -f spikiness+orig*
```

8 After skullstripping the brain, we perform voxel-wise despiking with AFNI to reduce noise caused by framewise displacement. 9 In addition to our despiked resting state output, a list of "spikiness" values is produced. These are renamed to `rest_despike.nii.gz` and `rest_spikiness.nii.gz` respectively. These output files are then compressed to save space while the original output files are removed.

```
10 rest_dir/rest_ssmooth.nii.gz: rest_dir/rest_despike.nii.gz
        echo "Smoothing resting date with FSL's SUSAN" ;\
        susan $^ -1.0 $(HWHM) 3 1 0 $@
```

10 Once the resting state data has been despiked it is ready to be spatially smoothed. To do this we use FSL's SUSAN with a 3D smoothing kernel 3mm in size. We rename our output with the `_ssmooth` extension to indicate that it has been spatially smoothed.

```
11 Freesurfer_test: $(SUBJECTS_DIR)/$(subject)/mri/brainmask.mgz
                $(SUBJECTS_DIR)/$(subject)/mri/aparc+aseg.mgz

12 xfm_dir/rest_to_freesurfer.mat: rest_dir/rest_despike.nii.gz
                        $(SUBJECTS_DIR)/$(subject)/mri/aparc+aseg.mgz
        mkdir -p xfm_dir ;\
        source /usr/local/freesurfer/stable5_3/SetUpFreeSurfer.sh ;\
        export SUBJECTS_DIR=$(SUBJECTS_DIR) ;\
        fslroi rest_dir/rest_despike.nii.gz rest_dir/rest_despike_vol0 0 1 ;\
        bbregister --s $(subject) --mov rest_dir/rest_despike_vol0.nii.gz \
        --reg xfm_dir/rest_to_freesurfer.dat --init-fsl --bold \
        --o xfm_dir/rest_to_freesurfer.nii.gz \
        --fslmat xfm_dir/rest_to_freesurfer.mat

13 xfm_dir/freesurfer_to_rest.mat: xfm_dir/rest_to_freesurfer.mat
                convert_xfm -omat $@ -inverse $<
```

11 To proceed with the calculation and removal of nuisance regressors from our spatially smoothed resting state data we first need to check whether the necessary FreeSurfer output files are available. This is because our estimation of background noise in the data will require white matter and cerebrospinal fluid masks which are created from the output of FreeSurfer's `recon-all` segmentation process. We normally run FreeSurfer separately, because it takes a lot longer to run than the resting state preprocessing. This target depends upon the FreeSurfer results `brainmask.mgz` and `aparc+aseg.mgz`. If these do not exist, no processing that depends upon this target can occur. Because the target `Freesurfer_test` is not an existing file (and will not at any point be created) it is included in the `.PHONY` section located above.

12 Once we are certain that the necessary files exist in our FreeSurfer directory, we create a directory (`xfm_dir`) in which to keep all of our data transformations. Then, using `fslroi`, we select the first volume of our despiked resting state data (`rest_despike_vol0`) as input for FreeSurfer's `bbregister`. BBregister is used to perform within-subject, cross-modal rigid registration. 13 After registering the despiked data in FreeSurfer space we must transform this registration back into resting state space. This is because FreeSurfer space is unique and the pipeline will encounter problems if attempting to directly register images from FreeSurfer space to standard (i.e. MNI) space.

```
14 regressors: rest_dir/fs_wMmask.nii.gz rest_dir/fs_csf_mask.nii.gz
                                rest_dir/rest_wm.nii.gz rest_dir/rest_csf.nii.gz

15 rest_dir/fs_wm_mask.nii.gz: $(SUBJECTS_DIR)/$(subject)/mri/aparc+aseg.mgz
        source /usr/local/freesurfer/stable5_3/SetUpFreeSurfer.sh ;\
```

```
        export SUBJECTS_DIR=$(SUBJECTS_DIR) ;\
        mri_binarize --i $^ --o $@ --erode 1 --wm

rest_dir/fs_csf_mask.nii.gz: $(PROJHOME)/freesurfer/$(subject)/mri/aparc+aseg.mgz
        source /usr/local/freesurfer/stable5_3/SetUpFreeSurfer.sh ;\
        export SUBJECTS_DIR=$(SUBJECTS_DIR) ;\
        mri_binarize --i $^ --o $@ --erode 1 --ventricles

16 rest_dir/rest_wm.nii.gz: rest_dir/fs_wm_mask.nii.gz rest_dir/rest_despike.nii.gz
                        xfm_dir/freesurfer_to_rest.mat
        flirt  -ref $(word 2,$^) -in $(word 1,$^) -out $@ -applyxfm \
        -init $(word 3,$^) ;\
        fslmaths $@ -thr .5 -bin $@

rest_dir/rest_csf.nii.gz: rest_dir/fs_csf_mask.nii.gz rest_dir/rest_despike.nii.gz
                        xfm_dir/freesurfer_to_rest.mat
        flirt  -ref $(word 2,$^) -in $(word 1,$^) -out $@ -applyxfm \
        -init $(word 3,$^) ;\
        fslmaths $@ -thr .3 -bin $@
```

14 The target `regressors` is included here for ease of use if one wanted to specifically create or recreate binarized masks from FreeSurfer's white matter and ventricle segmentations. 15 We binarize and extract white matter and ventricle masks. 16 We register the despiked resting state data to the white matter and CSF masks using FSL's FLIRT. Note that in the recipe the `$(word #, $^)` refers to a dependency in the dependency list. The first dependency may be referenced in the recipe as `$(word 1, $^)`, the second `$(word 2, $^)`, and so forth.

```
17 Run_aCompCor: rest_dir/rest_wm.txt rest_dir/rest_csf.txt

rest_dir/rest_wm.txt: rest_dir/rest_despike.nii.gz rest_dir/rest_wm.nii.gz
        fslmaths rest_dir/rest_despike.nii.gz -mas rest_dir/rest_wm.nii.gz
           rest_dir/rest_wm_ts.nii.gz ;\
   18   Rscript  $(SCRIPTpath)/aCompCor.R rest_dir/rest_wm_ts.nii.gz $(NPC) $(
           PCTVAR) ;\
        convert rest_dir/rest_wm_ts_VarianceExplained.png \
        rest_dir/rest_wm_ts_VarianceExplained.gif ;\
        rm rest_dir/rest_wm_ts_VarianceExplained.png ;\
        mv rest_dir/rest_wm_ts_pc.txt $@

rest_dir/rest_csf.txt: rest_dir/rest_despike.nii.gz rest_dir/rest_csf.nii.gz
        fslmaths rest_dir/rest_despike.nii.gz -mas rest_dir/rest_csf.nii.gz
           rest_dir/rest_csf_ts.nii.gz ;\
        Rscript  $(SCRIPTpath)/aCompCor.R rest_dir/rest_csf_ts.nii.gz $(NPC) \
        $(PCTVAR) ;\
        convert rest_dir/rest_csf_ts_VarianceExplained.png \
        rest_dir/rest_csf_ts_VarianceExplained.gif ;\
        rm rest_dir/rest_csf_ts_VarianceExplained.png ;\
        mv rest_dir/rest_csf_ts_pc.txt $@

19 rest_dir/rest_abs_pc1.txt: rest_dir/rest_mc.nii.gz
        echo "Creating list of motion regressors using MotionRegressorGenerator.py
           " ;\
        $(SCRIPTpath)/MotionRegressorGenerator.py -i rest_dir/rest.par \
        -o rest_dir/rest

20 rest_dir/rest_nuisance_regressors.txt: rest_dir/rest_csf.txt rest_dir/rest_wm.txt
```

```
    rest_dir/rest_abs_pc1.txt
        echo "Combine lists of motion regressors into one text file" ;\
        paste $(word 1,$^) $(word 2,$^) $(word 3,$^) rest_dir/rest_rel_pc1.txt \
        > $@
```

Once we have registered the despiked resting state data to the white matter and CSF masks, we need to compute nuisance regressors. **17** The target `Run_aCompCor` depends upon these regressors and is a convenience target. **18** The R script `aCompCor` calculate nuisance regressors from the masked resting state data. This script can extract a set number of principal components `NPC` or by setting a specific percent of variance `PCTVAR` that should be explained by the principal components included in the analysis. In this example we use 5 principal components. **19** A python script named `MotionRegressorGenerator`, located in the `bin` directory, is also called in this pipeline to calculate nuisance regressors from motion parameters. **20** The nuisance regressors generated during this step are then combined with the nuisance regressors calculated by `aCompCor` into a single text file.

```
21 postregression: rest_dir/rest_designrange.txt rest_dir/rest_postregression.nii.gz
        echo "Regressing motion artifacts out of resting-state data"

22 rest_dir/rest_designrange.txt: rest_dir/rest_nuisance_regressors.txt
        Rscript $(SCRIPTpath)/rangeArray.R $< $@

23 rest_dir/rest_postregression.nii.gz: rest_dir/rest_ssmooth.nii.gz rest_dir/
    rest_nuisance_regressors.txt rest_dir/rest_designrange.txt
        npc=`awk '{print NF}' $(word 2, $^) | sort -nu | tail -n 1` ;\
        npts=`wc -l $(word 2, $^)` ;\
        echo "/NumWaves $$npc" >  rest_dir/rest_regressors.feat.mat ;\
        echo "/NumPoints $$npts" >> rest_dir/rest_regressors.feat.mat ;\
        echo "/PPheights " `cat $(word 3, $^)` >> \
        rest_dir/rest_regressors.feat.mat ;\
        echo "/Matrix" >> rest_dir/rest_regressors.feat.mat ;\
        cat $(word 2, $^) >> rest_dir/rest_regressors.feat.mat ;\
        fsl_regfilt -i  rest_dir/rest_ssmooth.nii.gz \
        -d rest_dir/rest_regressors.feat.mat  \
        -f `seq -s, 1 1 $$npc` -o $@

24 rest_dir/rest_outliers_dvars_vals.txt: rest_dir/rest_mc.nii.gz
                $(PROJECT_HOME)/bin/motion_outliers -i $^ \
                -o rest_dir/rest_outliers_dvars.txt \
                -s rest_outliers_dvars_vals.txt --dvars --nomoco

rest_dir/rest_outliers_fd_vals.txt: rest.nii.gz
                $(PROJECT_HOME)/bin/motion_outliers -i $^ \
                -o rest_dir/rest_outliers_fd.txt \
                -s rest_outliers_fd_vals.txt --fd
```

Once the nuisance regressors have been calculated and combined into a single file, the next step is to remove them from the data. **21** This portion of the pipeline may be called specifically using the target `postregression`. **22** To regress out nuisance variables, we create a little FEAT design matrix and run `fsl_regfilt`. We calculate the range of the included nuisance regressors using the R script `rangeArray.R`. **23** Finally, we denoise the data by regressing out the nuisance regressors using simple ordinary least squares regression with FSL. **24** It may also be helpful to create text files listing motion outlier volumes based on dvars (RMS intensity differences in images between timepoints) or framewise displacement (fd).

```
clean_rest:
        echo "Removing all output files except for freesurfer transforms" ;\
        rm -rf rest_dir
```

Lastly, we create a target to remove all files created by the pipeline. This is particularly useful when testing and debugging the pipeline. When called, this target will simply remove the `rest_dir` directory and all files located within it; in other words, everything that we have created with this Makefile.

Example 7

Generating A Methods Section

Benjamin A. Korman[1]

This is an example of how to use a makefile to automatically generate a methods section. Using a makefile to generate methods sections saves time and limits human error when reporting MRI acquisition and preprocessing parameters by obtaining the necessary values directly from the image source data, the computer system, and the processing pipelines themselves. This is an example of how we handle provenance — we generate a human-readable description of the pipelines while saving information about the versions of the programs used to generate them.

We do this in two steps. First, important parameters are written to a comma-separated value (CSV) file. These are extracted from wherever appropriate for the study using a `bash` shell script. For example, versions of tools such as FSL and AFNI can be obtained from the system. Imaging acquisition parameters can be obtained from the PAR file (a Philips human-readable parameter file), or the DICOMs, or any other exported parameter specification file appropriate to your study. Parameters that are used in the processing pipelines themselves can be obtained from those makefiles. After the CSV file is complete, we render an R Markdown file that loads those values and generates an HTML report. We include PubMed links to references in the R Markdown file so that, if necessary, the links can be used to import bibliographic records into bibliographic software databases.

The code for this example is in `testsubject/lib/makefiles/methodsgenerator.mk`. This example builds upon the other processing pipelines in place for `testsubject`, `dti_susceptibility_correction`, and `ASLTestsubject`. It describes the resting state processing pipeline in Preprocessing Resting State Data, the DTI susceptibility-correction pipeline in DTI Distortion Correction with Conditionals, and the ASL processing pipeline in Quantifying Arterial Spin Labeling Data.

1
```
rs_parsource=$(wildcard parrecs/*REST*.PAR)
t1_parsource=$(wildcard parrecs/*MPRAGE*.PAR)
dti_parsource=$(wildcard parrecs/*DTI*.PAR)
asl_parsource=$(wildcard parrecs/*ASL*.PAR)
```

This makefile begins by 1 searching for and selecting the PAR source files from which the makefile will acquire the majority of the generated methods section's parameter values. Because the naming conventions for the PAR files do not often follow a specific pattern, we use a wildcard to search for a string we are sure will be in the file name.

2
```
rs_framewisedisplacement="\#"
rs_accelerationfactor="\#"
t1_shotinterval="\#"
dti_accelerationfactor="\#"
asl_invertpulse1="\#"
asl_invertpulse2="\#"
asl_labelduration="\#"
```

[1] bkorman@uw.edu

```
asl_postlabeldelay="\#"
asl_M0_repetitiontime="\#"
```

❷ Not all of the necessary parameter values are available in PAR files, so there are few parameters that must be set (by replacing the #'s specified for these values). Here we wish to report framewise displacement and acceleration factor used during resting-state data acquisition as well as the shot interval during the T1 anatomical scan acquisition along with parameters important for diffusion tensor imaging (DTI) and arterial spin labeling (ASL) processing. This makefile currently reports parameters important to resting-state, DTI, and ASL data and preprocessing but may be expanded to include other MRI techniques as well (e.g. voxel-based morphometry and susceptibility-weighted imaging).

```
❸ .PHONY: provenance clean_provenance
.SECONDARY:

❹ provenance: $(call print-help,provenance,"Automatically generate a methods section
    ") provenancedir provenance/parameter_table.csv provenance/Methods_Generator.
    html
```

❸ The targets listed in the `.PHONY` section are targets that do not correspond to actual files. The target ❹ `provenance` may be called to undertake all necessary steps towards creating an automated methods section while the target `clean_provenance` may be invoked to eliminate all of the makefile's generated output, useful for testing and debugging purposes.

```
❺ provenancedir:
        echo "Creating provenance directory" ;\
        mkdir -p provenance

❻ provenance/parameter_table.csv: provenancedir $(rs_parsource) $(t1_parsource) $(
    dti_parsource) $(asl_parsource)
        echo "Creating parameter table" ;\
        bash $(PROJECT_HOME)/bin/Methods_Generator.sh  $(rs_parsource) $(
           t1_parsource) $(dti_parsource) $(asl_parsource) $(
           rs_framewisedisplacement) $(rs_accelerationfactor) $(t1_shotinterval) $
           (dti_accelerationfactor) $(asl_invertpulse1) $(asl_invertpulse2) $(
           asl_labelduration) $(asl_postlabeldelay) $(asl_M0_repetitiontime) > $@
```

❺ This target creates a data provenance subdirectory to keep our project directory clean and organized. The files generated by this makefile will be stored in this folder. ❻ Once a methods specific subdirectory has been created it is time to extract the parameter values we wish to include in our methods section from their respective PAR files and tabulate them appropriately. This is done by ❼ calling a `bash` script which generates a table including the parameters' name (both an abbreviated form as well as a long form), value, and unit of measurement if applicable.

```
provenance/Methods_Generator.html: provenance/parameter_table.csv
        echo "Creating methods section html file" ;\
        cd provenance ;\
        cp $(PROJECT_HOME)/lib/Rmd/Methods_Generator.Rmd . ;\
        Rscript -e ‘library("rmarkdown"); \
        rmarkdown::render("Methods_Generator.Rmd")’
```

Using the `parameter_table.csv` file created in the previous step, an R script is called to incorporate the necessary parameter values into a model methods section R Markdown (.Rmd) file. One thing that is a little frustrating about R Markdown is that it will look for files in the directory in which it is located. This is why we first copy it to the `provenance` subdirectory. The R Markdown file then looks for the CSV file in the current working directory, with no need to pass paths back and forth between programs.

The outputted methods section HTML file can be viewed in any web browser. Figure 7.1 shows the R Markdown code that is processed (on the left) to generate the HTML report (on the right). The R Markdown

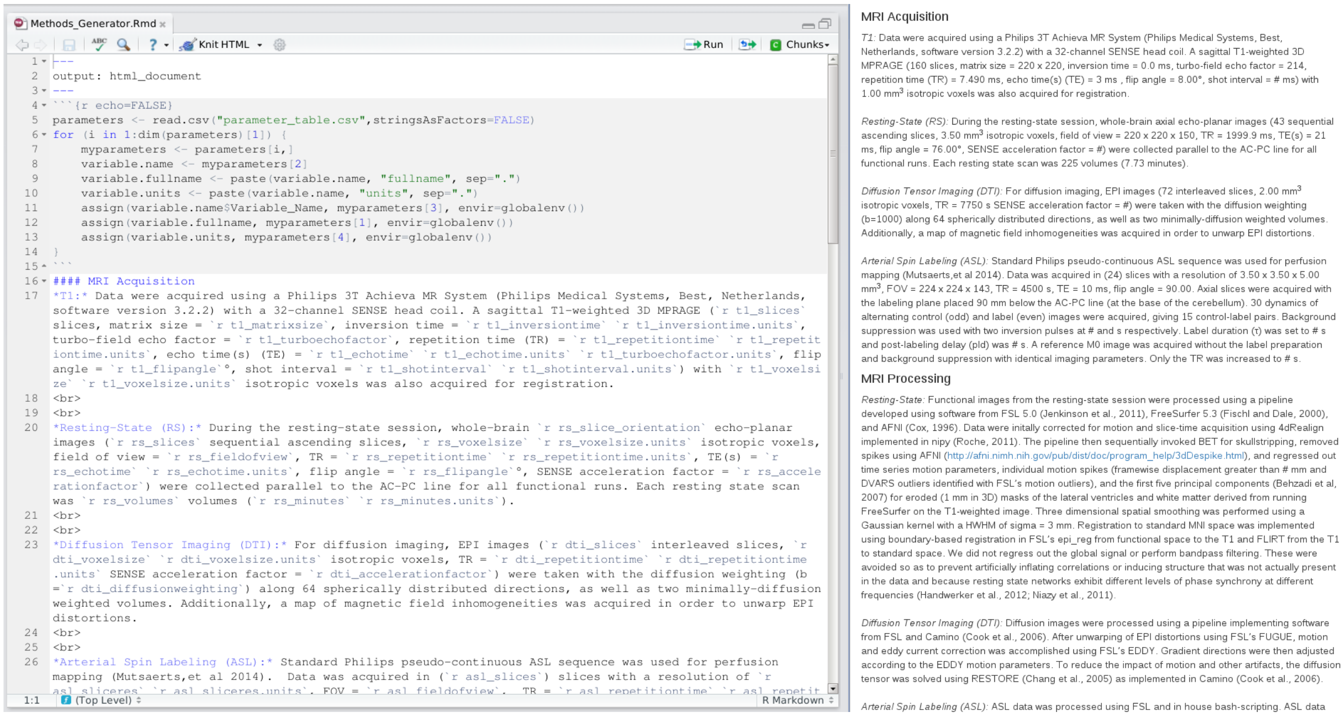

Figure 7.1: Methods generation in R Markdown

code is very simple. The first "chunk" of code reads in the CSV file and uses the short variable names to construct a variety of variables for the value of the parameter, its full name, and the units. These are accessed in the text description of the protocol with the syntax `'r variablename'`. For more information about how to write reports with R Markdown, see their excellent documentation.

```
clean_provenance:
        echo "Removing provenance directory" ;\
        rm -r provenance
```

Lastly, we create a target to remove all files created by this makefile. When called, this target will simply remove the `provenance` directory and all files located within it.

Example 8

Seed-based Functional Connectivity Analysis I

Susan J. Melhorn[1]

This is an example of how to use a makefile to execute a single-subject seed-based functional connectivity analysis. Here, the network of interest is the salience network with a 4mm sphere in the right insula as the seed region (`seedrins4.nii.gz`). Note the seed region mask has already been created based on the literature (Seeley WW (2008); Lee SE (2014)).

We conduct a seed-based analysis by (1) extracting the time course of interest, and (2) using this time course as a regressor in a GLM, including all nuisance variables. Here, this analysis is implemented using FSL's FEAT (`feat`) program.

The code for this example is in `testsubject/lib/makefiles/fcconnectivity.mk`. Note that the variables `PROJECT_HOME, FSL_DIR, STD_BRAIN, and subject` are all set elsewhere, but are needed. This example builds upon the other processing pipelines in place for `testsubject`.

```
.PHONY: Connectivity clean_fcconnectivity

Connectivity: $(call print-help,Connectivity,"Perform subject-level seed-based
   connectivity analysis") \
fcconnectivity_dir/Rins_in_func.nii.gz fcconnectivity_dir/Rins_ts.txt \
fcconnectivity_dir/$(subject).feat/stats/zstat1.nii.gz \
fcconnectivity_dir/$(subject).feat/stats/FZT1.nii.gz

❶ fcconnectivity_dir/Rins_in_func.nii.gz: $(PROJECT_HOME)/lib/fcconnectivity/
   seedrins4.nii.gz xfm_dir/MNI_to_rest.mat rest_dir/rest_ssmooth.nii.gz
        mkdir -p fcconnectivity_dir ;\
        flirt -in $(PROJECT_HOME)/lib/fcconnectivity/seedrins4.nii.gz \
        -ref rest_dir/rest_ssmooth.nii.gz -applyxfm \
        -init xfm_dir/MNI_to_rest.mat -out $@ ;\
        fslmaths $@  -bin $@

❷ fcconnectivity_dir/Rins_ts.txt: fcconnectivity_dir/Rins_in_func.nii.gz
                        rest_dir/rest_ssmooth.nii.gz
        mkdir -p `dirname $@ ` ;\
        fslmeants -i rest_dir/rest_ssmooth.nii.gz -o $@ \
        -m fcconnectivity_dir/Rins_in_func.nii.gz
```

❶ This target puts the seed mask in functional space from standard space. Note the dependencies here are created in the resting state preprocessing makefile. Normally we would threshold the mask at .5 to preserve its

[1] smelhorn@uw.edu

volume, but this particular seed is so small that we only binarize it using fslmaths 2 We use fslmeants to extract the timeseries from the masked functional connectivity data.

3
```
fcconnectivity_dir/fcconnectivity.fsf: $(PROJECT_HOME)/lib/fcconnectivity/
   fcconnectivityTEMPLATE.fsf
        pipelines=`echo $(MAKEPIPELINES)|sed 's/\//\\\\\//g'`;\
        sed -e 's/SUBJECT/$(subject)/g' \
        -e 's/MAKEPIPELINES/'$$pipelines'/g'  $< > $@
```

This step relies on a template .fsf file created using the Feat graphical user interface. We set this up for the first subject, and use the text file for the seed time course as an explanatory variable. The input data is the resting state data that has been processed up until nuisance regressors are removed. We use no convolution (the seed time course is already convolved by the brain) and turn off temporal derivative and temporal filtering. We include all nuisance regressors in the model.

The .fsf file created in this way has hardcoded paths that include the directory of the specific subject and the path to all of the pipeline examples. To make it work for other subjects (at other sites) we parameterize the file created by Feat to substitute the path to the subject with the text MAKEPIPELINES, and the subject identifier with the text SUBJECT. You can see this file at lib/fcconnectivity/fcconnectivityTEMPLATE.fsf. We use the sed command to replace these placeholder strings with their actual values, thus generating a customized .fsf file for each subject. We set the pipelines variable just to escape the slashes (/) in the MAKEPIPELINES variable for sed. If you like, after you run this step, you can open up the newly created .fsf file to see the settings that are described above.

```
fcconnectivity_dir/$(subject).feat/stats/zstat1.nii.gz:
rest_dir/rest_ssmooth.nii.gz rest_dir/rest_nuisance_regressors.txt \
fcconnectivity_dir/Rins_ts.txt fcconnectivity_dir/fcconnectivity.fsf
        feat fcconnectivity_dir/fcconnectivity.fsf
```

Run feat. Note that there are several dependencies in this rule that don't appear anywhere else in the recipe. However, they are needed by the .fsf file used to run feat.

```
fcconnectivity_dir/$(subject).feat/stats/FZT1.nii.gz:
fcconnectivity_dir/$(subject).feat/stats/zstat1.nii.gz \
rest_dir/rest_ssmooth.nii.gz rest_dir/rest_nuisance_regressors.txt
        NBVOLS='fslval rest_dir/rest_ssmooth.nii.gz dim4';\
        NBNUISANCE=`awk '{print NF}' rest_dir/rest_nuisance_regressors.txt \
        |head -1` ;\
        SD=`echo "sqrt($$NBVOLS-$$NBNUISANCE-3)" | bc -l` ;\
        echo $$SD ;\
        fslmaths fcconnectivity_dir/$(subject).feat/stats/zstat1.nii.gz \
        -div $$SD fcconnectivity_dir/$(subject).feat/stats/FZT1.nii.gz
```

After running feat, we need to convert the zstat to Fisher's Z transformed partial correlation. This math is implemented using fslmaths and is based on Jeanette Mumford's recommendation.

```
clean_fcconnectivity:
        rm -rf fcconnectivity_dir
```

Finally, we define a target to remove all the files we have created.

Example 9

Seed-based Functional Connectivity Analysis II

Matthew Peverill[1]

This is an example of how to use `make` to preprocess and analyze resting state fMRI data and conduct a group-level seed-based functional connectivity analysis. The fMRI data is motion corrected using Nipype (Gorgolewski et al., 2011) and then skull stripped and smoothed using FSL (Jenkinson et al., 2012) standard utilities. FreeSurfer is used to structurally define a region of interest (ROI - for the purpose of this example, the right superior frontal gyrus) specific to each subject which is then registered to the functional image.[2] `fslmeants` is used to extract a timeseries of activity in this ROI which is entered as a regressor in FSL along with nuisance regressors corresponding to motion outliers, rigid body movement, and white matter and CSF fluctuations to correct for noise. A simple quality assurance (QA) report is generated for registration and ROI extraction using an HTML template.

The code for this example is in `$MAKEPIPELINES/tapping`, in multiple files described below.

Group Level Makefile - `tapping/Makefile`

```
1 SUBJECTS = $(wildcard TappingSubj?)
  .PHONY: makefiles all $(SUBJECTS) resting-gfeat

  all: makefiles $(SUBJECTS) resting-gfeat

  $(SUBJECTS):
2         $(MAKE) --directory=$@ subject-resting

3 makefiles: $(SUBJECTS:%=%/Makefile)

  $(SUBJECTS:%=%/Makefile): lib/resting/subject.mk
4         ln -s $$PWD/$< $@

  resting-gfeat: resting.gfeat/cope1.feat/stats/cope1.nii.gz

5 resting.gfeat/cope1.feat/stats/cope1.nii.gz: lib/resting/Tapping_SecondLevel.fsf
           $(SUBJECTS)
          rm -rf /project_space/makepipelines/tapping/resting.gfeat ;\
          feat $<
```

[1] mrpev@uw.edu

[2] This is a very large seed! However, this is just an example.

The group level Makefile in the project parent allows for recursive make of all subjects in the project.

1 The variable `SUBJECTS` is set to a wildcard which captures all folders named `TappingSubj` followed by a single character (this would need to be modified for more than nine subjects - likely by expanding all subject numbers to two digit format and adding a second `?`).

2 This recipe provides the definition that `make (subject)` should execute `make subject-resting` within each subject's folder after a subject-level `Makefile` has been generated.

3 The `makefiles` phony target uses `make`'s substitution reference syntax to expand the list of subjects to a list of subject level makefiles.

4 Another recipe provides rules to generate the makefiles enumerated in 3 .

5 The final recipe executes group-level feat according to the template `lib/resting/Tapping_SecondLevel.fsf`, generating the final results of the analysis. For convenience, this recipe can be run by typing `make resting-gfeat`.

Subject Level Makefile - `tapping/lib/resting/subject.mk`

The subject-level `Makefile` is stored in `lib/resting/`, but a symbolic link pointing to it is generated in every subject directory. This makefile sets up necessary environment variables and then imports all component makefiles. The code executes exactly as if everything were in one makefile, but separating the code by procedure makes the code more readable.

```
.PHONY: prept1 clean printall
.SECONDARY:

ifeq "$(origin MAKEPIPELINES)" "undefined"
MAKEPIPELINES=/project_space/makepipelines
endif

SHELL = /bin/bash
6  subject=$(shell pwd|egrep -o 'TappingSubj[0-9]*')

projdir=$(MAKEPIPELINES)/tapping/
7  SUBJECTS_DIR=$(projdir)/freesurfer
SCRIPTpath=$(projdir)/bin

8  FSL_DIR=/usr/share/fsl/5.0/bin
AFNIpath=/usr/lib/afni/bin

TR=2.5
```

When we run `make` within a subject directory, the program reads `Makefile` first. Because of this, we can set environment variables once in this file and they will carry over to all included files. This section should be carefully reviewed when moving the code to a new environment.

6 Here, the `subject` variable is set to the subject directory name.

7 FreeSurfer's `SUBJECTS_DIR` variable should be set for the users environment.

8 Paths to the local installation of FSL and AFNI are set here. Later references to programs provided by these packages use these variables to provide a path so that the pipeline can be flexibly executed with different package versions.

```
9  include $(projdir)/lib/resting/Preprocess.mk
include $(projdir)/lib/resting/Regressors.mk
include $(projdir)/lib/resting/timeseries.mk
include $(projdir)/lib/resting/feat.mk
include $(projdir)/lib/resting/qa.mk

10 subject-resting: prept1 prepfunc regressors timeseries feat qa-all
```

```
11  clean-resting:
            rm -rf resting memprage ;\
            rm -f QA-report.html

12  tidy-resting:
            rm -fv resting/* ;\
            rm -fv memprage/* ;\
            rm -rf resting/timeseries

13  print-%:
            @echo $* = $($*)
```

9 Here we import child makefiles which contain all of the code necessary to execute the analysis.

10 The phony target `subject-resting` depends on all components of the analysis, providing one target to execute the entire workflow for this subject. This is the target referenced in the group-level makefile.

11 The `clean-resting` target removes all files created by the pipeline, returning the subject folder to a pre-run state.

12 The `tidy-resting` target allows for the removal of working files only and retains feat output.

13 The `print-%` target is provided for debugging purposes, eg. `make print-subject` will return the value of the `subject` environment variable.

Preprocessing - `tapping/lib/resting/Preprocess.mk`

```
    .PHONY= prept1 prept1-qa prepfunc prepfunc-qa

    prept1: memprage/T1_brain.nii.gz

14  memprage/T1.nii.gz: MPRAGE_S2.nii.gz
15          mkdir -p memprage && \
            $(FSL_DIR)/bin/fslreorient2std $< $@

16  memprage/T1_brain.nii.gz: memprage/T1.nii.gz
            $(FSL_DIR)/bin/bet $< $@

17  memprage/T1_brain_mask.nii.gz: memprage/T1_brain.nii.gz
            $(FSL_DIR)/bin/fslmaths $< -bin $@
```

This code provides basic preprocessing of the T1 structural image. In preprocessing, each image is generated from the prior image, so the makefile reads much as a `bash` script would with each line proceeding from the last, save that dependency and target shortcuts are substituted for filenames.

14 `T1.nii.gz` is generated by orienting the scanner output image to FSL's preferred orientation using `fslreorient2std`.

15 The '`&& \`' syntax at line ends causes `bash` to stop and make to return a failure for the recipe if the preceding command fails (otherwise all commands are executed and only the success or failure of the last executed command is considered when `make` reports whether the recipe ran successfully). This saves time by skipping programs that cannot succeed and makes the cause of a failure more clear, since the last error message presented to the user will be from the command that first failed instead of from a program that failed as a consequence.

16 Skull stripping is accomplished with `bet`.

17 A mask of the skull stripped brain is generated with `fslmaths`.

```
prepfunc: xfm_dir/T1_to_Resting.mat resting/Resting_ssmooth.nii.gz
        xfm_dir/fs_to_Resting.mat

```

```
   resting/Resting_orient.nii.gz: Resting.nii.gz
           mkdir -p resting && \
           $(FSL_DIR)/bin/fslreorient2std $< $@

18 resting/Resting_mc.nii.gz: resting/Resting_orient.nii.gz
           python $(SCRIPTpath)/4dRegister.py --inputs $< --tr $(TR) \
           --slice_order 'ascending' && \
            mv resting/Resting_orient_mc.nii.gz $@

19 resting/Resting.par: resting/Resting_mc.nii.gz
           mv resting/Resting_orient.par $@

20 resting/Resting_brain.nii.gz: resting/Resting_mc.nii.gz
           $(FSL_DIR)/bin/fslroi $^ resting/Resting_vol0 0 1 && \
           $(FSL_DIR)/bin/bet resting/Resting_vol0 resting/Resting_vol0 -f 0.3 && \
           $(FSL_DIR)/bin/fslmaths resting/Resting_vol0 -bin resting/Resting_vol0 &&
               \
           $(FSL_DIR)/bin/fslmaths $^ -mas resting/Resting_vol0 $@ && \
           rm resting/Resting_vol0.nii.gz

21 resting/Resting_ssmooth.nii.gz: resting/Resting_brain.nii.gz
           $(FSL_DIR)/bin/susan $^ -1.0 3 3 1 0 $@
```

This code provides basic preprocessing of the functional resting state image.

18 A python script, `4dRegister.py`, is called which outputs both the motion corrected image and a `.par` file containing 6 motion regressors.

19 Although the par file is created by `4dRegister.py`, a separate recipe allows us to rename the file and, importantly, allows for the execution of later recipes which depend on the `.par` file.

20 `fslroi` is used to extract the first volume (time point) of the four dimensional functional image. `bet` Is used to skull-strip the image and a binarized mask is generated and applied using `fslmaths`.

21 Smoothing is applied with `susan` to produce the final preprocessed image.

```
22 xfm_dir/Resting_to_T1.mat: resting/Resting_brain.nii.gz memprage/T1.nii.gz
       memprage/T1_brain.nii.gz
           mkdir -p xfm_dir && \
           $(FSL_DIR)/bin/fslroi $< resting/resting_vol0 0 1 && \
           $(FSL_DIR)/bin/epi_reg --epi=resting/resting_vol0 --t1=$(word 2,$^) \
           --t1brain=$(word 3,$^) --out=xfm_dir/'basename $@ .mat'

23 xfm_dir/Resting_to_T1.nii.gz: xfm_dir/Resting_to_T1.mat

   xfm_dir/T1_to_Resting.mat: xfm_dir/Resting_to_T1.mat
           $(FSL_DIR)/bin/convert_xfm -omat $@ -inverse $^

24 $(SUBJECTS_DIR)/$(subject)/mri/aparc+aseg.mgz: | memprage/T1.nii.gz
           source /usr/local/freesurfer/stable5_3/SetUpFreeSurfer.sh && \
           export SUBJECTS_DIR=$(SUBJECTS_DIR) && \
           /usr/local/freesurfer/stable5_3/bin/recon-all -i $< -subjid $(subject) \
           -all && \
           touch $(SUBJECTS_DIR)/$(subject)

25 xfm_dir/fs_to_T1.mat: $(SUBJECTS_DIR)/$(subject)/mri/aparc+aseg.mgz
    memprage/T1.nii.gz
           mkdir -p xfm_dir && \
           source /usr/local/freesurfer/stable5_3/SetUpFreeSurfer.sh && \
           export SUBJECTS_DIR=$(SUBJECTS_DIR) && \
```

```
        tkregister2 --mov $(SUBJECTS_DIR)/$(subject)/mri/orig.mgz \
        --targ $(word 2,$^) --noedit --regheader \
        --reg xfm_dir/fs_to_T1.dat \
        --fslregout xfm_dir/fs_to_T1_init.mat && \
        mri_convert $(SUBJECTS_DIR)/$(subject)/mri/orig.mgz \
        $(SUBJECTS_DIR)/$(subject)/mri/orig.nii.gz && \
        $(FSL_DIR)/bin/flirt -ref $(word 2,$^) \
        -in $(SUBJECTS_DIR)/$(subject)/mri/orig.nii.gz \
        -init xfm_dir/fs_to_T1_init.mat -omat $@

26 xfm_dir/fs_to_Resting.mat: xfm_dir/fs_to_T1.mat xfm_dir/T1_to_Resting.mat
        $(FSL_DIR)/bin/convert_xfm -concat $(word 2, $^) -omat $@ $<
```

This code creates registrations between functional, structural, and standard space as well as brain segmentation using FreeSurfer.

22 Registration is created from functional to structural space.

23 Although the recipe above creates both `.mat` and `.nii.gz` files created by `epi_reg`, later recipes depending on `Resting_to_T1.nii.gz` will fail unless we add this empty recipe referring back to the rule for the `.mat` file. Although it is possible to simply make these later recipes depend on the `.mat` file despite using the `.nii.gz` file, the workflow is both more readable and easier to debug if we indicate the relationship between the two files here and reference either file as called for later.

24 Here FreeSurfer is called to segment the brain, with the recipe targeting a label file which is generated at the end of `recon-all`. The '`|`' (pipe) symbol here indicates that `T1.nii.gz` is an 'order only' dependency, which means that `recon-all` will not be re-run if `T1.nii.gz` is newer than `aparc+aseg.mgz`. The purpose here being that FreeSurfer is often run separately from individual analyses, and it may be undesirable for the pipeline to re-run `recon-all` each time the code generating `T1.nii.gz` is changed. Other applications may be better served with a normal dependency, in which case the pipe can be omitted.

25 Here the FreeSurfer image is registered on to the T1 structural image. `tkregister2` is used to generate an initial affine registration matrix. The final registration is generated by `flirt`, using `tkregister2`'s matrix as a starting point so as to avoid local minima.

26 Registration from FreeSurfer to functional space is generated by concatenating `fs_to_T1.mat` with `T1_to_Resting.mat` using `convert_xfm`.

Generation of Nuisance Regressors - `tapping/lib/resting/Regressors.mk`

```
.PHONY: regressors
regressors: resting/wm.txt resting/csf.txt resting/nuisance_regressors.txt

27 resting/dvars_regressors: resting/Resting_brain.nii.gz
        $(SCRIPTpath)/motion_outliers -i $^ -o $@ --dvars -s resting/dvars_vals\
         --nomoco && \
        rm resting/dvars_vals && \
        touch $@

28 resting/fd_regressors: resting/Resting_brain.nii.gz resting/Resting.par
        $(SCRIPTpath)/motion_outliers -i $^ -o $@ --fd -s resting/fd_vals \
        -c resting/Resting.par --nomoco --thresh=3 && \
        rm resting/fd_vals && \
        touch $@

29 resting/all_outliers.txt: resting/dvars_regressors resting/fd_regressors
        cat resting/dvars_spike_vols | $(SCRIPTpath)/transpose \
        > resting/alloutliers_nsort.txt && \
        cat resting/fd_spike_vols | $(SCRIPTpath)/transpose \
```

```
        >> resting/alloutliers_nsort.txt && \
        sort -nu resting/alloutliers_nsort.txt > $@ && \
        rm resting/alloutliers_nsort.txt

30 resting/outlier_regressors.txt: resting/Resting_orient.nii.gz
resting/all_outliers.txt
        vols=‘fslval $< dim4’ && \
        echo "vols is $$vols" && \
        python ${SCRIPTpath}/SinglePointGenerator.py -i $(word 2,$^) -v $$vols \
        -o $@ -p resting/resting_percent_outliers.txt
```

This code generates motion outlier regressors in feat compatible syntax.

27 - 28 FSL's `motion_outliers` script is called to generate lists of motion outliers using two separate metrics (RMS intensity difference of volume N to volume N+1 and frame displacement, respectively - see (Power et al., 2012)). A separate regressor is generated for each outlier.

29 The resulting two regressor files are transposed (rotated) and sorted numerically, effectively sorting the outlier volumes by time (duplicates from the two sources will appear next to each other).

30 The python script `SinglePointGenerator.py` then extracts unique outliers and returns them in standard column format.

```
31 fs_wm_mask.nii.gz: $(SUBJECTS_DIR)/$(subject)/mri/aparc+aseg.mgz
        source /usr/local/freesurfer/stable5_3/SetUpFreeSurfer.sh && \
        export SUBJECTS_DIR=$(SUBJECTS_DIR) && \
        mri_binarize --i $< --o $@ --erode 1 --wm

32 resting/wm.txt: fs_wm_mask.nii.gz resting/Resting_mc.nii.gz
xfm_dir/T1_to_Resting.mat
        $(FSL_DIR)/bin/flirt -ref $(word 2,$^) -in $< -out wm.nii.gz  -applyxfm \
        -init $(word 3,$^) && \
        $(FSL_DIR)/bin/fslmaths wm.nii.gz -thr .5 wm.nii.gz && \
        $(FSL_DIR)/bin/fslmeants -i $(word 2,$^) -o  $@ -m wm.nii.gz

33 fs_ventricles_mask.nii.gz: $(SUBJECTS_DIR)/$(subject)/mri/aparc+aseg.mgz
        source /usr/local/freesurfer/stable5_3/SetUpFreeSurfer.sh && \
        export SUBJECTS_DIR=$(SUBJECTS_DIR) && \
        mri_binarize --i $< --o $@ --erode 1 --ventricles

34 resting/csf.txt: fs_ventricles_mask.nii.gz resting/Resting_mc.nii.gz
xfm_dir/T1_to_Resting.mat
        $(FSL_DIR)/bin/flirt  -ref $(word 2,$^) -in $(word 1,$^) \
        -out `dirname $@`/`basename $@ .txt`.nii.gz  \
        -applyxfm -init $(word 3,$^) && \
        $(FSL_DIR)/bin/fslmaths `dirname $@`/`basename $@ .txt`.nii.gz \
        -thr .5 `dirname $@`/`basename $@ .txt`.nii.gz && \
        $(FSL_DIR)/bin/fslmeants -i $(word 2,$^) -o  $@ \
        -m `dirname $@`/`basename $@ .txt`.nii.gz

35 resting/nuisance_regressors.txt: resting/csf.txt resting/wm.txt
resting/Resting.par resting/outlier_regressors.txt resting/Resting_brain.nii.gz
        paste $(word 1,$^) $(word 2,$^) $(word 3,$^) $(word 4,$^) > $@
```

Here we generate noise correction regressors from signal in white matter and CSF regions.

31 A binarized mask of FreeSurfer's white matter volume is generated in the first recipe.

32 The mask from 31 is then put in to functional space using `flirt` and binarized again to remove any gray areas generated during registration. Finally, `fslmeants` is used to generate a timeseries containing outliers in WM activity.

33 -34 The same procedure used above is repeated with FreeSurfer's CSF volume.

35 To generate the final nuisance regressors file to be input to `feat`, `paste` is used to combine the three outlier regressor files with the rigid body motion regressors generated in preprocessing.

ROI Timeseries Extraction - `lib/resting/timeseries.mk`

```
.PHONY: timeseries

timeseries: resting/timeseries/ctx-rh-superiorfrontal.txt
         resting/timeseries/r-sfg-mask.nii.gz

resting/timeseries/ctx-rh-superiorfrontal.txt: resting/Resting_ssmooth.nii.gz
resting/timeseries/r-sfg-mask.nii.gz
36        fslmeants -i $< -m $(word 2, $^) -o $@

37 resting/timeseries/r-sfg-mask.nii.gz: resting/timeseries/ctx-rh-superiorfrontal.
   nii resting/Resting_ssmooth.nii.gz xfm_dir/fs_to_Resting.mat
        flirt -in $< -applyxfm -init $(word 3, $^) \
        -out resting/timeseries/rsfg-funcspace.nii.gz -paddingsize 0.0 \
        -interp trilinear -ref $(word 2, $^) && \
        fslmaths resting/timeseries/rsfg-funcspace.nii.gz -bin $@ && \
        rm resting/timeseries/rsfg-funcspace.nii.gz

38 $(SUBJECTS_DIR)/$(subject)/register.dat: $(SUBJECTS_DIR)/$(subject)/mri/aparc+aseg
   .mgz
        tkregister2 --mov $(SUBJECTS_DIR)/$(subject)/mri/T1.mgz --noedit \
                --s $(subject) --regheader --reg $@

39 $(SUBJECTS_DIR)/$(subject)/labels2/aparc+aseg-in-rawavg.mgz: $(SUBJECTS_DIR)/$(
   subject)/register.dat
        mkdir -p $(SUBJECTS_DIR)/$(subject)/labels2/ ;\
        mri_label2vol --seg $(SUBJECTS_DIR)/$(subject)/mri/aparc+aseg.mgz \
        --reg $(SUBJECTS_DIR)/$(subject)/register.dat --o $@ \
        --temp $(SUBJECTS_DIR)/$(subject)/mri/aparc+aseg.mgz

40 resting/timeseries/ctx-rh-superiorfrontal.nii: $(SUBJECTS_DIR)/$(subject)/labels2/
   aparc+aseg-in-rawavg.mgz
        mkdir -p resting/timeseries && \
        mri_binarize --i $(SUBJECTS_DIR)/$(subject)/labels2/aparc+aseg-in-rawavg.
           mgz \
        --match 2028 --o resting/timeseries/ctx-rh-superiorfrontal.nii
```

Here a mask of the ROI (Right SFG) is generated from FreeSurfer and registered to functional space; a timeseries of activity in the ROI is then generated using FSL. The recipes are written roughly in the reverse order they are run, starting with the desired endpoint and working backwards through all required dependencies. `make` will execute them in the desired order according to the dependency structure.

36 The `fslmeants` command generates a timeseries of average activation from the preprocessed resting image within the area defined by the specified mask file.

37 The registered mask file is generated by applying the registration matrix transforming FreeSurfer to functional space generated in 26 to the mask of the ROI generated with FreeSurfer. The mask must be binarized using `fslmaths`, as registration will generate non-binary values around the edges of the mask. The non-binarized mask is then removed.

38 An image indexed by FreeSurfer's aseg and aparc atlases is generated using `mri_label2vol`.

39 The (unregistered) FreeSurfer mask for the ROI is generated from the labeled volume by using `mri_-binarize` to isolate image voxels whose value matches the relevant FreeSurfer label (the index value for the `ctx-rh-superiorfrontal` area is 2028 - see `FreeSurferColorLUT.txt` in your FreeSurfer install directory for a list of volume labels).

First Level Analysis - `lib/resting/fsl.mk`

```
.PHONY: feat
.SECONDARY:

41 feat: resting/Resting.feat/stats/cope1.nii.gz

42 resting/Resting.feat/stats/cope1.nii.gz: resting/Resting.fsf resting/
   Resting_ssmooth.nii.gz
resting/nuisance_regressors.txt $(FSL_DIR)/data/standard/MNI152_T1_2mm_brain.nii.
   gz
resting/timeseries/ctx-rh-superiorfrontal.txt
        rm -rf resting/Resting.feat && \
        $(FSL_DIR)/bin/feat resting/Resting.fsf

43 resting/Resting.fsf: $(projdir)/lib/resting/Tapping_FirstLevel.fsf
resting/Resting_ssmooth.nii.gz
        sed -e 's/SUBJECT/$(subject)/g' $< > $@
```

Here a template `.fsf` file is copied from the `lib` directory and `feat` is run. Because there is only one functional run per subject, only the subject number need be substituted when copying the template. 41 `feat` Creates many files, but as the first cope is the one we are most interested in (and will only generate if the program runs successfully), it is listed as the recipe target. Therefor the phony target amounts to an alias, as there is only one dependency.

42 First, any previous runs of `fsl` are removed - otherwise `fsl` will continually create new directories when `feat` is rerun, leading to inconsistent file structures between subjects and breaking the `make` dependency structure. To execute the analysis, `feat` is simply called on the fsf file.

43 The fsf file is generated per subject in this recipe by using `sed` to copy a template while substituting the subject number for the text `SUBJECT` in the original. If further by-subject customization is needed, the `-e` flag can be provided to perform additional substitutions.

Quality Assurance Reports - `lib/resting/qa.mk`

```
.PHONY=qa-all prept1-qa prepfunc-qa timeseries-qa

qa-all: prept1-qa prepfunc-qa timeseries-qa QA-report.html

QA-report.html: ../lib/resting/subject_QA_report.html
44      sed -e 's/SUBJECTNUMBER/$(subject)/g' $< > $@

prept1-qa: memprage/QA/images/T1_brain.png

45 memprage/QA/images/rendered_T1_brain.nii.gz: memprage/T1.nii.gz
memprage/T1_brain_mask.nii.gz
        mkdir -p `dirname $@` && \
        $(FSL_DIR)/bin/overlay 1 1 $(word 1,$^) -a $(word 2,$^) 1 10 $@

46 memprage/QA/images/T1_brain.png: memprage/QA/images/rendered_T1_brain.nii.gz
        $(SCRIPTpath)/slices memprage/QA/images/rendered_T1_brain.nii.gz -o $@
```

```
prepfunc-qa: resting/QA/images/Resting_ssmooth_mid_animation.gif resting/QA/images
   /resting_to_T1.png

47 resting/QA/images/Resting_ssmooth_mid_animation.gif:
resting/Resting_ssmooth.nii.gz
        mkdir -p `dirname $@` && \
        $(SCRIPTpath)/functional_movies.sh $< `dirname $@`

48 resting/QA/images/resting_to_T1.png: xfm_dir/Resting_to_T1.nii.gz
memprage/T1_brain.nii.gz
        mkdir -p `dirname $@` && \
        $(SCRIPTpath)/sliceappend.sh -1 $(word 1,$^) -2 $(word 1,$^) -o $@ -s

timeseries-qa: resting/QA/images/rendered_r-sfg-mask.png

49 resting/QA/images/rendered_r-sfg-mask.nii.gz: resting/Resting_brain.nii.gz
resting/timeseries/r-sfg-mask.nii.gz
        mkdir -p `dirname $@` && \
        $(FSL_DIR)/bin/overlay 1 1 $(word 1,$^) -a $(word 2,$^) 1 10 $@

50 resting/QA/images/rendered_r-sfg-mask.png:
resting/QA/images/rendered_r-sfg-mask.nii.gz
        $(SCRIPTpath)/slices $< -o $@
```

A simple quality assurance (QA) report is generated per subject from an `.html` template.

44 The template is copied from the lib directory using `sed` to substitute the appropriate subject number. Because filenames are preserved within subjects and relative paths are used in the html code to indicate paths to qa images, no other substitutions are necessary.

45 The FSL utility `overlay` is used to generate an image of the skull-stripped structural image in red overlaying the original T1, allowing for visual inspection of the quality of the skull strip.

46 The `slices` script generates an image of several slices of the above overlay volume for easy visual inspection. As elsewhere, these steps could be written in a single recipe, but separating it to smaller recipes allows for cleaner code and easier debugging.

47 The `functional_movies.sh` script creates an animated `.gif` file displaying slices of the functional volume animated over time. This allows for visual inspection of gross movement and other imaging artifacts.

48 The `sliceappend.sh` script allows for the inspection of registration by presenting an outline of one image on top of a second image to which it has been registered on one row of slices and the reverse on the next.

49-50 The same method as was used for the inspection of skull stripping is used to inspect the quality of the ROI extraction.

Example 10

Using ANTs Registration with FEAT

Kelly A. Sambrook[1]

This is an example of using Advanced Normalization Tools (ANTs)(Avants et al., 2014) registration with FSL Feat. ANTs provides a superior nonlinear registration from the T1 to MNI brain compared to FSL's `fnirt` command, so we use it here in conjunction with FSL's `epi-reg` to replace the functional to standard space registrations created by FEAT with those created by ANTs.

The code for this example is in `$MAKEPIPELINES/ANTsreg/` in specific makefiles described below. It is assumed that the fMRI data here have been pre-processed (including motion correction) before running this pipeline.

Note that to run this example, and indeed, to use ANTs with FSL, you must have ANTs and ITK-SNAP Convert3D tools installed. We assume that Convert3D tools (specifically, `c3d_affine_tool`) are installed in your bin directory and is available in your path. You can type:

```
$ which c3d_affine_tool
```

to determine where it is located if it is in your path. ANTs, however, is another story; you may have a different version than we do and it may be installed in a different location. So please note that you will probably need to edit this example to specify your location for ANTs.

Group Level Makefile

```
SUBJECTS = TS001 TS002
.PHONY:  all $(SUBJECTS)

all:  $(SUBJECTS)

$(SUBJECTS):
        $(MAKE) --directory=$@ $(TARGET)
```

The code for this makefile can be found at `$MAKEPIPELINES/ANTsreg/Makefile`. There are two subjects in this directory that require the same subject-level processing. Of course, in a real study there would be many more. This group level makefile is a driver that will recursively call targets defined the subject-level makefiles. See Setting up an Analysis Directory for more information on this type of directory structure.

[1] kelly89@uw.edu

Subject Level Makefile

The subject-level makefile is in `ANTsreg/Makefile.subject`. Each subject directory has a symbolic link to this file that is named `Makefile`. In this way, we can change it in the top-level directory but all subjects will immediately see the changes.

```
cwd = $(shell pwd)
subject=$(notdir $(cwd))
```

We use the directory name here to set a variable that contains the name of the subject. This is handy when full pathnames must be specified to programs.

```
❶ export OMP_NUM_THREADS=1

SHELL=/bin/bash
ifeq "$(origin MAKEPIPELINES)" "undefined"
MAKEPIPELINES=/project_space/makepipelines
endif

PROJECT_DIR=$(MAKEPIPELINES)/ANTsreg
STANDARD_DIR=$(PROJECT_DIR)/Standard
SUBJECT_DIR=$(PROJECT_DIR)/$(subject)

❷ ANTSpath=/usr/local/ANTs-2.1.0-rc3/bin/

❸ BLOCKS= TappingBlock1 TappingBlock2

❹ FSLDIR=/usr/share/fsl/5.0/

STD_BRAIN=$(FSLDIR)/data/standard/MNI152_T1_2mm_brain.nii.gz
STD=$(FSLDIR)/data/standard/MNI152_T1_2mm.nii.gz
```

Here we define a number of variables that are used by other makefiles.
❶ ANTs exploits multiple cores to speed up execution. However, if we are running this pipeline across many subjects using `make`, we disable this parallelism by setting the variable `OMP_NUM_THREADS` to be one.
❷ This is where ANTs is located on our systems. It is unlikely that ANTs is in the same directory on your system, so you will have to edit this line.
❸ Similarly, the location of FSL may be different on your system. Check the location of `FSLDIR` on your system and uncomment it as shown in this example. Note that if FSL is in a different location on your system, you will have to edit the Feat template files, located in `ANTsreg/templates`.
❹ We set a variable called `BLOCKS` to the names of the fMRI tapping task runs. This saves some typing later.

```
antsreg: PrepSubject Feat

include ../lib/makefiles/Prep.mk
include ../lib/makefiles/Feat.mk

clean:   clean_prep clean_feat
```

The main target here, `antsreg`, creates some basic registrations (defined by the `PrepSubject` target in the `Prep.mk` makefile) and then runs FEAT, creating ANTs registrations in the FEAT directories (target `Feat` defined in the `Feat.mk` makefile). These makefiles are stored in the `lib` directory and are included by the subject-level makefile.

Finally, we define a `clean` target that depends upon the `clean_prep` and `clean_feat` targets, defined in their respective makefiles.

Preparatory Registrations - `Prep.mk`

For convenience we have separated the main work of performing registrations with ANTs from the task of running Feat and applying the ANTs registrations to the Feat output. The registrations are defined in the Makefile `ANTsreg/lib/makefiles/Prep.mk`, described in this section.

```
.PHONY: PrepSubject bet struct_registrations epi_registrations clean_prep
PrepSubject: bet struct_registrations epi_registrations

5 Drop1Sfx = $(basename $(1))
```

We define phony targets as usual, and the main target is the `PrepSubject` target. 5 We also define a helpful little function, `Drop1Sfx` that drops the suffix of its argument. This is accomplished by using the `make` defined function `basename`.

```
6 bet: MPRAGE/T1_brain.nii.gz $(patsubst %,Tapping/%_brain.nii.gz, $(BLOCKS))

MPRAGE/T1_brain.nii.gz: MPRAGE/T1.nii.gz
        bet $^ $@ -R

7 Tapping/%_brain.nii.gz: Tapping/%.nii.gz
        bet $< $@ -F
```

We define a phony target, `bet`, to perform skull stripping of the MPRAGE and functional images. 6 We use pattern substitution on the `BLOCKS` variable to form the names of the skull-stripped functional images. 7 An implicit rule is used to create the skull-stripped functional images. This simplifies the Makefile when there are multiple functional runs that all need to be processed in the same way.

```
struct_registrations: xfm_dir/T1_to_mni_Warp.nii.gz

xfm_dir/T1_to_mni_Warp.nii.gz: MPRAGE/T1_brain.nii.gz
        mkdir -p xfm_dir ;\
        export ANTSPATH=$(ANTSpath) ;\
        $(ANTSpath)/antsIntroduction.sh -d 3 -i MPRAGE/T1_brain.nii.gz \
        -m 30x90x20 -o $(SUBJECT_DIR)/xfm_dir/T1_to_mni_ \
        -s CC -r $(STD_BRAIN) -t GR
```

The structural registration of the MPRAGE to the standard brain is performed using ANTs, using the script `antsIntroduction.sh`. The target file `T1_to_mni_Warp.nii.gz` will be created by this script because we have specified the output prefix using the `-o` flag.

```
epi_registrations: $(patsubst %,xfm_dir/%_to_T1.mat,$(BLOCKS))
$(patsubst %,xfm_dir/%_to_mni_epireg_ants.nii.gz,$(BLOCKS))

8 Tapping/%_brain_vol0.nii.gz: Tapping/%_brain.nii.gz
        fslroi $< $@ 0 1

xfm_dir/%_to_T1.mat: Tapping/%_brain_vol0.nii.gz MPRAGE/T1.nii.gz
MPRAGE/T1_brain.nii.gz
        mkdir -p xfm_dir ;\
    9   epi_reg --epi=Tapping/$*_brain_vol0  \
        --t1=MPRAGE/T1.nii.gz --t1brain=MPRAGE/T1_brain.nii.gz \
        --out=$(call Drop1Sfx,$@)
```

We use the target `epi_registrations` to perform the registrations involving the functional data. Our first step is to use `epi_reg` from FSL to perform boundary-based registration of the functional image to the MPRAGE. 8 We take the first volume of each skull-stripped functional run to use as the input volume for `epi_reg`. This neat trick (thanks Katie Askren!) allows `epi_reg` to run faster and use a lot less memory. 9 Note that we get to use the `Drop1Sfx` function that we defined earlier on the target to specify the output path to `epi_reg`.

```
10 xfm_dir/%_to_T1_ras.txt: MPRAGE/T1_brain.nii.gz Tapping/%_brain_vol0.nii.gz
   xfm_dir/%_to_T1.mat
        c3d_affine_tool -ref MPRAGE/T1_brain.nii.gz \
        -src Tapping/$*_brain_vol0 xfm_dir/$*_to_T1.mat -fsl2ras -oitk $@

11 xfm_dir/%_to_mni_epireg_ants.nii.gz: Tapping/%_brain_vol0.nii.gz $(STD_BRAIN)
   MPRAGE/T1_brain.nii.gz xfm_dir/%_to_T1_ras.txt xfm_dir/T1_to_mni_Warp.nii.gz
        export ANTSPATH=$(ANTSpath) ;\
        $(ANTSpath)/WarpImageMultiTransform 3 Tapping/$*_brain_vol0.nii.gz $@ \
        -R $(STD_BRAIN) xfm_dir/T1_to_mni_Warp.nii.gz \
        xfm_dir/T1_to_mni_Affine.txt xfm_dir/$*_to_T1_ras.txt
```

Now comes the moment where we combine the T1 to standard space registration that we made with ANTs with the functional to T1 registration that we did with `epi_reg`. To do this you need to use `WarpImageMultiTransform` from the ANTs package. **10** We first convert the FSL style transformation matrices to ITK format using `c3d_affine_tool`. **11** Then we use `WarpImageMultiTransform` to combine this matrix with the T1 to standard space warp and affine matrix. Voila! We have achieved registration.

```
clean_prep:
        rm -rf  */*_brain*.nii.gz */*vol0.nii.gz xfm_dir
```

Finally, we define a target `clean_prep` to remove all files created by this makefile.

Running Feat and Applying ANTs Registrations - `Feat.mk`

The next step is to run first level Feats, apply the ANTs registrations to the resulting output files, and then run a second level Feat for each individual. This is done by the Makefile described here, located in `ANTsreg/lib/makefiles/Feat.mk`.

```
TEMPLATES=$(PROJECT_DIR)/templates

Tapping_FEAT1_TEMPLATE=$(PROJECT_DIR)/templates/Tapping_FL.fsf
Tapping_FEAT2_TEMPLATE=$(PROJECT_DIR)/templates/Tapping_HL.fsf
```

We run Feat on multiple blocks with multiple subjects by defining Feat templates. We create these by setting up Feat runs for a single subject using the graphical user interface and saving the configuration file. Then we edit that file, replacing things like subject ID and the run name with capitalized text strings that we can find and replace in a makefile with their real values. Here we define variables for these templates (first level and higher level).

```
.PHONY: Feat FirstLevelFeats FirstLevelReg SecondLevelFeats
.SECONDARY: $(allupdatereg)

12 allcopes=$(wildcard Tapping/*.feat/stats/cope*.nii.gz)
allvarcopes=$(wildcard Tapping/*.feat/stats/varcope*.nii.gz)
13 allupdatereg=$(subst stats,reg_standard/stats,$(allcopes))
$(subst stats,reg_standard/stats,$(allvarcopes))
allupdatetdof= $(subst stats/cope,reg_standard/stats/FEtdof_t,$(allcopes))

Feat: FirstLevelFeats FirstLevelReg SecondLevelFeats
```

We define our phony targets as usual.

Our goal is to transform the copes and varcopes into standard space using the ANTs registration. **12** However, because there may be an arbitrary number of copes, we use a wildcard here to obtain their names. **13** We use pattern substitution on the names that we obtained from wildcards to define the filenames for the updated registrations that we need to create. Similarly, we also define the DOF (degrees of freedom) files that would normally be created by Feat.

```
FirstLevelFeats: $(patsubst %,Tapping/%.feat/stats/cope4.nii.gz, $(BLOCKS))

Tapping/%.feat/stats/cope4.nii.gz: $(patsubst %,Tapping/%_brain.nii.gz,
   TappingBlock1 TappingBlock2)
        rm -rf ‘echo $@ | awk -F "/" ‘{print $$1"/"$$2}’’ ;\
        NBVOLS=‘fslval $(word 1,$^) dim4’ ;\
        RUN=$* ;\
        RUN_NUM=‘echo $${RUN} | sed -e ‘s/TappingBlock//g’’ ;\
        sed -e ‘s|SUBDIR|$(SUBJECT_DIR)|g’ -e "s/NB/$${NBVOLS}/g" \
        -e "s/RUN/$${RUN_NUM}/g" -e "s/SUBJECT/$(subject)/g" \
        $(Tapping_FEAT1_TEMPLATE) > Tapping/$*.fsf ;\
        feat Tapping/$*.fsf ;\
```

This block of code runs Feat for the two fMRI blocks. We use the last cope (cope 4) as the target file to indicate that the recipe has completed correctly. We set variables for the number of volumes, the run name, and the run number, and use these (in addition to the subject and subject directory) to modify the Feat template before running Feat.

```
14 FirstLevelReg:  FirstLevelFeats $(allupdatereg)
$(patsubst %,Tapping/%.feat/reg_standard/mask.nii.gz, $(BLOCKS))
$(patsubst %,Tapping/%.feat/reg_standard/mean_func.nii.gz, $(BLOCKS))
$(patsubst %,Tapping/%.feat/reg_standard/example_func.nii.gz, $(BLOCKS))
$(allupdatetdof) $(patsubst %,Tapping/%.feat/reg/standard.nii.gz,
$(BLOCKS)) $(patsubst %,Tapping/%.feat/reg/example_func2standard.mat, $(BLOCKS))

15 define make-cope
Tapping/%.feat/reg_standard/stats/$(notdir $(1)): Tapping/%.feat/stats/$(notdir $
   (1)) xfm_dir/T1_to_mni_Warp.nii.gz
xfm_dir/T1_to_mni_Affine.txt xfm_dir/%_to_T1_ras.txt
        mkdir -p `dirname $$@` ;\
        export ANTSPATH=$(ANTSpath) ;\
        $(ANTSpath)/WarpImageMultiTransform 3 $$(word 1,$$^) $$@ -R $(STD_BRAIN) \
        $$(word 2,$$^) $$(word 3,$$^) $$(word 4,$$^)
endef

16 $(foreach c,$(allupdatereg),$(eval $(call make-cope,$c)))
```

After running Feat, we need to create all the standard space files that Feat normally would create, using ANTs registration instead of FEAT to do so. **14** This target defines all the files that need to be created for Feat using ANTs registration matrices. We make liberal use of pattern substitution here to form these names. **15** Because we have multiple fMRI runs (TappingBlock1 and TappingBlock2) and a lot of copes that we wish to register to standard space, we cannot simply use implicit rules to describe how to transform them. It would be nice if you could use one character to substitute for the tapping block name, and another character for the cope number, but `make` doesn't work that way. So you could use a pattern to substitute for the tapping block name and explicitly write out rules for each of the files to register.

Instead, we use a combination of canned recipes, the `call` function, and the `eval` function to create the registration rules for the copes and varcopes "on the fly" in `make`. The first step here is to define the canned recipe `make-cope`, which uses the filename of its first argument to create the snippet of recipe that registers the cope or varcope file in the stats directory to standard space. Note that there are two dollar signs for every one in the recipe. This is necessary because of how this recipe will be evaluated. **16** This line does the magic of creating new makefile rules out of our `make-cope` variable. The `foreach` function goes through the list of files in the `allupdatereg` variable that we defined earlier, calling each of them `c`. For each file, it then evalutes (using `eval`) the output of calling `make-cope` on each of the files. The `call` function creates a `make` recipe for each file, and then `eval` causes `make` to actually evaluate these new rules, as if they had been written out in

the Makefile from the beginning. The argument to `eval` is expanded twice: first by `eval` and then by `make`, which is why there are extra `$` characters.

This is long and a little tricky but it does the job.

```
Tapping/%.feat/reg_standard/example_func.nii.gz: Tapping/%.feat/example_func.nii.
   gz xfm_dir/T1_to_mni_Warp.nii.gz xfm_dir/T1_to_mni_Affine.txt xfm_dir/%
   _to_T1_ras.txt
        mkdir -p `dirname $@`;
        $(ANTSpath)/WarpImageMultiTransform 3 Tapping/$*.feat/example_func.nii.gz
           $@ -R $(STD_BRAIN) xfm_dir/T1_to_mni_Warp.nii.gz xfm_dir/
           T1_to_mni_Affine.txt xfm_dir/$*_to_T1_ras.txt

Tapping/%.feat/reg_standard/mean_func.nii.gz: Tapping/%.feat/mean_func.nii.gz
   xfm_dir/T1_to_mni_Warp.nii.gz xfm_dir/T1_to_mni_Affine.txt xfm_dir/%_to_T1_ras.
   txt
        mkdir -p `dirname $@`;
        $(ANTSpath)/WarpImageMultiTransform 3 Tapping/$*.feat/mean_func.nii.gz \
        $@ -R $(STD_BRAIN) xfm_dir/T1_to_mni_Warp.nii.gz \
        xfm_dir/T1_to_mni_Affine.txt xfm_dir/$*_to_T1_ras.txt

Tapping/%.feat/reg_standard/mask.nii.gz: Tapping/%.feat/mask.nii.gz xfm_dir/
   T1_to_mni_Warp.nii.gz xfm_dir/T1_to_mni_Affine.txt xfm_dir/%_to_T1_ras.txt
        mkdir -p `dirname $@`;
        $(ANTSpath)/WarpImageMultiTransform 3 Tapping/$*.feat/mask.nii.gz \
        $@ -R $(STD_BRAIN) xfm_dir/T1_to_mni_Warp.nii.gz \
        xfm_dir/T1_to_mni_Affine.txt xfm_dir/$*_to_T1_ras.txt

Tapping/%.feat/reg/standard.nii.gz: xfm_dir/T1_to_mni_Warp.nii.gz
        mkdir -p `dirname $@`;
        cp $< $@

Tapping/%.feat/reg/example_func2standard.mat: $(TEMPLATES)/selfreg.mat
        mkdir -p `dirname $@`;
        cp $< $@
```

There are a variety of files that need to be registered using ANTs that can easily be specified using implicit rules parameterized by the block name. We do not need to use `eval` to create these rules; we can just write them.

```
define make-tdof
Tapping/%.feat/reg_standard/stats/$(notdir $(1)):
Tapping/%.feat/stats/cope1.nii.gz Tapping/%.feat/stats/dof
        mkdir -p Tapping/$$*.feat/reg_standard/stats ;\
        fslmaths Tapping/$$*.feat/stats/cope1.nii.gz -mul 0 \
        -add `cat Tapping/$$*.feat/stats/dof` $$@
endef

$(foreach c,$(allupdatetdof),$(eval $(call make-tdof,$c)))
```

Just like the copes, the degrees of freedom files (dof) can be created by defining a canned parameterized recipe to create them. These files are just NIfTI files of the same dimensions as the cope with the degrees of freedom of the analysis in all voxels. The parameterized recipe takes the dof filename as an argument, uses the `call` function create a `make` recipe for each dof file, and then use `eval` to have `make` evaluate these new rules!

```
SecondLevelFeats: Tapping/Tapping_FixedFX.gfeat/cope4.feat/stats/zstat4.nii.gz

Tapping/Tapping_FixedFX.gfeat/cope4.feat/stats/zstat4.nii.gz:
```

```
$(Tapping_FEAT2_TEMPLATE) $(patsubst % ,Tapping/%.feat/mask.nii.gz,$(BLOCKS))
   FirstLevelReg
        rm -rf ‘echo $@ | awk -F "/" ’{print $$1"/"$$2}’‘ ;\
        featName=‘echo $@ | awk -F "/" ’{print $$2}’ | \
        awk -F ".gfeat" ’{print $$1}’‘ ;\
        sed -e ’s|SUBDIR|$(SUBJECT_DIR)|g’ $(word 1,$^) \
        > Tapping/$${featName}.fsf ;\
        feat Tapping/$${featName}.fsf
```

After all the ANTs registrations have been applied we can run the second level Feats. We use the last zstat file as a marker that indicates Feat has completed successfully. As in the first level Feat, we modify a template to specify the subject and the block.

```
clean_feat:
        rm -rf Tapping/*.*feat Tapping/*.fsf
```

Finally, we define a target to clean up all files that we have created!

The next step in processing, not covered in this example, would be to perform group level analysis across all subjects.

Example 11

Creating Result Tables Automatically Using Make

Maya Reiter[1]

Creating result tables illustrating the results of fMRI group analysis takes a LOT of time and effort, especially because FSL does not provide anatomical labels for each significant cluster. It is possible to identify anatomical regions of significant clusters using FSL `atlasquery` by hand, for each cluster, and copy-paste these labels into a spreadsheet. This takes (or, I speculate that it takes — as I decided that there must be a better way before even trying) hours on end! Moreover, if anything changes in your model, you will have to do this all over again! Rest assured, there is a better way.

In this example, I will introduce a program that I wrote (in `bash` and Python), that creates result tables out of group analysis directories (truth be told, it also works on individual first level .feat directories). The product of this program is an almost-ready version of a table that you would publish in a journal article (Yay!). I have also found the result tables generated by this program a useful tool to display results as I am interpreting them. It's a nice way to "see it all at once".

This is also an example of how `make` can help you parallelize your favorite scripts, if you do not wish to re-write them in `make`. In this case, I had spent a full week creating this software using `bash` and Python, and was pleased with the way it was working. Nevertheless, using `make`, I was able to parallelize this code using the `-j` flag. This allowed me to create result tables for different group analyses in parallel, and saved me a lot of computer time (my program takes a long time to run if there are many significant clusters).

To run this example, you will need the example group analysis directory (`GROUP_2BACK.gfeat`), the Makefile, and all of the scripts in the `bin` directory. You will also need to have Python and FSL installed. Group Feat (`.gfeat`) directory names may **not** exceed 20 characters because spreadsheet tab names cannot exceed 20 characters.

The code for this example is in `$MAKEPIPELINES/Gen_Result_Tables/Makefile`.

Simple Result Tables

```
SHELL=/bin/bash

all: results1.txt results2.xlsx

1 results1.txt: GROUP_2BACK.gfeat
	bin/mScript_Get_feat_stats $< ;\
```

[1] mayar15@uw.edu

```
        mv GROUP_2BACK.gfeat_Feat_Results $@

❷ results2.xlsx: results1.txt
        bin/mScript_make_result_tables -o $@ -f $(word 1,$^)
```

There are only two targets in this makefile: `results1.txt` and `results2`. ❶ `results1.txt` is a preliminary result table that has separate cells for the anatomical label as defined by each of the three atlases I chose to implement ("Harvard-Oxford Cortical Structural Atlas", "Harvard-Oxford Subcortical Structural Atlas", "Cerebellar Atlas in MNI152 space after normalization with FLIRT"). It also includes all atlas-defined regions encompassed by the clusters, no matter how low the probability that they belong to the region according to the atlas (0.07% probability frontal pole gets included in this table). Indeed this table is useful for checking the probability that each cluster or peak of the cluster belongs to the anatomical region/structure defined by the atlas.

Other than that, this table is a pain to look at. Each atlas is incomplete in terms of providing anatomical labels for the whole brain. Thus, looking up a cerebellum cluster in the Harvard-Oxford Subcortical Structural Atlas will leave you with a blank cell in this table. (As a side note, this program does not implement all atlases, and as such, does not provide specific anatomical labels for the brainstem or various nuclei, etc. This functionality can be added but I haven't done it yet).

❷ `results2.xlsx` is a far cleaner result table than `results1.txt`. The peak region is looked up in the three atlases, and the other regions that the cluster encompases are listed by order of probability (NOT peak/z-score). Only labels that have a probability of more than 5% of belonging to that cluster are listed. Labels in this result table do not include white matter. Also, the program figures out which of the three atlases to use.

Multiple Group Analyses

The other cool thing about the program `mScript_make_result_tables` is that it can assemble multiple group analyses (for each of which you would create a "results1-like" table first) in different worksheets within the spreadsheet. You can thus create one file (let's call it `ResultsForPaperX`) with multiple "tabs" or worksheets, one for each group analyses. For example, the first tab of this filecould be group differences and the second could be correlations with a behavioral measure. Here is an example of how you would make a Makefile to do that (this is not available in the examples directory).

```
SHELL=/bin/bash
export SHELL
.PHONY:
all: GroupDifferences Correlations ResultsForPaperX

GroupDifferences.txt: groupDifferencesAnalysis.gfeat
        bin/mScript_Get_feat_stats $< ;\
        mv groupDifferencesAnalysis.gfeat_Feat_Results $@

Correlations.txt: correlationsAnalysis.gfeat
        bin/mScript_Get_feat_stats $< ;\
        mv correlationsAnalysis.gfeat_Feat_Results $@

❸ ResultsForPaperX.xlsx: GroupDifferences.txt Correlations.txt
        bin/mScript_make_result_tables -o $@ -f $(word 1,$^),$(word 2,$^)
```

Here, we create tables from two group Feat directories (`groupDifferencesAnalysis.gfeat` and `correlationsAnalysis.gfeat`). Because these tables are independent of each other (they only depend on the gfeat directory), you can use the `-j` flag so that they are made in parallel (this is also the time consuming step!). ❸ The file `ResultsForPaperX.xlsx` will be created from the tables `GroupDifferences.txt`

and `Correlations.txt`. Because this target depends on **both** these tables, `make` will wait patiently until both are generated before it runs `mScript_make_result_tables`. Give it a try with your own group Feats!

Example 12

Plotting Group FEAT Results Against Behavioral Measures

Melissa A. Reilly[1]

In neuroimaging research it is often beneficial to examine your results in conjunction with behavioral data to gain a more complete understanding of your effect. For example, it is certainly informative enough to look at group activation maps from subjects completing a memory task within the scanner; however, taking it a step further and incorporating the subjects' accuracy scores on the task arguably paints a richer picture. The example provided here does exactly this using a subset of a dataset from OpenFMRI.org (`https://openfmri.org/dataset/ds000115`); these study subjects completed a memory task (the n-back) within the scanner and their accuracy scores were tracked. In this example, we will visualize each significant cluster, identify it, and plot its activation against the accuracy scores, all in the hopes of providing a clearer interpretation of the data.

The code for this example is in `$MAKEPIPELINES/WorkingMemory`. From within that directory, call the `PrepareExample` script to download the data and organize the directory - please note, this may take upwards of 30 minutes depending on your machine.

```
$ bash PrepareExample
```

Once the download is complete, you can use the `"prepare"` target to run the lower-level FEATs.

```
$ make -f makefiles/Makefile -k -j TARGET=prepare
```

When the lower-level FEATs have completed, you can run the higher-level group analysis. A design file is already prepared for this:

```
$ Feat lib/GROUP_2BACK.fsf
```

If you open the file with Feat, you will note that the accuracy scores are already included (these scores were obtained from `data/demographics.txt`). You should check that the paths to the input files and standard brain are correct on your system. Once you have, click "Go" to run the analysis.

This example uses a `makefile` in several smaller chunks to create the final product. The first part is to read the results from the `.gfeat` directory to generate masks for each significant cluster, and this utilizes `makefiles/Makefile.gfeat`. The second part is to use an FSL tool (`featquery`) to warp each mask into the subject's space and extract from it the mean z-score. We then use the `makefile` to create a spreadsheet within

[1]`mreilly@uw.edu`

the .gfeat directory containing all the featquery z-scores, and finally, plot all those values against n-back performance and create an HTML report containing the plots as well as visualization of the clusters. Let's begin:

```
SHELL=/bin/bash
export SHELL
FSL_DIR=/usr/share/fsl/5.0

ifeq "$(origin MAKEPIPELINES)" "undefined"
MAKEPIPELINES=/project_space/makepipelines
endif

1 group1: .readyforfq
group2: FQresults_DATA
group3: FQreport.html

.PHONY: group1 featqueries group2 group3 clean.gfeat

clean.gfeat:
        rm -rf .readyforfq FQ* design2.mat sed* PLOTS MASKS CLUSTERS AQUERY .
           legend

MYLOCAT=$(shell pwd)
SCRIPTDIR=/project_space/makepipelines/WorkingMemoryIBIC/lib
GF=$(basename $(shell basename `pwd`))
ALLCOPES=$(wildcard cope*.feat)
NUMMASKS=`ls MASKS | wc -l`
```

As you've seen before, we define our SHELL and FSL_DIR variables for consistency. We also include a "clean.gfeat" target to remove any of the outputs generated from this makefile. 1 Makefile.gfeat is run in several small chunks, so the makefile is divided into four phony targets to make this easier to run from the command line (three of the four are defined above, the forth is soon to come). Here we prepare some variables to help make complete the "group1" target, which creates the masks we need before we move into the subject directories. MYLOCAT is a variable to save our current location, and GF pulls the name of the .gfeat out of that. We define SCRIPTDIR to make a note of where we will be keeping some scripts we'll need later. The ALLCOPES variable complies a list of all the cope*.feat subdirectories within the .gfeat. The NUMMASKS variable will list all the masks in our soon-to-be-created MASKS/ subdirectory. The "group1" target has only one prerequisite - .readyforfq.

```
.readyforfq:
        mkdir -p MASKS ;\
        mkdir -p CLUSTERS ;\

    2   for COPE in $(ALLCOPES); do \
            3   Zs=`ls $${COPE} | grep ^cluster_mask_zstat | wc -l` ;\
                WHICHZ=`seq 1 1 $${Zs}` ;\
                for CM in $${WHICHZ}; do \
              4   CNUM=`fslstats $${COPE}/cluster_mask_zstat$${CM}.nii.gz -R | awk
                      '{ print $$2 }'` ;\
                       for VAL in `seq -w 01 01 $${CNUM%%.*}`; do \
                               $(FSL_DIR)/bin/fslmaths $${COPE}/
                                  cluster_mask_zstat$${CM}.nii.gz -thr $${VAL} -
                                  uthr $${VAL} -bin MASKS/$${COPE%.*}_z$${CM}_m$$
                                  {VAL}.nii.gz ;\
                               $(FSL_DIR)/bin/fslmaths $${COPE}/thresh_zstat$${CM
                                  }.nii.gz -mas MASKS/$${COPE%%.*}_z$${CM}_m$${
```

```
                                    VAL}.nii.gz CLUSTERS/$${COPE%.*}_z$${CM}_m$${
                                    VAL}.nii.gz ;\
                        done ;\
                done ;\
        done ;\

        echo "$(NUMMASKS) masks found for $(GF)!";\
        touch .readyforfq
```

We begin by creating `MASKS/` and `CLUSTERS/` subdirectories to house the files we are about to create. We have already defined our `ALLCOPES` variable to be anything in the directory that fits the `cope*.feat` wildcard. In this example we have five copes, which are defined in `GROUP_2BACK/design.fsf`, but a given analysis could have any number, so a wildcard allows the program to be accommodating to both simpler and more complex designs. We want `make` to go into each of these copes (❷), find out how many `cluster_mask_zstat*.nii.gz` images are in each (❸), and then how many unique clusters are in each of those (❹). Though not ideal as far aesthetics are concerned, some shell variables and looping is the simplest way to do this. (It is worth pointing out that the double `$$` is intentional – when referring to shell variables `make` requires that you escape the `$` with another `$`.) For each unique cluster, `fslmaths` will create a binary mask to be placed in the `MASKS/` directory, and will then use that mask to create a thresholded image in the `CLUSTERS/` directory. The images are identical, except that the `MASKS/` one is binarized, and both are named according to the same convention (cope(#)z(#)m(#).nii.gz). To put all this into practice, move into `GROUP_2BACK.gfeat` and run the "group1" target (NOTE: the "group1" target should NOT be parallelized):

```
$ make -f ../makefiles/Makefile.gfeat -k group1
```

As the "group1" target completes, it will print the number of masks created to the terminal. If there are no masks, there is nothing left to do; this analysis has 13 masks, which you can confirm by looking in the `MASKS/` subdirectory. The next step is to pass these masks to Featquery to run in the individual subject directories.

```
INTYPE=`cat design.fsf | grep "set fmri(inputtype)" | awk '{print $$$$3}'`
INPUTS=$(shell cat `pwd`/design.fsf | grep "set feat_files" | awk '{print $$3}' |
   sed -e 's/"//g' | sed -e 's/\.feat.*/\.feat/')
MASKLIST=$(basename $(basename $(shell ls MASKS)))

❺ define query

$(1)/fq_$(GF)_$(2)/report.txt: .readyforfq MASKS/$(2).nii.gz
        if [ $(INTYPE) -eq 1 ]; then COPENUM=`echo $(2) | cut -c 5-5`; else
           COPENUM=1; fi;\
        if [ -d $(1)/fq_$(GF)_$(2) ]; then rm -rf $(1)/fq_$(GF)_$(2)*; fi ;\
        $(FSL_DIR)/bin/featquery 1 $(1) 1 stats/zstat$$$${COPENUM} fq_$(GF)_$(2) $
           (MYLOCAT)/MASKS/$(2).nii.gz ;\
        echo "`cat $(1)/fq_$(GF)_$(2)/report.txt | awk '{print $$$$6}'`"

endef

❻ featqueries: $(foreach mask, $(MASKLIST), $(INPUTS:%=%/fq_$(GF)_$(mask)/report.txt
   ))
❼ $(foreach input,$(INPUTS),$(foreach mask,$(MASKLIST),$(eval $(call query,$(input),
   $(mask)))))

clean.featqueries:
        rm -rfv $(foreach mask, $(MASKLIST), $(INPUTS:%=%/fq_$(GF)_$(mask)))
```

We will first define a few more variables to help us in running the **featqueries** target. The INTYPE variable reads the **.gfeat** design file to determine the input type: lower-level FEATs or cope images. We have already discussed that using shell variables requires an extra $, but this gets even more complicated within a function. Additional escapes are necessary, hence the four $ in the **awk** command. The INPUTS variable reads the subject inputs from the design file, and uses some **sed** commands to remove some extensions and quotation marks. The MASKLIST variable is, of course, a list of all the masks contained in the MASKS/ directory. Since a given group FEAT can have any number of masks, it is imperative that our program is flexible enough to deal with an unspecified number. We need to write a smarter recipe, where **make** will know to take each mask, run **featquery**, and create the output file – and all this without us specifying how many masks or how they are named. **5** To do this, we define a function called **query**. The recipe defined within it is written like any other, but makes use of arguments, which appear as $(1) and $(2). The intent is for **every** subject input ($(1)) and for **every** mask we have generated ($(2)), to run Featquery. Breaking down the recipe itself, the first line reads the INTYPE variable to determine how to define the COPENUM variable, as this is slightly different when using lower-level FEATs as inputs vs. cope images. The second line will check if a Featquery directory already exists for this subject and mask combination, and will delete it if it does. The third line actually runs the Featquery command.

6 The phony "featqueries" target is defined using pattern substitution to be all the **report.txt** files that result from each featquery that is to be run. **7** We use a combination of **make**'s **foreach**, **eval**, and **call** to read all the masks contained in MASKLIST and all the subject inputs and submit each of those as arguments to **query**. We also define a "clean.featqueries" target to clean up all the Featquery outputs in the subject directories, which you may find useful for clutter-control after the spreadsheet has been generated. Run the "featqueries" target (and this time, parallelizing is encouraged!).

```
$ make -f ../makefiles/Makefile.gfeat -k -j featqueries
```

When all the featqueries have completed, we are ready for the "group2" target:

```
RESULTS=$(MASKLIST:%=FQresults_%)
INPUTNUM=`cat design.fsf | grep "set feat_files(" | wc -l`
```

There are only a few additional variables we need for completing the "group2" target, which compiles the spreadsheet (FQresults_DATA) containing the subject list, explanatory variables (EVs) and the mean values for each mask. INPUTNUM is a count of our subject inputs, which we are reading from the **design.fsf** file. RESULTS will hold the names of the files that will contain each column of the Featquery data in the spreadsheet.

```
FQresults_DATA: .readyforfq FQresults_allSUBS FQresults_EVs $(RESULTS)
	paste -d',' FQresults_allSUBS FQresults_EVs $(RESULTS) > FQresults_DATA
```

FQresults_DATA will be a comma-separated spreadsheet that contains a column of the subject IDs, the names of the EVs and their values for each subject, and the names of the masks and the mean z-scores extracted from each of them for each subject. This file is created by pasting together several smaller files which are created below.

```
FQresults_allSUBS: .readyforfq
	echo $(INPUTNUM)_SUBJECTS >> FQresults_allSUBS ;\
	echo $(INPUTS) >> FQresults_allSUBS ;\
	sed -i 's/ /\n/g' FQresults_allSUBS ;\
	rm -f sed*
```

FQresults_allSUBS is a text file containing each subject input. This file will be the first column of the FQresults_DATA spreadsheet. We simply print "$(INPUTNUM)_SUBJECTS" to the file to serve as the column header. We **echo** the subject inputs (the contents of $(INPUTS)) after that, and use **sed** to replace spaces with linebreaks.

```
FQresults_EVs: .readyforfq
```

```
        NUMEV=`cat design.mat | awk '/NumWaves/ {print $$2}'` ;\
    8   for EV in `seq 1 1 $${NUMEV}`; do \
                echo -ne "`cat design.fsf | grep "set fmri(evtitle$${EV})" | awk
                    '{print $$3}'`," >> FQresults_EVs ;\
        done ;\
        echo >> FQresults_EVs ;\
        sed -i 's/"//g' FQresults_EVs ;\
        cat design.mat | tail -n $(INPUTNUM) >> design2.mat ;\
        NUMEV=`cat design.mat | awk '/NumWaves/ {print $$2}'` ;\
        sed -i 's/\t/,/g' design2.mat ;\
        cat design2.mat >> FQresults_EVs ;\
        sed -i 's/,$$//g' FQresults_EVs ;\
        sed -i 's/\"//g' FQresults_EVs ;\
        rm -f sed*
```

`FQresults_EVs` is another text file that will make up the column(s) immediately to the right of the subject inputs in `FQresults_DATA`, with the number of columns being determined by how many EVs were included in your `.gfeat` model (in our case, two). This recipe reads the `.gfeat`'s `design.mat` and `design.fsf` files, which contain all the information we need. Our end goal is to have a column for each EV where the EV name is printed in the first row and the value for each subject is printed on the subsequent lines, with each column being separated from the next with a comma. The `NUMEV` variable will read the header of the `design.mat` file to determine the number of EVs. **8** We will then use a simple `for loop` to read the names of the EVs one through `$(NUMEV)` from `design.fsf`, and print all those names to the first row of `FQresults_EVs`. To obtain the values for these EVs, we essentially want the information contained in `design.mat` (but not all of it, and not in that format). We will copy the lines of `design.mat` that pertain to the individual subjects to `design2.mat` by using the `tail` command in conjunction with our `INPUTNUM` variable. A series of `sed` commands at the end of the recipe cleans up the data: the first exchanges tabs for commas, the second removes commas at the end of a line, and the last removes quotes that `FEAT` puts around the values. `FQresults_EVs` is now ready to be pasted next to `FQresults_allSUBs` in the creation of `FQresults_DATA`; we need only the columns that correspond to the individual clusters:

```
define getvalues

FQresults_$(1): .readyforfq
        echo "$(1)" > temp_$(1) ;\
        for i in $(INPUTS); do echo `cat $$$${i}/fq_$(GF)_$(1)/report.txt | awk '{
            print $$$$6}'` >> temp_$(1); done ;\
        mv temp_$(1) FQresults_$(1)

endef

9 $(foreach mask,$(MASKLIST),$(eval $(call getvalues,$(mask))))
```

As you well know by now, a function is our best bet for dealing with the unpredictable nature of the number of masks in our analysis. The `getvalues` function will create a text file for each mask called "`FQresults_-MaskName`", which will have the name of the mask in the first row and the values for each subject in subsequent rows. **9** As we did with the `query` function, we submit each mask name as an argument. For each mask, the recipe looks into each subjects corresponding `featquery` results page, grabs the mean value, and prints it to the `FQresults` file for that mask.

Despite the lengthy explanation, this all runs very quickly. Run the "group2" target; you should not include a `-j` flag.

```
$ make -f ../makefiles/Makefile.gfeat group2 -k
```

Completion of the "group2" target yields a comma-separated spreadsheet that you can import to statistical or graphing software, but make still has a few more tricks up its sleeve. The "group3" target is by far the most impressive part of this example, so let's jump right in:

```
INTYPE2=`cat design.fsf | grep "set fmri(inputtype)" | awk '{print $$3}'`
WHICHX=$(shell head -n 1 FQresults_EVs | awk -F ',' '{print $$NF}')
PAGES=$(MASKLIST:%=AQUERY/%)
STANDARD=/usr/share/fsl/5.0/data/standard/MNI152_T1_2mm_brain.nii.gz
```

We define just a few new variables to complete the "group3" target. The INTYPE2 variable is similar to the INTYPE variable we defined earlier, different only in the number of escapes in the awk command, because we won't be using it within a function this time. When we call R to read FQresults_DATA and create the scatterplots, we need to specify which variable goes on the x-axis. For simplicity, it is assumed that the last EV entered is intended to be the x-axis variable, and thus WHICHX is defined to be the last item in the first row of FQresults_EVs. PAGES represents the names of the small chunks of HTML we will put together for each cluster we intend to display in our final report, which will be saved in a subdirectory called AQUERY/. STANDARD contains the full path to our standard brain, which should be modified to match your system.

```
Rplots: FQresults_DATA
        mkdir -p PLOTS ;\
        Rscript $(SCRIPTDIR)/AUTOPLOT.R $(WHICHX) FQresults_DATA $(shell pwd) ;\

define ssclust

PLOTS/$(1)_brain.png: .readyforfq
        echo $(1) ;\
        mkdir -p PLOTS ;\
        /bin/bash $(SCRIPTDIR)/sscluster $(1) $(FSL_DIR) $(STANDARD)

endef

$(foreach cluster,$(MASKLIST),$(eval $(call ssclust,$(cluster))))
```

The phony "Rplots" target will create a directory to house some of the images we're about to create, and will call the R script (located in the lib/ directory) to create the scatterplots. Each plot will be saved according to the mask name. Within the ssclust function we will call an external script to create a screenshot of each cluster to view alongside the corresponding scatterplot, saved as PLOTS/MaskName_brain.png. These screenshots will overlay the cluster on the specified standard brain, and will capture ten images in each plane to create a visual representation of each cluster.

```
define atlasquery

AQUERY/$(1): .readyforfq $(MASKLIST:%=PLOTS/%_brain.png) Rplots .legend
        mkdir -p AQUERY ;\
        HTML=AQUERY/$(1) ;\
        echo "<img src="PLOTS/$(1).png" alt="$(1).png">" >> $$$${HTML} ;\
        echo "<img src="PLOTS/$(1)_brain.png" alt="$(1)_brain.png" width="60%">"
           >> $$$${HTML} ;\
        echo "<br><br><table width="100%"><tr>" >> $$$${HTML} ;\
        HOCORT=`$(FSL_DIR)/bin/atlasquery -a "Harvard-Oxford Cortical Structural
           Atlas (Lateralized)" -m CLUSTERS/$(1)` ;\
        echo "<td valign="top"><p align="left">Harvard-Oxford Cortical Structural
           Atlas:<br><small>`echo $$$${HOCORT} | sed -e 's/[0-9] /<br>/g'`</small
           ></p></td>" >> $$$${HTML} ;\
        HOSUBCORT=`$(FSL_DIR)/bin/atlasquery -a "Harvard-Oxford Subcortical
           Structural Atlas" -m CLUSTERS/$(1)` ;\
```

```
	echo "<td valign="top"><p align="left">Harvard-Oxford Subcortical Structural Atlas:<br><small>`echo $$$${HOSUBCORT} | sed -e 's/[0-9] /<br>/g'`</small></p></td>" >> $$$${HTML} ;\
	CEREBELL=`$(FSL_DIR)/bin/atlasquery -a "Cerebellar Atlas in MNI152 space after normalization with FLIRT" -m CLUSTERS/$(1)` ;\
	echo "<td valign="top"><p align="left">Cerebellar Atlas after normalization with FLIRT:<br><small>`echo $$$${CEREBELL} | sed -e 's/[0-9] /<br>/g'`</small></p></td>" >> $$$${HTML} ;\
	JUEL=`$(FSL_DIR)/bin/atlasquery -a "Juelich Histological Atlas" -m CLUSTERS/$(1)` ;\
	echo "<td valign="top"><p align="left">Juelich Histological Atlas:<br><small>`echo $$$${JUEL} | sed -e 's/[0-9] /<br>/g'`</small></p></td>" >> $$$${HTML} ;\
	echo "</tr></table>" >> $$$${HTML} ;\
	echo "<p>- - - - - - - - - - - - - - - - - - - - - - - -</p>" >> $$$${HTML}

endef

$(foreach cluster,$(MASKLIST),$(eval $(call atlasquery,$(cluster))))
```

The final FQreport.html file is built from a number of smaller pieces, and the atlasquery function will create most of these pieces. We first create a AQUERY/ subdirectory to put all these pieces in, where "AQUERY" is short for atlasquery, an FSL tool that gives the average probability of a mask being a member of the different labeled regions of a given atlas. The small pieces of HTML we are compiling here will, for all of our masks, place the scatterplot next to the corresponding cluster screenshot, and below that, will provide the atlasquery outputs for four different atlases: the Juelich Histological Atlas, Cerebellar Atlas in MNI152 space after normalization with FLIRT, and the Harvard-Oxford Cortical and Subcortical Structural Atlases. These individual pieces can be viewed on their own, but we will pull them all together to create the FQreport.html file:

```
FQreport.html: $(MASKLIST:%=AQUERY/%)
	REPORT=FQreport.html ;\
	echo "<!DOCTYPE html><html>" > $${REPORT} ;\
	echo "<head>" >> $${REPORT} ;\
	echo "<title>$(GF).gfeat</title>" >> $${REPORT} ;\
	echo "</head>" >> $${REPORT} ;\
	echo "<body bgcolor="#000000"><font face="Arial" color="#ffffff">" >> $${REPORT} ;\
	echo "<center>" >> $${REPORT} ;\
	echo "<p>Scatter plots depicting the relationship between brain activity and behavioral measures are provided for descriptive purposes only</p>" >> $${REPORT} ;\
10	for page in $(PAGES); do echo "<BR><BR>">> $${REPORT}; cat $${page} >> $${REPORT}; done ;\
	echo "</center></body></html>" >> $${REPORT}
```

The last bit of code here serves as a template for the HTML report. It contains code pertaining to basic styling and formatting of the HTML document, and 10 then looks to $(PAGES) to get the names of the HTML pieces contained in the AQUERY/ subdirectory, and embeds those into this report. To run this final bit, you should feel free to parallelize:

```
$ make -f ../makefiles/Makefile.gfeat group3 -k -j
```

An example of the final report is shown below (for a clearer view, a pre-generated copy is saved at lib/pregenREPORT.html; it provides a clear description of the data, and together with the FQresults_DATA

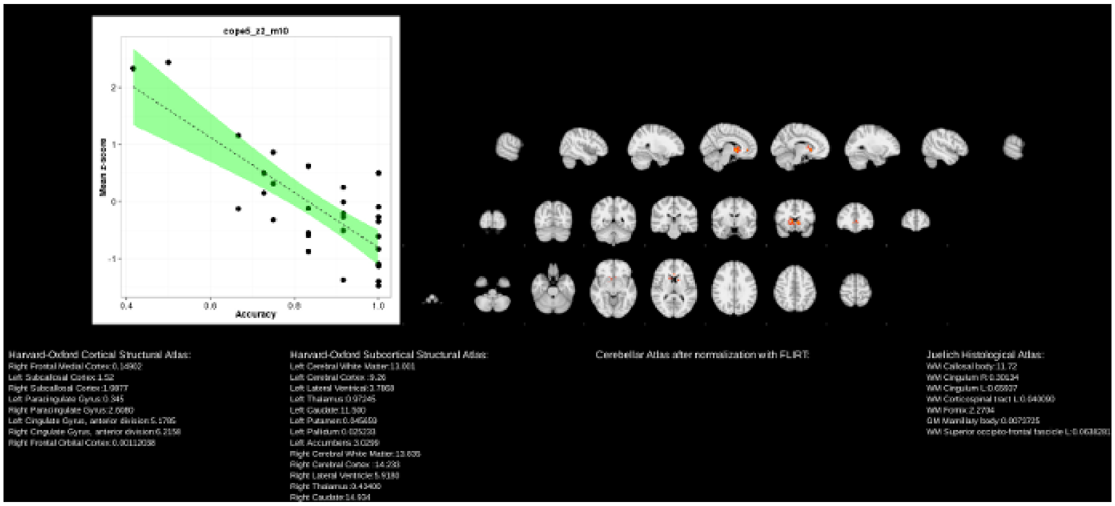

Figure 12.1: Report plotting group FEAT results against behavioral measures.

file, provides an excellent stepping stone for further analysis.

Bibliography

Alsop, D., Detre, J., Golay, X., GÃijnther, M., Hendrikse, J., Hernandez-Garcia, L., Lu, H., Macintosh, B., Parkes, L., Smits, M., van Osch, M., Wang, D., Wong, E., and Zaharchuk, G. (2015). Recommended implementation of arterial spinâĂŘlabeled perfusion mri for clinical applications: A consensus of the ismrm perfusion study group and the european consortium for asl in dementia. *Magnetic Resonance in Medicine*, 73(1):101–116.

Avants, B. B., Tustison, N. J., Stauffer, M., Song, G., Wu, B., and Gee, J. C. (2014). The insight toolkit image registration framework. *Frontiers in Neuroinformatics*, 8:44.

Desikan, R. S., Ségonne, F., Fischl, B., Quinn, B. T., Dickerson, B. C., Blacker, D., Buckner, R. L., Dale, A. M., Maguire, R. P., Hyman, B. T., Albert, M. S., and Killiany, R. J. (2006). An automated labeling system for subdividing the human cerebral cortex on mri scans into gyral based regions of interest. *NeuroImage*, 31(3):968 – 980.

Fischl, B., Salat, D. H., van der Kouwe, A. J., Makris, N., Ségonne, F., Quinn, B. T., and Dale, A. M. (2004a). Sequence-independent segmentation of magnetic resonance images. *NeuroImage*, 23(Supplement 1):S69 – S84. Mathematics in Brain Imaging.

Fischl, B., van der Kouwe, A., Destrieux, C., Halgren, E., Ségonne, F., Salat, D. H., Busa, E., Seidman, L. J., Goldstein, J., Kennedy, D., Caviness, V., Makris, N., Rosen, B., and Dale, A. M. (2004b). Automatically Parcellating the Human Cerebral Cortex. *Cerebral Cortex*, 14(1):11–22.

Gorgolewski, K., Burns, C. D., Madison, C., Clark, D., Halchenko, Y. O., Waskom, M. L., and Ghosh, S. S. (2011). Nipype: A flexible, lightweight and extensible neuroimaging data processing framework in python. *Frontiers in Neuroinformatics*, 5:13.

Graham-Cumming, J. (2015). *The GNU Make Book*. No Starch Press.

Iglesias, J., Liu, C., Thompson, P., and Zu, T. (2011). Robust brain extraction across datasets and comparison with publicly available methods. *IEEE Transactions on Medical Imaging*, 30(9):1617–1634.

Jenkinson, M., Beckmann, C. F., Behrens, T. E., Woolrich, M. W., and Smith, S. M. (2012). Fsl. *Neuroimage*, 62(2):782–790.

Lee SE, Khazenzon AM, e. a. (2014). Altered network connectivity in frontotemporal dementia with c9orf72 hexanucleotide repeat expansion. *Brain*, 137:3047–60.

Power, J. D., Barnes, K. A., Snyder, A. Z., Schlaggar, B. L., and Petersen, S. E. (2012). Spurious but systematic correlations in functional connectivity MRI networks arise from subject motion. *NeuroImage*, 59(3):2142–2154.

Seeley WW, Crawford R, e. a. (2008). Frontal paralimbic network atrophy in very mild behavioral variant frontotemporal dementia. *Arch Neurol*, 65:249–55.

Stein, J. L., Medland, S. E., Vasquez, A. A., Hibar, D. P., Senstad, R. E., Winkler, A. M., Toro, R., Appel, K., et al. (2012). Identification of common variants associated with human hippocampal and intracranial volumes. *Nature Genetics*, 44(5):552–561.
